# The yeast Mkt1/Pbp1 complex promotes adaptive responses to respiratory growth

**DOI:** 10.1083/jcb.202411169

**Published:** 2025-08-13

**Authors:** Daniel Caballero, Benjamin M. Sutter, Zheng Xing, Caroline Wang, Emma Choo, Yun Wang, Yu-San Yang, Sina Ghaemmaghami, Andrew Lemoff, Benjamin P. Tu

**Affiliations:** 1 Department of Biochemistry, University of Texas Southwestern Medical Center, Dallas, TX, USA; 2 Howard Hughes Medical Institute, University of Texas Southwestern Medical Center, Dallas, TX, USA; 3 Department of Biology, University of Rochester, Rochester, NY, USA

## Abstract

An amino acid polymorphism in the Rad2/XPG protein Mkt1 (Mkt1-G30D) reportedly underlies variation in mitochondrial phenotypes among laboratory yeast, but the function of Mkt1 and the effects of the polymorphism are unknown. We confirm with genetics and biochemical assays guided by AlphaFold structure predictions that Mkt1 forms a complex with Pbp1, a messenger RNP protein that supports adaptations to respiratory conditions, such as Pumilio protein Puf3-dependent mitochondrial protein expression and TORC1-dependent autophagy. Using CEN.PK (Mkt1-G30) yeast, we show that, like Pbp1, Mkt1 is required for Puf3-dependent mitochondrial protein expression and autophagy during respiratory growth. Notably, we found the Mkt1-G30D mutation destabilizes the Mkt1/Pbp1 complex, helping to explain its loss-of-function effects. A *HAP1*^*+*^ S288C strain exhibited defects in mitochondrial biogenesis and autophagy, which were rescued by replacing its Mkt1-D30 allele with the Mkt1-G30 allele. Thus, the Mkt1/Pbp1 complex supports adaptive processes during respiratory growth, and the Mkt1-G30D mutation is an evolutionary adaptation that tempers respiratory processes by destabilizing the Mkt1/Pbp1 complex.

## Teaser

Caballero et al. show that a polymorphism in the Mkt1 gene underlies complex genetic phenotypes among strains of budding yeast. Mkt1 interacts with a stress granule protein Pbp1 to regulate expression of mitochondrial proteins, TORC1 signaling, and autophagy in yeast.

## Introduction

The brewing yeast *Saccharomyces cerevisiae* has been an important model organism for the study of metabolic pathways for decades. Yeast vigorously consume glucose via glycolysis (i.e., fermentation) and grow rapidly in its presence. Depletion of glucose from the environment forces yeast to rely on mitochondrial energy production via the TCA cycle to consume byproducts of glycolysis, such as ethanol and acetate. Adaptation to respiratory conditions involves regulatory changes across various cellular processes, such as transcription, translation, and autophagy. For instance, transcriptional activators such as the HAP2/3/4 complex specifically drive respiratory gene expression upon glucose deprivation (Buschlen et al., 2003). At the level of translation, the Pumilio protein Puf3 promotes either the decay or translation of its target mRNAs, which encode mitochondrial proteins, in a manner dependent on the metabolic state of the cell (Lee and Tu, 2015). Furthermore, we have shown yeasts are capable of undergoing TORC1-regulated autophagy when cultured in a minimal medium that contains nitrogen and the non-fermentable carbon source lactate (Wu and Tu, 2011). Thus, respiratory growth is associated with the induction of specific regulatory programs and processes that collectively rewire and support energy production from non-fermentable carbon sources.

Poly(A) binding protein-binding protein 1 (Pbp1), known as ataxin-2 in higher organisms, is a cytoplasmic RNA-binding protein which is thought to have various roles in posttranscriptional regulation such as poly(A) tail length control, translation, and stress granule formation (Mangus et al., 1998; Mangus et al., 2004; Tadauchi et al., 2004; Swisher and Parker, 2010). In yeast, Pbp1 has also been shown to be important for maintaining mitochondrial function and homeostasis. Pbp1 overexpression has been reported to suppress survival defects resulting from defective intron splicing, ethidium bromide treatment, and expression of a mutant mitochondrial ADP/ATP carrier (Waldherr et al., 1993; Dunn and Jensen, 2003; Wang and Chen, 2015). More recently, we have reported Pbp1 is required for promoting the translation of Puf3-target mRNA transcripts, which generally encode mitochondrial ribosomal and OXPHOS-associated proteins during respiratory growth (van de Poll et al., 2023). Pbp1 overexpression also represses TORC1 signaling during heat stress, and we have additionally shown Pbp1 promotes autophagy during growth in minimal respiratory medium by forming redox-sensitive condensates which repress TORC1 signaling (Takahara and Maeda, 2012; Yang et al., 2019; Kato et al., 2019).

Maintenance of K2 killer toxin 1 (Mkt1) is a poorly understood member of the Rad2/XPG family of DNA repair enzymes and was initially reported in 1980 by Reed Wickner to be involved in the maintenance of viral M2 dsRNA (Wickner, 1980). Over two decades later, Mkt1 was reported to function in a complex with Pbp1 to promote the expression of the HO endonuclease in a manner dependent on the 3′ UTR region of the HO transcript (Tadauchi et al., 2004). More recently, studies in *Trypanosoma brucei* have proposed Mkt1 and Pbp1 exist in a complex which, in association with cytoplasmic mRNA–ribonucleoprotein complexes, appears to be important for mRNA stabilization (Singh et al., 2014; Melo do Nascimento et al., 2020). Interestingly, a polymorphism at the 30th aa residue of yeast Mkt1 (Mkt1-G30D) has also been reported to underlie defects across a range of stress-related processes such as heat tolerance, sporulation, and various mitochondrial-related phenotypes, including petite colony growth, growth in low glucose liquid medium, ethanol tolerance, and levels of mRNAs targeted by the Pumilio protein Puf3 (Steinmetz et al., 2002; Deutschbauer and Davis, 2005; Dimitrov et al., 2009; Anderson et al., 2010; Swinnen et al., 2012; Lee et al., 2009). These reports collectively point to an important role for Mkt1 in promoting mitochondrial function, and indeed, one report even proposed Mkt1 is a “global regulator of mitochondrial mRNAs” (Anderson et al., 2010). However, despite these studies, our knowledge of yeast Mkt1/Pbp1 complex function and the effects of the Mkt1-G30D polymorphism remain limited.

In this study, we provide evidence that Mkt1 exists in a complex with Pbp1 which promotes Puf3-dependent mitochondrial protein expression during respiratory growth and autophagy during nutritional stress under respiratory conditions. We show for the first time that the Mkt1-G30D polymorphism decreases Mkt1/Pbp1 complex abundance and formation, leading to defective Puf3-dependent protein expression and autophagy. Importantly, we find that expression of WT Mkt1-G30 alleles in a *HAP1*^*+*^ S288C strain, which naturally harbors the Mkt1-D30 allele, is sufficient to rescue defects in Puf3-dependent mitochondrial protein expression and autophagy, indicating the Mkt1-G30D polymorphism largely underlies the reduced capacity of S288C yeast to carry out these processes. We define the Mkt1/Pbp1 complex as a key regulator of mitochondrial homeostasis and provide evidence that the Mkt1-G30D polymorphism represents an evolutionary adaptation that supports yeast survival in glucose-rich laboratory environments that favor fermentative growth.

## Results

### Mkt1 and Pbp1 exist in a complex under fermentative and respiratory conditions

**Figure 1.**
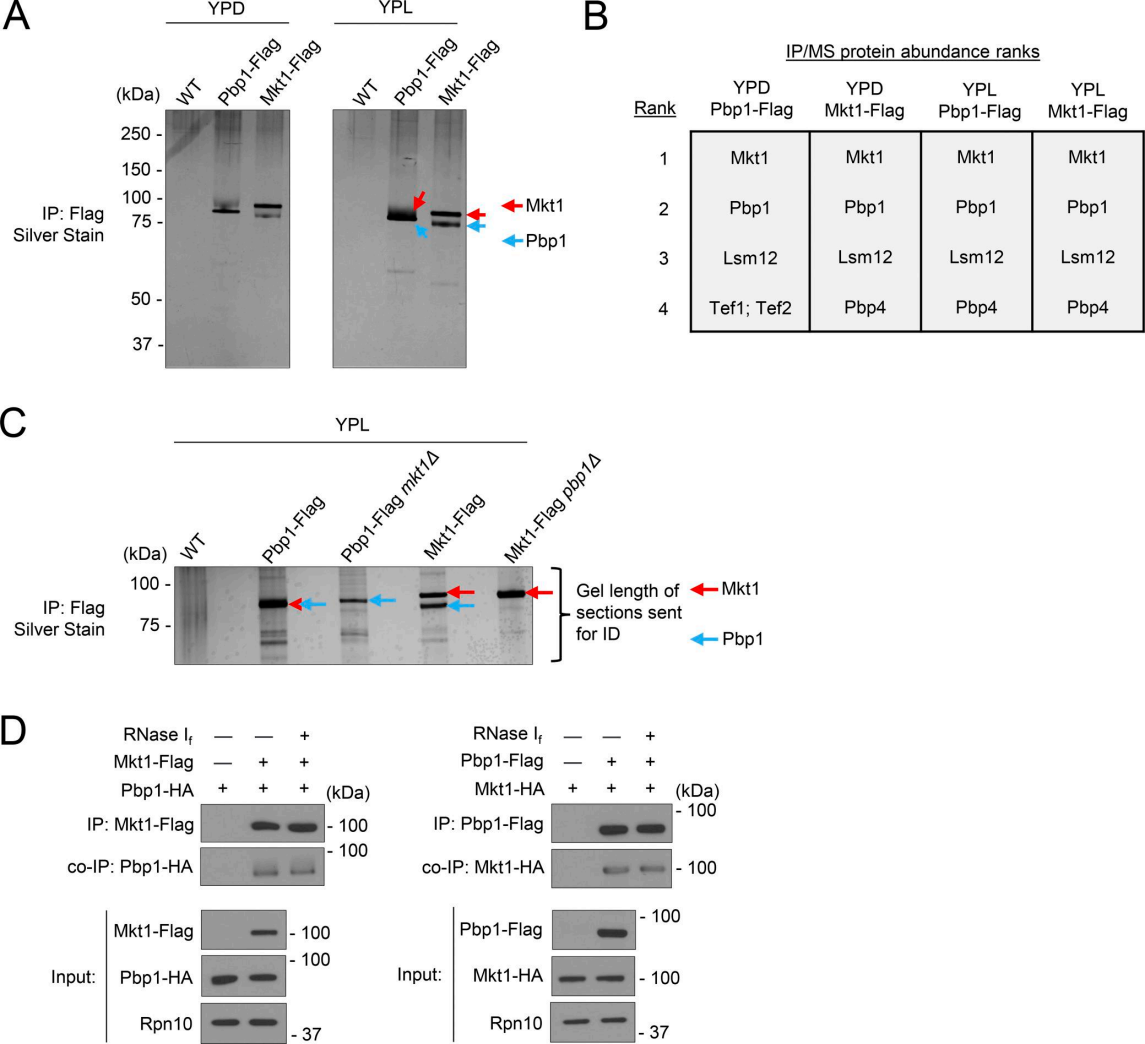
**Mkt1 and Pbp1 form a complex under fermentative and respiratory conditions. (A)** Silver stain of immunoprecipitated Flag-tagged Mkt1 and Pbp1 protein samples used in IP/MS experiment to identify protein interactors. Cells were collected from YPD cultures in log phase and 3 h following switch to YPL medium. **(B)** Top four most abundant proteins in each IP/MS sample from A. **(C)** IP/MS experiment to identify protein bands present on silver stain gel of Mkt1-Flag and Pbp1-Flag IP samples. Length of gel sections cut and sent for protein identification is depicted. Cells were collected from YPL cultures grown to log phase. **(D)** co-IP experiment showing Mkt1–Pbp1 interaction is not dependent on RNA. IP samples were incubated with RNase I_f_ for 15 min at 37°C. Cells were collected from YPL cultures grown to log phase. Source data are available for this figure: SourceData F1.

Pbp1 is thought to associate with cytoplasmic RNA processing and translation factors such as Pab1, Dhh1, Lsm12, and Pbp4 (Mangus et al., 1998; Fleischer et al., 2006; Swisher and Parker, 2010). Mkt1, a poorly understood Rad2/XPG family protein, has previously been reported to interact with Pbp1 in *S. cerevisiae*, *Schizosaccharomycespombe*, *T. brucei*, and *Cryptococcusneoformans*, but biochemical and structural evidence for a complex between Mkt1 and Pbp1 has been lacking (Tadauchi et al., 2004; Taglini et al., 2020; Singh et al., 2014; Son et al., 2019). To identify Mkt1 and Pbp1 protein interactors, we performed an immunoprecipitation/mass spectrometry (IP/MS) experiment using Mkt1-Flag and Pbp1-Flag yeast strains cultured in rich glucose (YPD, fermentative) and lactate (YPL, respiratory) media. In each Flag-tagged sample tested, we found Mkt1 and Pbp1 were the two most abundant proteins present (Fig. 1, A and B; and Table S1). Based on protein sizes of the tagged and untagged proteins in each sample, we reasoned the top and bottom bands present in the Mkt1-Flag lanes were Mkt1 and Pbp1, respectively, while the single band in the Pbp1-Flag lanes consisted of both Mkt1 and Pbp1 proteins. We confirmed the bands were Mkt1 and Pbp1 in a second IP/MS experiment in which protein bands representing Flag-tagged Mkt1 and Pbp1 were cut from a silver-stained gel and identified by MS (Fig. 1 C and Table S2). Finally, we determined the interaction between Mkt1 and Pbp1 was not dependent on RNA by performing a co-IP experiment with RNase treatment (Fig. 1 D). In sum, we obtained evidence that Mkt1 and Pbp1 form a complex under both fermentative and respiratory conditions.

### Characterization of the Mkt1-binding region on Pbp1

**Figure 2.**
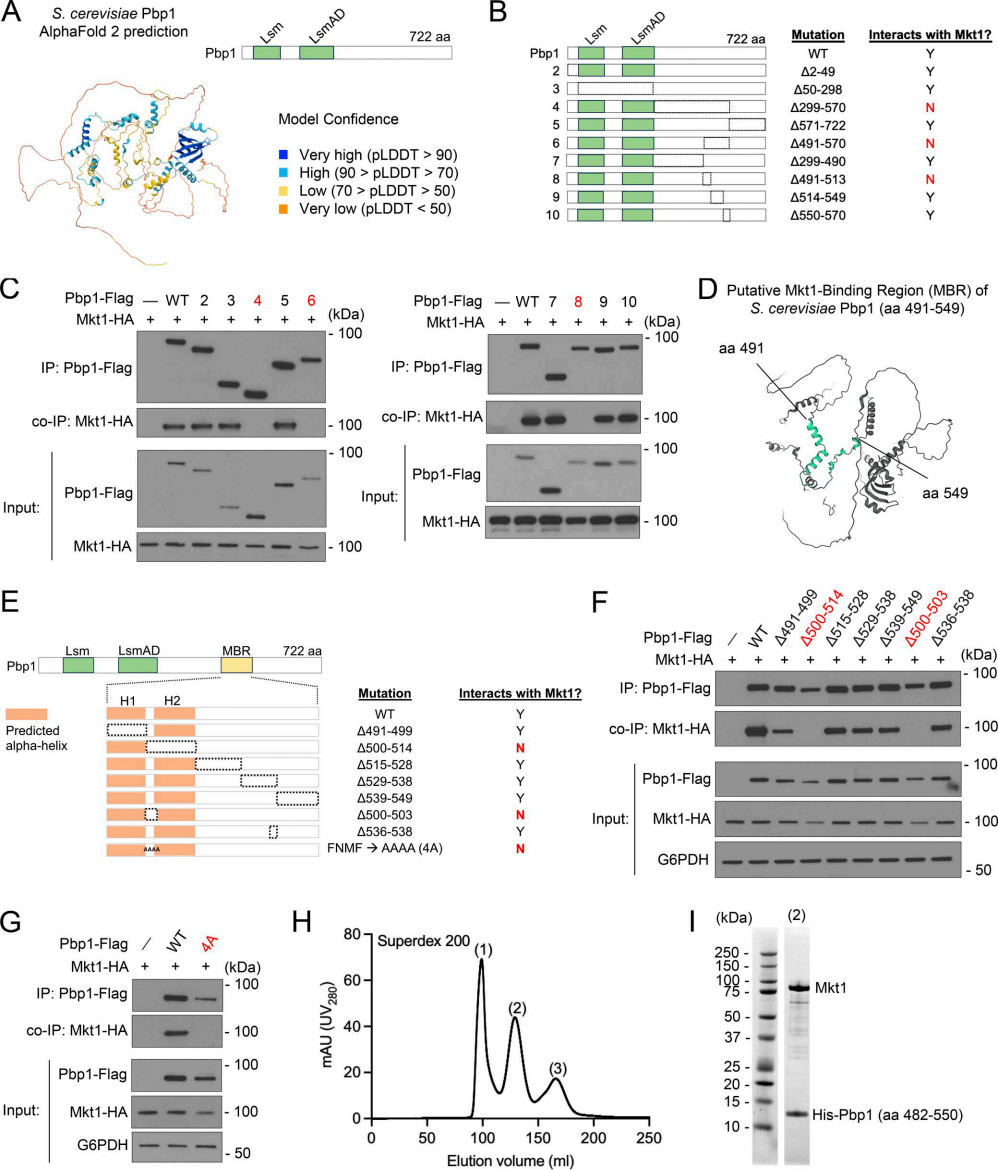
**Evidence for an MBR within Pbp1. (A)** AlphaFold protein structure prediction of *S. cerevisiae* Pbp1 (AF-P53297-F1-v4) and protein domain schematic. **(B)** Schematic representation of Pbp1 truncation mutants assayed for their ability to interact with Mkt1 in C. The dotted lines represent the deleted regions. **(C)** co-IP experiments testing interactions between Pbp1 truncation variants shown in B and Mkt1. Cells expressing each mutant version of Pbp1 with Flag from the endogenous locus and Mkt1-HA were collected at log phase during growth in YPL medium. Note: Pbp1Δ299-570, Pbp1Δ491-570, and Pbp1Δ491-513 did not interact with Mkt1. **(D)** AlphaFold structure depicting *S. cerevisiae* Pbp1’s putative MBR (aa 491–549). **(E)** Schematic representation of Pbp1 truncation mutants assayed for their ability to interact with Mkt1 in F and G. Truncations were made within aa 491–549, which encompasses the proposed MBR. Two helical structures are predicted to reside within the MBR and are represented as orange rectangles. The Pbp1-4A strain has aa 500–503 replaced with four alanine residues. The dotted lines represent the deleted regions. **(F)** co-IP testing interactions between Pbp1 truncation variants depicted in E and Mkt1. Cells expressing each mutant version of Pbp1 with Flag from the endogenous locus and Mkt1-HA were collected at log phase during growth in YPL medium. Pbp1Δ500-514 and Pbp1Δ500-503 did not interact with Mkt1. **(G)** co-IP testing interaction between Pbp1-4A variant depicted in E and Mkt1. Cells were collected at log phase during growth in YPL medium. Pbp1-4A did not interact with Mkt1. **(H)** Full-length Mkt1 and His-Pbp1 (aa 482–550) were co-expressed in Rosetta cells and purified by immobilized metal ion affinity chromatography. Eluted complex was further purified by gel filtration using a HiPrep 26/60 Sephacryl S-200 High Resolution column. Depicted is the gel filtration purification trace. **(I)** Coomassie-stained SDS-PAGE analysis of the peak 2 fraction from H representing the complex of Mkt1 and His-Pbp1 (aa 482–550). Source data are available for this figure: SourceData F2.

Pbp1 is defined by Lsm and LsmAD domains in its N-terminal half, which are thought to be sites of RNA and protein binding, and a highly disordered C-terminal half that includes a methionine-rich redox-sensitive disordered region (Yang et al., 2019; Kato et al., 2019). Inspection of the AlphaFold structure of Pbp1 revealed additional predicted regions of structure interspersed throughout Pbp1, including within the LsmAD domain and the middle (aa 299–570) disordered region (Jumper et al., 2021; Varadi et al., 2024) (Fig. 2 A). We speculated these regions may represent uncharacterized sites of binding to protein and/or RNA, including Mkt1.

**Figure S1.**
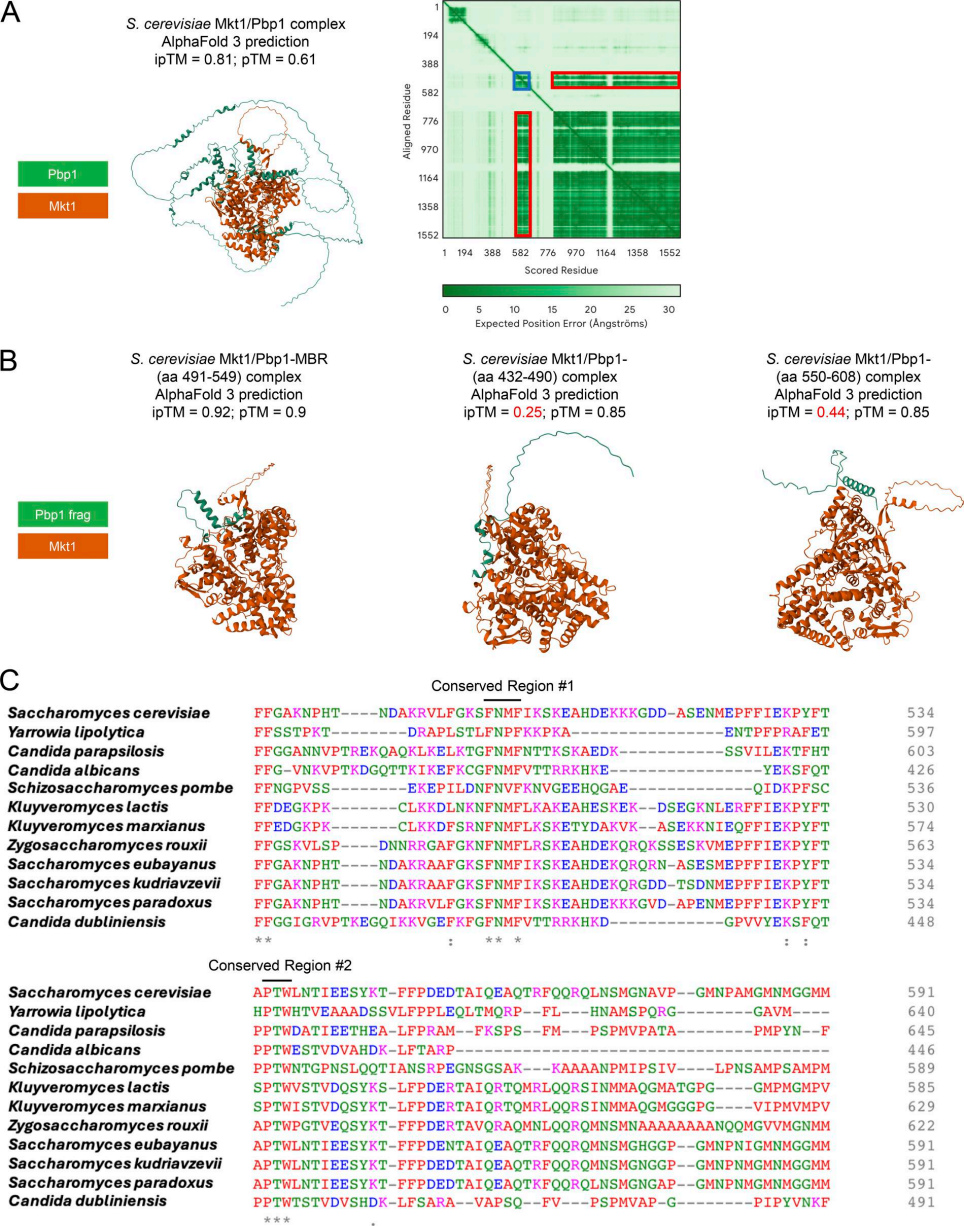
**Protein sequence alignment and AlphaFold analyses of the putative MBR on Pbp1. (A)** AlphaFold 3 complex prediction of *S. cerevisiae* Mkt1 and Pbp1. pTM (0.61) and ipTM (0.81) did not reflect a highly confident interaction, but this was due to Pbp1 protein disordered regions driving down these scores. Highlighted in dark blue on the PAE plot is the predicted Pbp1 structure corresponding to approximately aa 491–549. Highlighted in red are the corresponding ordered parts of Mkt1. PAE plot regions between Pbp1 aa 491–549 and Mkt1 have low expected position error, suggesting a possible interaction (Abramson et al., 2024). **(B)** AlphaFold 3 predictions of *S. cerevisiae* full-length Mkt1 in complex with Pbp1 aa 491–549, aa 432–490, and aa 550–608. pTM and ipTM scores for the Mkt1/Pbp1-MBR (aa 491–549) complex indicated a high-quality prediction that might be similar to the true structure. In the cases of the other two predicted complexes, ipTM scores < 0.6 indicated likely failed predictions. **(C)** Alignment of Pbp1 protein sequences from yeast species generated using Clustal Omega. Two aa stretches in *S. cerevisiae* Pbp1, aa 500–503 and aa 536–538, are highly conserved across yeast species.

To learn more about the Mkt1/Pbp1 complex, we attempted to identify a region of Pbp1 required for binding to Mkt1 with co-IP experiments. Using a collection of Pbp1 truncation mutants, we found that Pbp1 mutants lacking aa 299–570, aa 491–570, and aa 491–513 did not interact with Mkt1 as assessed by co-IP, indicating the region encompassing aa 491–513 may be required for Mkt1-Pbp1 binding (Fig. 2, B and C). The Pbp1 aa 491–513 region does not have an annotated domain, but based on AlphaFold, it is predicted to contain two helical structures, which could represent binding sites. Consistent with this idea, inspection of the PAE plot of the Mkt1/Pbp1 complex AlphaFold 3 prediction revealed Mkt1 is confidently predicted to interact with Pbp1 throughout approximately aa 491–549 (Fig. S1 A) (Abramson et al., 2024). The AlphaFold 3 prediction of Mkt1 and the Pbp1 aa 491–549 region also indicated a highly probable interaction, while predictions between Mkt1 and adjacent Pbp1 regions were not confident (Fig. S1 B). Therefore, based on these data, we propose that Pbp1 aa 491–549 may contain the site of Mkt1 binding, which we term the Mkt1-binding region (MBR) (Abramson et al., 2024) (Fig. 2 D).

To further understand the proposed Pbp1 MBR, we performed a sequence alignment of Pbp1 protein sequences across yeasts. This revealed two conserved regions across the MBR, including a four aa stretch corresponding to Pbp1 aa 500–503, which we speculated could be critical for Mkt1-Pbp1 binding (Fig. S1 C). Indeed, we found Pbp1 truncation strains lacking aa 500–514 and 500–503 were unable to interact with Mkt1 (Fig. 2, E and F). Substitution of aa 500–503 with alanine residues also prevented Mkt1 and Pbp1 from interacting (Fig. 2 G). The loss of Mkt1-Pbp1 binding in these Pbp1 mutants led to clear decreases in both Mkt1 and Pbp1 protein levels, indicating an intact complex is required for normal levels of these proteins (Fig. 2, F and G). Furthermore, we purified a complex comprising full-length Mkt1 and a His-tagged Pbp1 protein fragment containing the putative MBR using immobilized metal ion affinity chromatography followed by gel filtration (Fig. 2, H and I). Taken together, we provide *in vitro* and *in vivo* data that Mkt1 and Pbp1 form a complex.

### Protein sequence and structure prediction analyses of Mkt1

**Figure S2.**
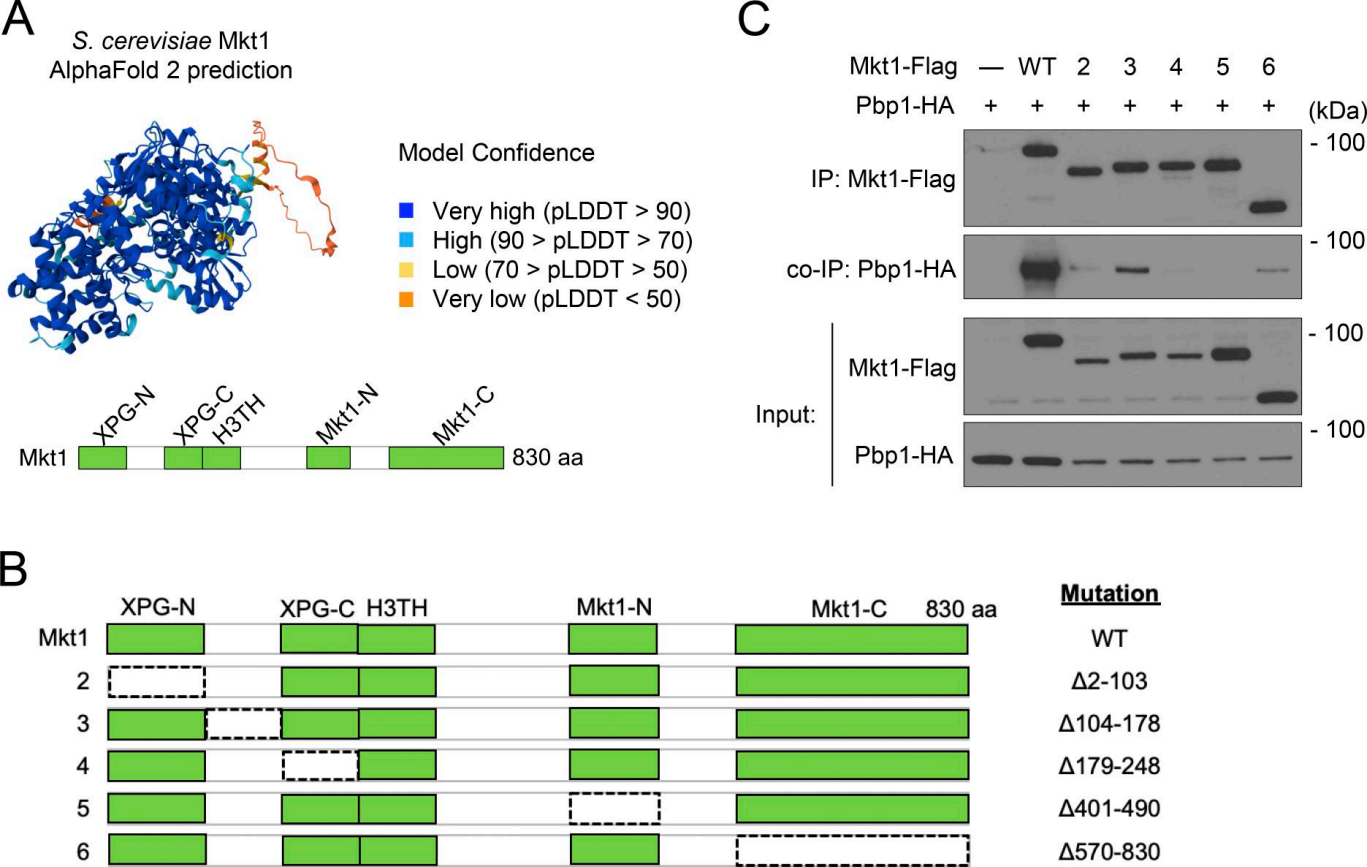
**Interaction and bioinformatic analyses of *S. cerevisiae* Mkt1. (A)** AlphaFold protein structure prediction of *S. cerevisiae* Mkt1 (AF-P40850-F1-v4) and schematic depicting Mkt1 protein domains. **(B)** Schematic representation of Mkt1 truncation mutants assayed for their ability to interact with Pbp1 in C. The dotted lines represent the deleted regions. **(C)** co-IP testing interactions between Flag-tagged Mkt1 truncation mutants and Pbp1-HA. Cells were collected from YPL cultures grown to log phase. All Mkt1 mutants tested had disrupted interactions with Pbp1-HA and reduced Pbp1-HA protein abundance. Source data are available for this figure: SourceData FS2.

Mkt1 contains XPG-N and XPG-C domains characteristic of Rad2/XPG family proteins, a helix-3-turn-helix domain, and Mkt1-N and Mkt1-C domains (Fig. S2 A). Based on its AlphaFold structure, Mkt1 is predicted to be a highly folded protein that resembles other Rad2/XPG proteins (Fig. S2 A) (Jumper et al., 2021; Varadi et al., 2024). The Mkt1 aa 570–830 region corresponding to the Mkt1-C domain was also previously reported to be required for Mkt1 to interact with Pbp1 (Tadauchi et al., 2004). To assess the contribution of other Mkt1 regions toward Pbp1 binding, we created Mkt1 truncation strains lacking other domains and assessed their interactions with Pbp1 in a co-IP experiment (Fig. S2, B and C). All the truncation strains exhibited varying degrees of reduced Mkt1 protein levels, disrupted binding with Pbp1, and similarly decreased Pbp1 protein levels. Overall, it is difficult to determine for each truncation strain whether reductions in co-immunoprecipitated Pbp1 protein were due mainly to reduced Mkt1 protein levels or to the loss of a contacting domain on Mkt1, but they indicate at least that multiple Mkt1 domains are likely binding to Pbp1 (consistent with our AlphaFold 3 complex prediction) and that Pbp1 protein abundance is dependent on an intact complex.

Mkt1 proteins have only been reported in fungi and protozoa, suggesting they are absent in multicellular organisms. We decided to take advantage of the protein structure homology tool Foldseek to determine whether Mkt1-like proteins exist in higher organisms (van Kempen et al., 2024). To our surprise, we found four proteins predicted to be structurally similar to full-length *S. cerevisiae* Mkt1 in *Drosophila*, mice, and humans—asteroid homolog 1 (ASTE1), FAM120A, FAM120B, and FAM120C (Table S3). These proteins contain PIN-like domains and long C-terminal halves like Mkt1 despite exhibiting poor aa sequence conservation with *S. cerevisiae* Mkt1 (Paysan-Lafosse et al., 2023). Therefore, we propose Mkt1 is conserved in higher organisms in the forms of ASTE1 and FAM120 proteins and that together they comprise a previously unrecognized class of proteins within the Rad2/XPG protein family.

### The Mkt1/Pbp1 complex promotes the expression of Puf3-dependent nuclear-encoded mitochondrial proteins

**Figure 3.**
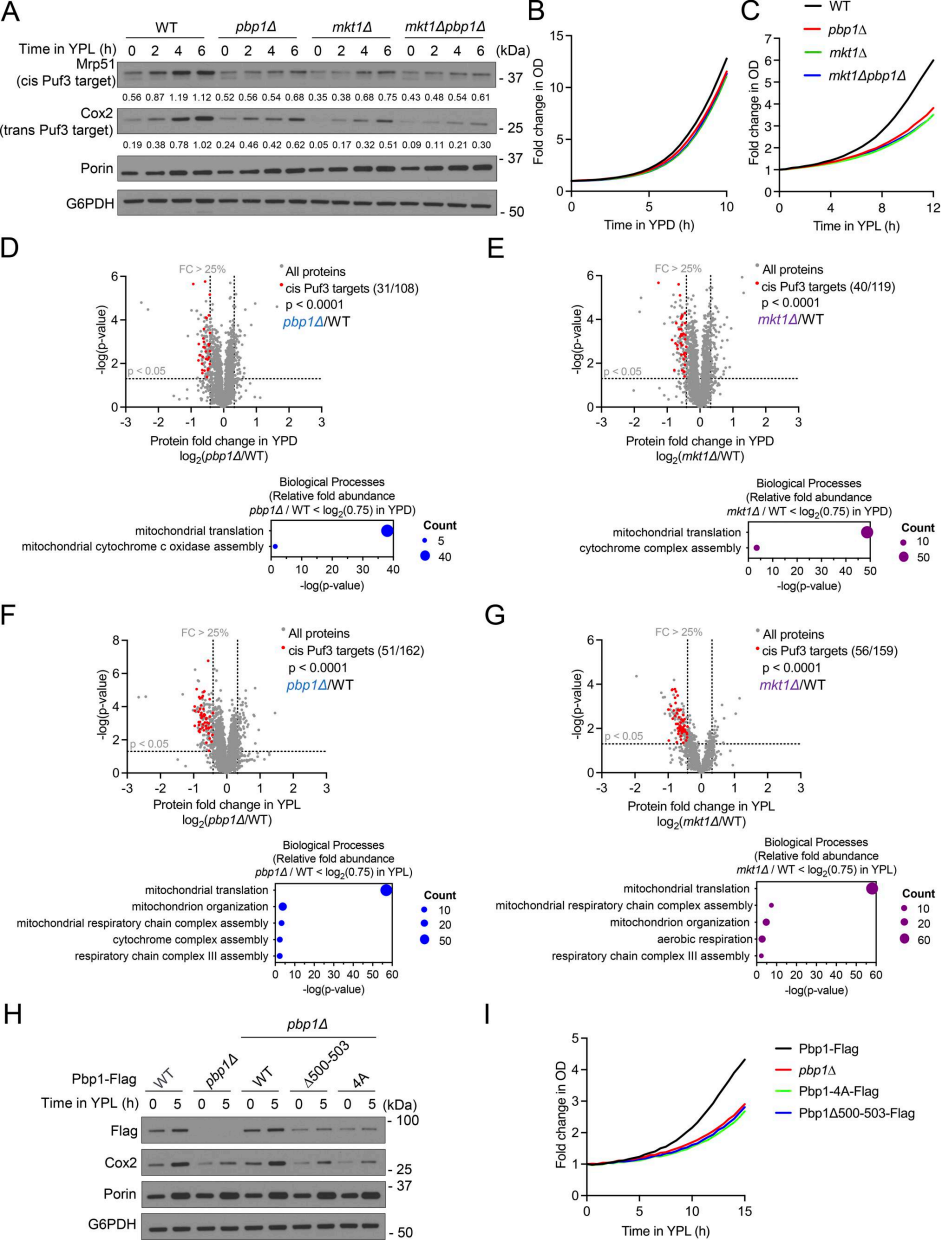
**Mkt1 and Pbp1 are both required for proper translation of nuclear-encoded mitochondrial proteins regulated by Puf3. (A)** Western blot showing levels of the direct Puf3 target Mrp51, the indirect Puf3 target Cox2, and the non-Puf3 target Por1 in indicated strains grown to log phase in YPD and after switch to YPL medium for the indicated times. Numerical values represent relative densities of Mrp51 and Cox2 bands compared with corresponding G6PDH bands. *pbp1∆*, *mkt1∆*, and *mkt1∆pbp1∆* cells exhibited reduced levels of Mrp51 and Cox2 but not Por1. **(B)** Growth curves of indicated strains cultured in YPD medium. Cells were monitored using an automated plate reader during incubation at 30°C, and measurements were collected every 30 min. WT, *pbp1∆*, *mkt1∆*, and *mkt1∆pbp1∆* cells grew at similar rates. Representative traces from a single experiment are depicted (*n* = 6 per group). **(C)** Growth curves of indicated strains grown in YPL medium using the method described in B. *pbp1∆*, *mkt1∆*, and *mkt1∆pbp1∆* cells grew more slowly than WT. Representative traces from a single experiment are depicted (*n* = 6 per group). **(D)** Relative abundances of proteins detected by TMT-MS in *pbp1∆* cells compared with WT during log phase growth in YPD medium (*n* = 3 per group). Statistical significance (P < 0.05) was calculated using unpaired, two-sided *t* test. The set of nuclear-encoded mitochondrial proteins encoded by mRNAs previously shown to be directly regulated by Puf3 (cis Puf3 targets) was significantly reduced based on Fisher’s exact test (Lapointe et al., 2018). Accompanying plot depicts representative mitochondrial protein GO terms enriched among proteins found to be significantly decreased (fold-change < log_2_(0.75)). **(E)** Relative abundances of proteins detected by TMT-MS in *mkt1∆* cells compared with WT during growth in YPD medium (*n* = 3 per group). Statistics were calculated using tests described in D. Accompanying plot depicts representative mitochondrial protein GO terms enriched among proteins found to be significantly decreased (fold-change < log_2_(0.75)). **(F)** Relative abundances of proteins detected by TMT-MS in *pbp1∆* cells compared with WT 3 h following switch from YPD to YPL medium (*n* = 3 per group). Statistics were calculated using tests described in D. Accompanying plot depicts representative mitochondrial protein GO terms enriched among proteins found to be significantly decreased (fold-change < log_2_(0.75)). **(G)** Relative abundances of proteins detected by TMT-MS in *mkt1∆* cells compared with WT 3 h following switch from YPD to YPL medium (*n* = 3 per group). Statistics were calculated using tests described in D. Accompanying plot depicts representative mitochondrial protein GO terms enriched among proteins found to be significantly decreased (fold-change < log_2_(0.75)). **(H)** Western blot depicting levels of Cox2, Pbp1-Flag, and Por1 in cells grown in YPD and after switch to YPL medium. Pbp1Δ500-503 and Pbp1-4A strains had reduced levels of Cox2 and Pbp1-Flag. **(I)** Growth curves of indicated strains cultured in YPL medium. Growth was monitored using the method described in B. Pbp1Δ500-503 and Pbp1-4A strains had slower growth compared with WT. Representative traces from a single experiment are depicted (*n* = 3 per group). Source data are available for this figure: SourceData F3.

We previously reported Pbp1 interacts with the Pumilio RNA-binding protein Puf3 and promotes the expression of its target mRNAs, which encode mitochondrial proteins (van de Poll et al., 2023). Interestingly, Mkt1 has been proposed to regulate Puf3-target mRNA levels, but whether Mkt1 functions like Pbp1 in Puf3-dependent posttranscriptional regulation has not been addressed (Lee et al., 2009). To assess the role of Mkt1 in Puf3-dependent protein expression, we monitored the protein levels of the direct Puf3 target mitochondrial ribosomal protein 51 (Mrp51), the indirect Puf3 target subunit II of cytochrome c oxidase (Cox2), and the non-Puf3 target Por1 (voltage-dependent anion channel porin) in cells grown in YPD and YPL media (Lee and Tu, 2015). Both *mkt1Δ* and *pbp1Δ* cells exhibited reduced Mrp51 and Cox2 protein levels consistent with diminished Puf3-dependent protein expression, while Por1 levels were similar between the WT and KO cells (Fig. 3 A). The *mkt1Δ* and *pbp1Δ* cells also exhibited reduced growth in YPL but not YPD media, indicating the cells have defective respiratory growth (Fig. 3, B and C). Next, we performed a tandem-mass tag/MS (TMT-MS) experiment to comprehensively measure protein abundances in cells grown in both YPD and YPL media. Notably, both *mkt1Δ* and *pbp1Δ* cells exhibited significantly reduced levels of mitochondrial proteins previously shown using multi-omics methods to be encoded by mRNAs directly regulated by Puf3 (Lapointe et al., 2018) (Fig. 3, D–G and Table S4). Collectively, these data indicate Mkt1 functions like Pbp1 to promote Puf3-dependent nuclear-encoded mitochondrial protein expression.

**Figure S3.**
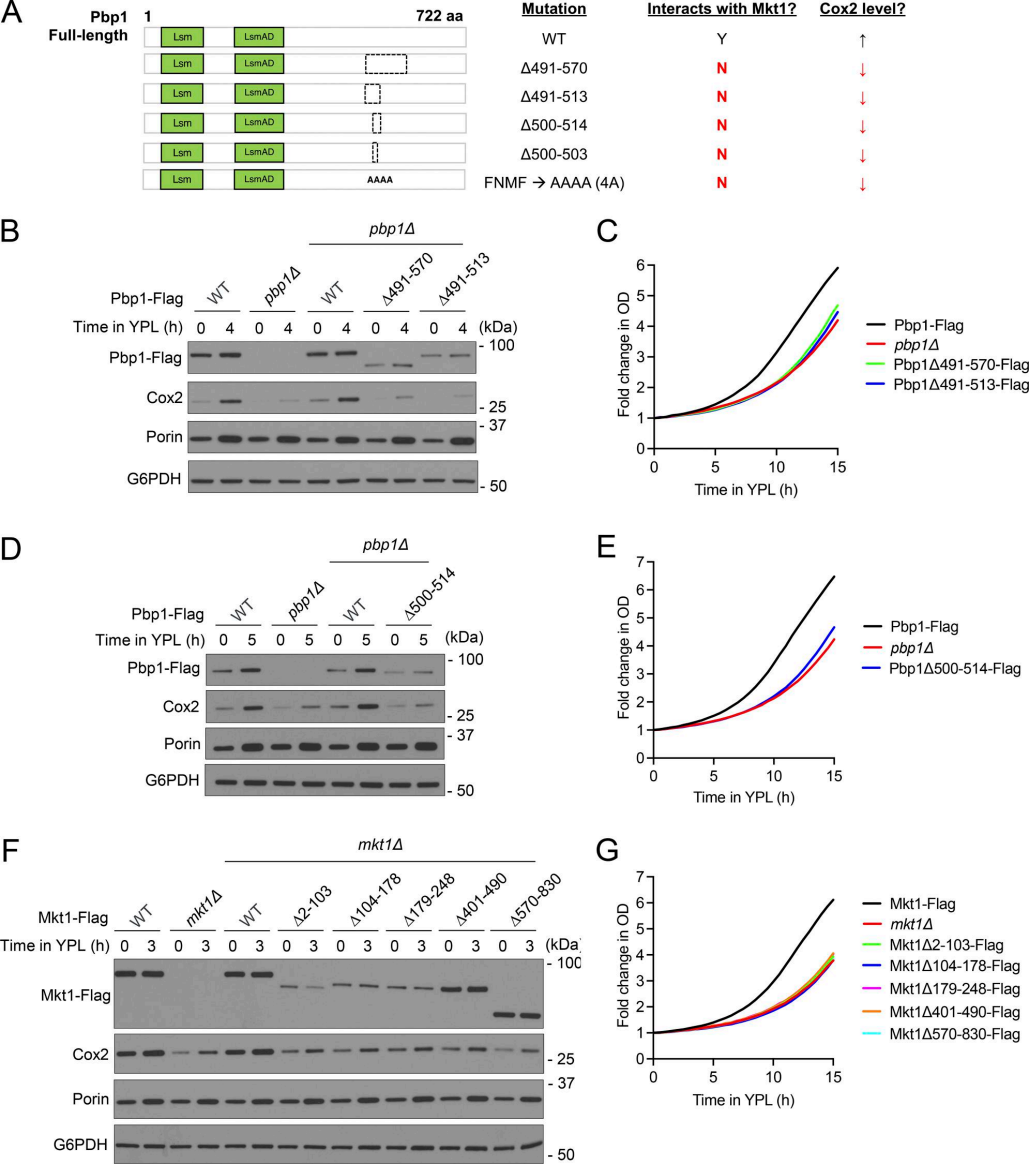
**Puf3-dependent protein expression is disrupted in Mkt1 and Pbp1 truncation strains. (A)** Schematic representation of Flag-tagged Pbp1 truncation strains assayed for their capacity to express mitochondrial proteins and grow in YPL medium. **(B)** Western blot depicting Cox2, Pbp1-Flag, and Por1 protein levels during growth in YPD and following switch to YPL medium. Pbp1Δ491-570 and Pbp1Δ491-513 cells had diminished Cox2 and Pbp1-Flag protein levels compared with WT. **(C)** Growth curves of indicated strains collected using an automatic plate reader during incubation in YPL medium at 30°C. OD_600_ measurements were obtained every 30 min. Note: Pbp1Δ491-570 and Pbp1Δ491-513 cells had reduced growth. Representative traces from a single experiment are depicted (*n* = 3 per group). **(D)** Western blot depicting Cox2, Pbp1-Flag, and Por1 protein levels during growth in YPD and following switch to YPL medium. Pbp1Δ500-514 cells had decreased Cox2 and Pbp1-Flag protein levels compared with WT. **(E)** Growth curves of indicated strains during incubation in YPL medium obtained using the method described in C. Note: Pbp1Δ500-514 cells had reduced growth. Representative traces from a single experiment are depicted (*n* = 3 per group). **(F)** Western blot depicting Cox2, Mkt1-Flag, and Por1 protein levels during growth in YPD and following switch to YPL medium. Mkt1 truncation strains had reduced Cox2 protein abundance compared with WT. **(G)** Growth curves of indicated strains during incubation in YPL medium using method described in C. Note: Mkt1 truncation strains had reduced growth. Representative traces from a single experiment are depicted (*n* = 3 per group). Source data are available for this figure: SourceData FS3.

To further understand how the Mkt1/Pbp1 complex promotes Puf3-dependent protein expression, we assessed Cox2 and Por1 protein levels in Pbp1 and Mkt1 truncation mutant strains. We previously showed cells lacking Pbp1 aa 299–570 and aa 570–830, which encompass Pbp1 low-complexity regions, exhibit reduced Cox2 protein abundance and proposed this was due to defective Pbp1 self-assembly (van de Poll et al., 2023). We found that the Pbp1 truncation strains Pbp1Δ491-570, Pbp1Δ491-513, Pbp1Δ500-514, Pbp1Δ500-503, and Pbp1-4A, which all have disrupted binding to Mkt1, exhibited reduced Cox2 protein levels and growth in YPL medium (Fig. 3, H and I; and Fig. S3, A–E). The levels of mutant Pbp1 were also reduced in these strains, again suggesting Mkt1 binding is important for Pbp1 stability. Finally, the Mkt1 truncation strains, which all have disrupted binding to Pbp1, had reduced Cox2 protein levels and growth in YPL medium (Fig. S3, F and G). Overall, these results indicate an intact Mkt1/Pbp1 complex is required for optimal Puf3-dependent protein expression.

### The Mkt1/Pbp1 complex genetically and physically interacts with Puf3

**Figure 4.**
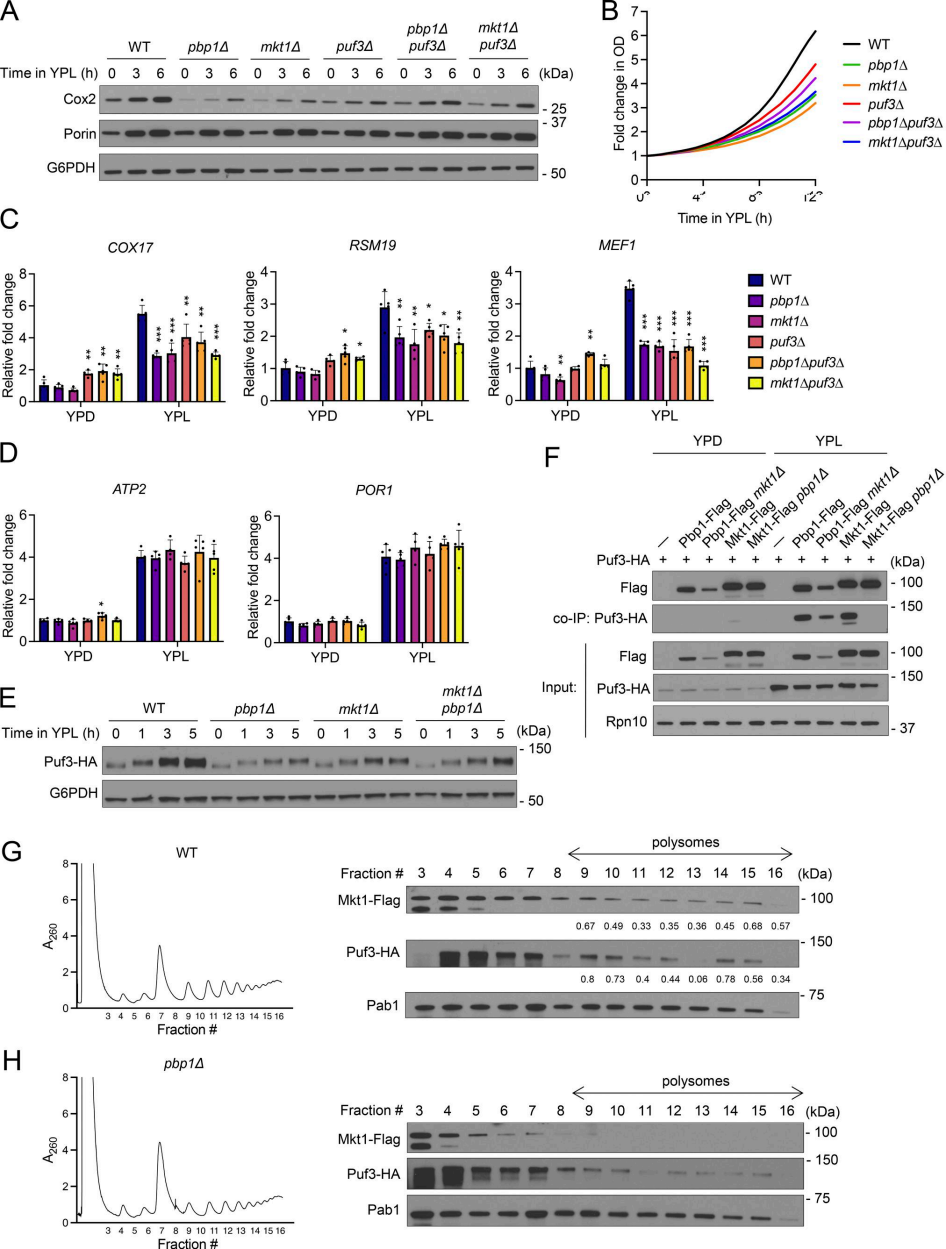
**The Mkt1/Pbp1 complex genetically and physically interacts with Puf3. (A)** Western blot assessing Cox2 and Por1 levels from cells grown in YPD to log phase and following switch to YPL medium. *puf3∆*, *pbp1∆puf3∆*, and *mkt1∆puf3* cells appear to exhibit increased Cox2 protein levels compared with *pbp1∆* and *mkt1∆* cells. **(B)** Growth curves of indicated strains collected using an automated plate reader during incubation in YPL medium. Cells were incubated at 30°C, and measurements were obtained every 30 min. KO strains (*pbp1∆*, *mkt1∆*, *puf3∆*, *pbp1∆puf3∆*, and *mkt1∆puf3*) exhibited reduced growth compared with WT cells (*n* = 4 per group). **(C)** RT-qPCR analysis of Puf3-target mRNA levels from cells collected during log phase growth in YPD and then 3 h after switching to YPL medium. P values were calculated using unpaired, two-sided *t* test (mean ± SD, *n* = 5, except *n* = 4 for *mkt1∆* group). *P < 0.05; **P < 0.01; ***P < 0.001. **(D)** RT-qPCR analysis of non–Puf3-target mRNA levels from cells in C collected during log phase growth in YPD and then 3 h after switching to YPL medium. P values were calculated using unpaired, two-sided *t* test (mean ± SD, *n* = 5, except *n* = 4 for *mkt1∆* group). *P < 0.05. **(E)** Western blot assessing Puf3-HA protein levels from cells grown in YPD to log phase and then 1, 3, and 5 h after switching to YPL medium. Puf3 protein levels were decreased in KO cells, while Puf3 phosphorylation persisted. **(F)** co-IP assessing interactions between Flag-tagged Mkt1 and Pbp1 with Puf3-HA in cells cultured to log phase in YPD and 3 h following switch to YPL medium. Mkt1-Flag and Pbp1-Flag interacted with Puf3-HA preferentially in YPL medium. Note: Mkt1-Flag did not interact with Puf3-HA in *pbp1∆* cells, while Pbp1-Flag and Puf3-HA continued to interact in *mkt1∆* cells despite diminished Pbp1-Flag levels. **(G)** Polysome profile of WT cells collected 3 h following switch from YPD to YPL medium and associated western blot for Mkt1-Flag, Pab1, and Puf3-HA in collected fractions. Numerical values represent relative densities of Mkt1-Flag and Puf3-HA bands compared with corresponding Pab1 bands. Mkt1-Flag and Puf3-HA localized to polysome fractions. **(H)** Polysome profile of *pbp1∆* cells collected 3 h following switch from YPD to YPL medium and associated western blot for Mkt1-Flag, Pab1, and Puf3-HA in collected fractions. Mkt1-Flag was less present in polysome fractions. Source data are available for this figure: SourceData F4.

We previously showed Puf3 genetically interacts with Pbp1 with respect to Cox2 protein levels and Puf3-target mRNA levels (van de Poll et al., 2023). To investigate the genetic relationship between the Mkt1/Pbp1 complex and Puf3, we assessed Cox2 and Por1 levels in single and double KO strains grown in YPD and YPL media. As expected, *mkt1Δ* and *pbp1Δ* cells had a more severe Cox2 deficit compared with *puf3Δ* cells, while *pbp1Δpuf3Δ* and *mkt1Δpuf3Δ* cells exhibited Cox2 levels comparable with *puf3Δ* cells (Fig. 4 A). The defects in Cox2 levels in these strains were associated with reduced growth in YPL medium (Fig. 4 B). Next, we tested the levels of Puf3-target and non-Puf3 target mRNAs in cells grown in YPD and YPL media. Compared with WT cells, Puf3-target mRNA levels were generally decreased in *mkt1Δ* and *pbp1Δ* cells and increased in *puf3Δ*, *pbp1Δpuf3Δ*, and *mkt1Δpuf3Δ* cells during growth in YPD medium (Fig. 4 C). However, only in some cases were these comparisons statistically significant, such as the Cox17 mRNA levels between WT and *puf3Δ*, *pbp1Δpuf3Δ*, and *mkt1Δpuf3Δ* cells. On the other hand, the levels of Puf3-target mRNAs were significantly decreased in all KO cells during growth in YPL medium, indicating both the Mkt1/Pbp1 complex and Puf3 are important, particularly during respiratory growth, for maintaining Puf3-target mRNA levels (Fig. 4 C). The levels of non-Puf3 target mRNAs were similar between the strains during growth in YPD and YPL media (Fig. 4 D). Taken together, these data suggest the Mkt1/Pbp1 complex and Puf3 functionally interact to maintain Puf3-target mRNA levels and promote their translation during respiratory growth.

Puf3 is phosphorylated throughout its N-terminus when cells are switched from glucose to respiratory media to promote the translation of its target mRNAs (Lee and Tu, 2015). We previously found loss of Pbp1 does not impact Puf3 phosphorylation but does diminish Puf3 protein abundance (van de Poll et al., 2023). Cells lacking Mkt1 also exhibited reduced Puf3 levels, while Puf3 phosphorylation remained intact (Fig. 4 E). These data suggest the Mkt1/Pbp1 complex maintains Puf3 protein levels and that reduced Puf3 protein abundance could partially underlie the Puf3-dependent translation defect in *mkt1Δ* and *pbp1Δ* cells.

Pbp1 preferentially interacts with Puf3 during growth in YPL medium (van de Poll et al., 2023). We found Mkt1, like Pbp1, also interacts with Puf3 in a manner that is enhanced during growth in YPL medium, suggesting the interactions between the Mkt1/Pbp1 complex with Puf3 are dependent on metabolic state (Fig. 4 F). Notably, Mkt1 does not interact with Puf3 in *pbp1Δ* cells, while Pbp1 interacts with Puf3 in *mkt1Δ* cells despite reduced Pbp1 protein abundance. These data indicate Mkt1 and Pbp1 associate with Puf3 specifically during respiratory conditions and that Pbp1 is required for Mkt1 to associate with Puf3 and possibly other translation and mRNA processing factors.

**Figure S4.**
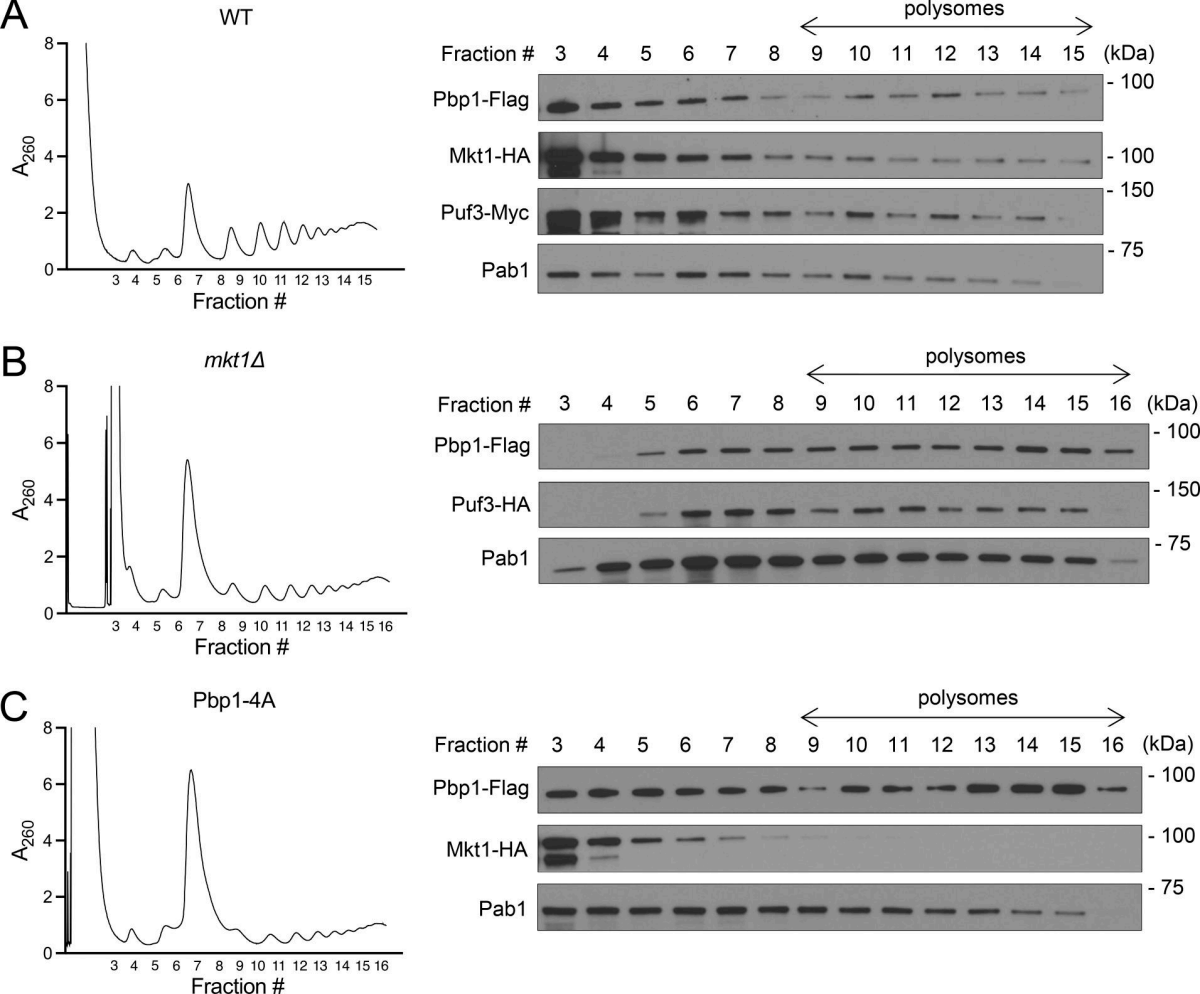
**Assessment of Mkt1/Pbp1 complex and Puf3 localization to polysomes. (A)** Polysome profile of WT cells collected 3 h following switch from YPD to YPL medium and associated western blot for Pbp1-Flag, Mkt1-HA, Puf3-myc, and Pab1 in collected fractions. Note: Mkt1-HA, Pbp1-Flag, and Puf3-myc were present in polysome fractions. **(B)** Polysome profile of *mkt1∆* cells collected 3 h following switch from YPD to YPL medium and associated western blot for Pbp1-Flag, Puf3-HA, and Pab1 in collected fractions. Note: Pbp1-Flag and Puf3-HA were present in polysome fractions. **(C)** Polysome profile of Pbp1-4A cells collected 3 h following switch from YPD to YPL medium and associated western blot for Pbp1-Flag, Mkt1-HA, and Pab1 in collected fractions. Note: Mkt1-HA was less present in polysome fractions. Source data are available for this figure: SourceData FS4.

We have previously shown Puf3 localizes to polysomes specifically under respiratory conditions, while Mkt1 and Pbp1 have also been shown to localize to polysomes (Lee and Tu, 2015; Tadauchi et al., 2004). We speculated the Mkt1/Pbp1 complex and Puf3 interact predominantly during respiratory growth because Puf3 only becomes associated with the Mkt1/Pbp1 complex at polysomes under this condition. To test this hypothesis, we performed polysome profiling and verified Mkt1, Pbp1, and Puf3 are localized to polysomes in cells growing in YPL medium (Fig. 4 G and Fig. S4 A). In addition, as was previously reported, Mkt1 was not detected in polysome fractions in *pbp1Δ* cells, while Pbp1 was localized to polysomes in *mkt1Δ* cells (Fig. 4 H and Fig. S4 B) (Tadauchi et al., 2004). Mkt1 was also not found in polysome fractions in Pbp1-4A cells, which have impaired Mkt1 binding (Fig. S4 C). In sum, these data in combination with our Puf3 co-IP experiment indicate that during respiratory growth Puf3 associates with the Mkt1/Pbp1 complex at polysomes and that either the loss of Pbp1 or disruption of Mkt1-Pbp1 binding renders Mkt1 less able to associate on polysomes with Puf3.

### The Mkt1/Pbp1 complex drives autophagy under respiratory conditions

**Figure 5.**
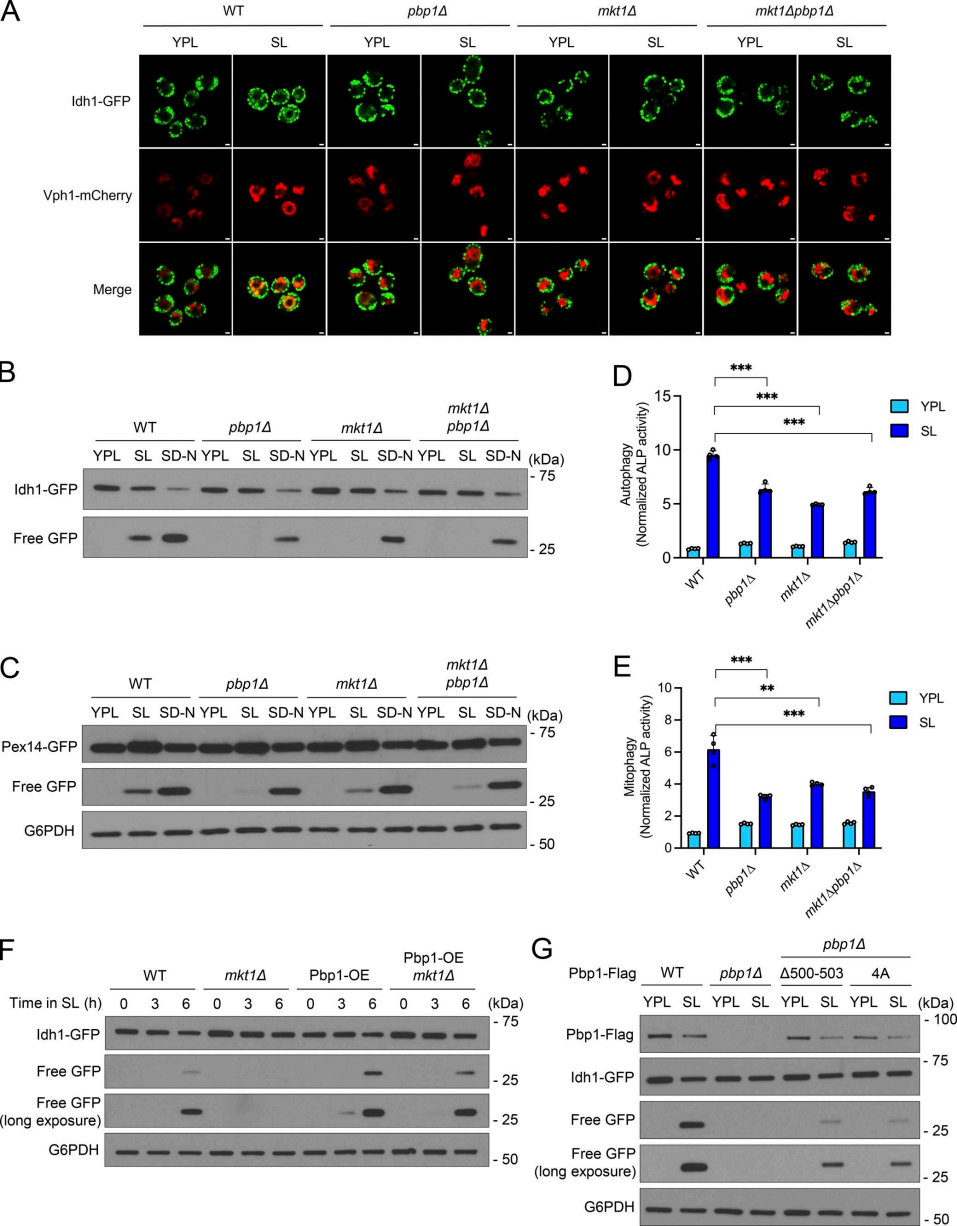
**The Mkt1/Pbp1 complex is required for autophagy during respiratory growth. (A)** Images of WT, *pbp1∆*, *mkt1∆*, and *mkt1∆pbp1∆* cells before and 6 h after switching from YPL to SL medium. Localization of GFP to the vacuole (Vph1-mCherry) indicates mitophagy (Kanki and Klionsky, 2008). *pbp1∆*, *mkt1∆*, and *mkt1∆pbp1∆* cells were unable to induce mitophagy following switch to SL medium. Scale bar = 1 μm. **(B)** GFP cleavage assay showing mitophagy in indicated strains following switch from YPL to either SL or SD-N (high glucose, nitrogen starvation) media for 6 h. The accumulation of free GFP indicates mitophagy. Mitophagy was defective in *pbp1∆*, *mkt1∆*, and *mkt1∆pbp1∆* cells in SL medium. **(C)** GFP cleavage assay showing pexophagy in indicated strains following switch from YPL to either SL or SD-N media for 6 h. Pexophagy was defective in *pbp1∆*, *mkt1∆*, and *mkt1∆pbp1∆* cells in SL medium. **(D)** Alkaline phosphatase assay assessing general autophagy in cells 7 h following switch from YPL to SL medium. *pbp1∆*, *mkt1∆*, and *mkt1∆pbp1∆* cells had reduced alkaline phosphatase activity reflective of a general autophagy defect. P values were calculated using unpaired, two-sided *t* test (mean ± SD; *n* = 4). ***P < 0.001. **(E)** Alkaline phosphatase assay measuring mitophagy in cells 7 h following switch from YPL to SL medium. *pbp1∆*, *mkt1∆*, and *mkt1∆pbp1∆* cells had reduced alkaline phosphatase activity indicative of a mitophagy defect. P values were calculated using unpaired, two-sided *t* test (mean ± SD; *n* = 4). **P < 0.01; ***P < 0.001. **(F)** Pbp1 overexpression rescues mitophagy in *mkt1∆* cells following switch from YPL to SL medium for 6 h. Pbp1 was overexpressed by swapping the *PBP1* promoter for the *TEF1* promoter. Mitophagy was assayed using the Idh1-GFP cleavage assay. **(G)** GFP cleavage assay showing Pbp1Δ500-503 and Pbp1-4A strains had reduced Pbp1 protein levels and disrupted mitophagy following growth in SL medium for 6 h. Note: residual free GFP in Pbp1Δ500-503 and Pbp1-4A cells was detected, which may reflect continued TORC1 repression. Source data are available for this figure: SourceData F5.

We have previously shown that Pbp1 forms intracellular condensates which repress TORC1 to drive autophagy and survival specifically under starvation conditions requiring mitochondrial respiration (Yang et al., 2019). Given our understanding of how Mkt1 binding to Pbp1 promotes mitochondrial protein expression, we hypothesized Mkt1 may also function with Pbp1 in the regulation of autophagy and TORC1 signaling. Using imaging, GFP cleavage, and alkaline phosphatase assays, we found both *mkt1Δ* and *pbp1Δ* cells exhibited impaired autophagy when switched from YPL medium to a minimal (SL) lactate medium (Fig. 5, A–E). On the other hand, *mkt1∆* and *pbp1Δ* cells exhibited robust levels of autophagy when cultured in nitrogen starvation (SD-N) medium, reflecting the specific role of Mkt1 and Pbp1 in regulating autophagy under respiratory conditions (Fig. 5, B and C).

We next performed a GFP cleavage assay with yeast strains that overexpress Pbp1 to understand the functional relationship between Mkt1 and Pbp1 in autophagy. Previously, Pbp1 overexpression was found to inhibit TORC1 signaling in an S288C strain (Takahara and Maeda, 2012). In line with this observation, we observed Pbp1 overexpression led to enhanced autophagy and rescued the autophagy defect in *mkt1Δ* cells (Fig. 5 F). These results suggest Mkt1 is dispensable for Pbp1 to inhibit TORC1 signaling and that Pbp1 is the effector of TORC1 repression in the Mkt1/Pbp1 complex.

**Figure S5.**
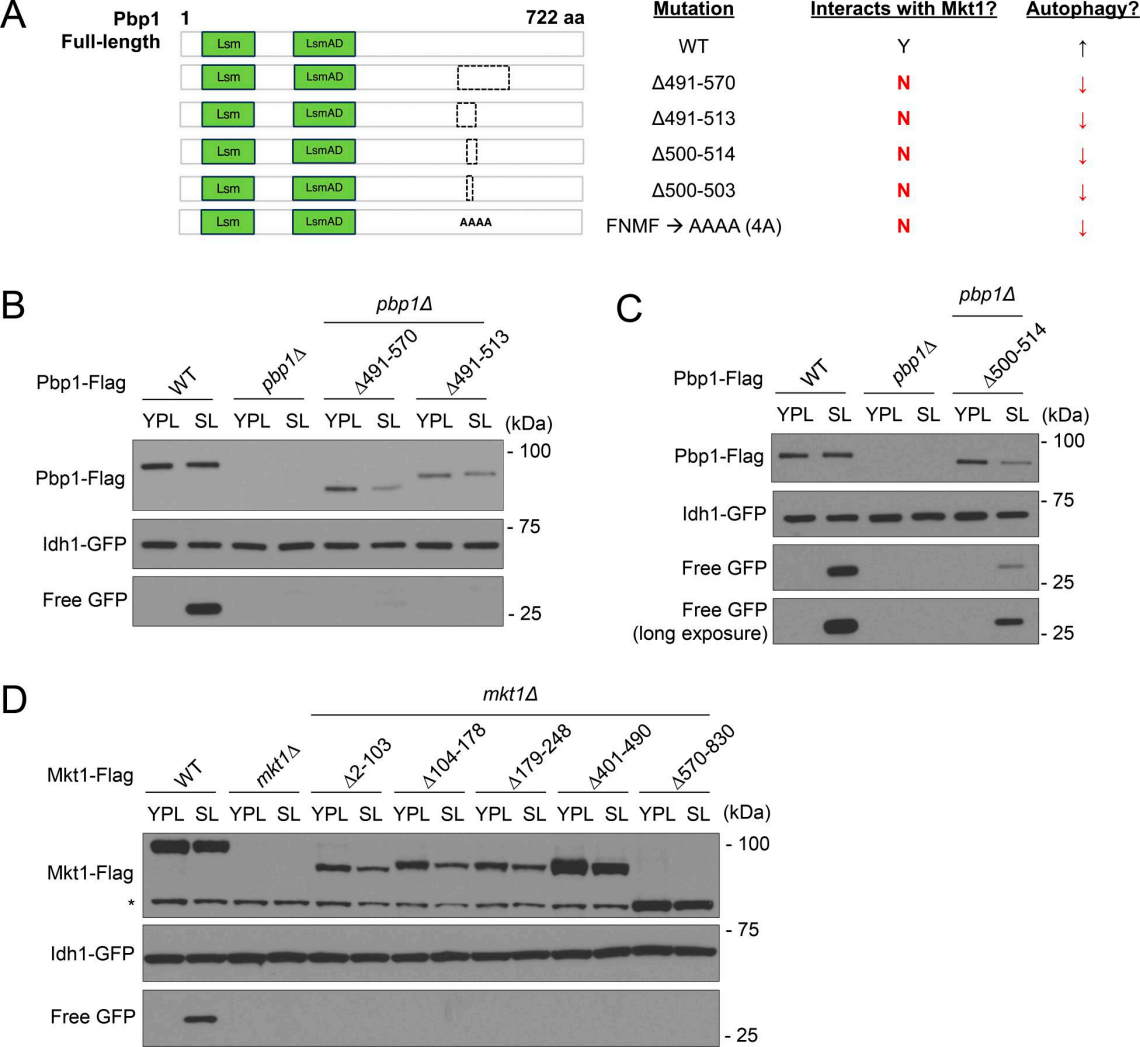
**Autophagy in Mkt1 and Pbp1 truncation strains during growth in minimal respiratory medium. (A)** Schematic representation of Flag-tagged Pbp1 truncation strains tested for their capacity to undergo autophagy during growth in SL medium. **(B)** GFP cleavage assay showing Pbp1Δ491-570 and Pbp1Δ491-513 cells had disrupted mitophagy following incubation in SL medium for 6 h. **(C)** GFP cleavage assay showing Pbp1Δ500-514 cells had disrupted mitophagy following incubation in SL medium for 6 h. Note: residual free GFP in Pbp1Δ500-514 cells was detected, which may reflect continued TORC1 repression. **(D)** GFP cleavage assay showing Mkt1 truncation mutants had disrupted mitophagy following incubation in SL medium for 6 h. * indicates a nonspecific band. Source data are available for this figure: SourceData FS5.

To appreciate the importance of Mkt1-Pbp1 binding to the control of autophagy, we next performed GFP cleavage assays using Mkt1 and Pbp1 truncation strains with defective complex formation. The Pbp1 truncation mutants (Pbp1Δ491-570, Pbp1Δ491-513, Pbp1Δ500-514, Pbp1Δ500-503, and Pbp1-4A) all exhibited autophagy defects (Fig. 5 G and Fig. S5, A–C). Interestingly, we detected residual GFP cleavage in the Pbp1Δ500-514, Pbp1Δ500-503, and Pbp1-4A strains and speculate that this was due to persistent TORC1 signaling repression by Pbp1. In addition, the Mkt1 truncation strains exhibited defective autophagy like *mkt1∆* cells during respiratory growth, presumably due to the markedly reduced levels of Pbp1 protein in these strains (Fig. S5 D). In sum, Pbp1/Mkt1 truncation strains with defective complex formation exhibit compromised autophagy during respiratory growth, indicating an intact Mkt1/Pbp1 complex is required for this process to optimally occur.

### The Mkt1/Pbp1 complex is required for TORC1-dependent repression of autophagy and growth in minimal respiratory medium

**Figure 6.**
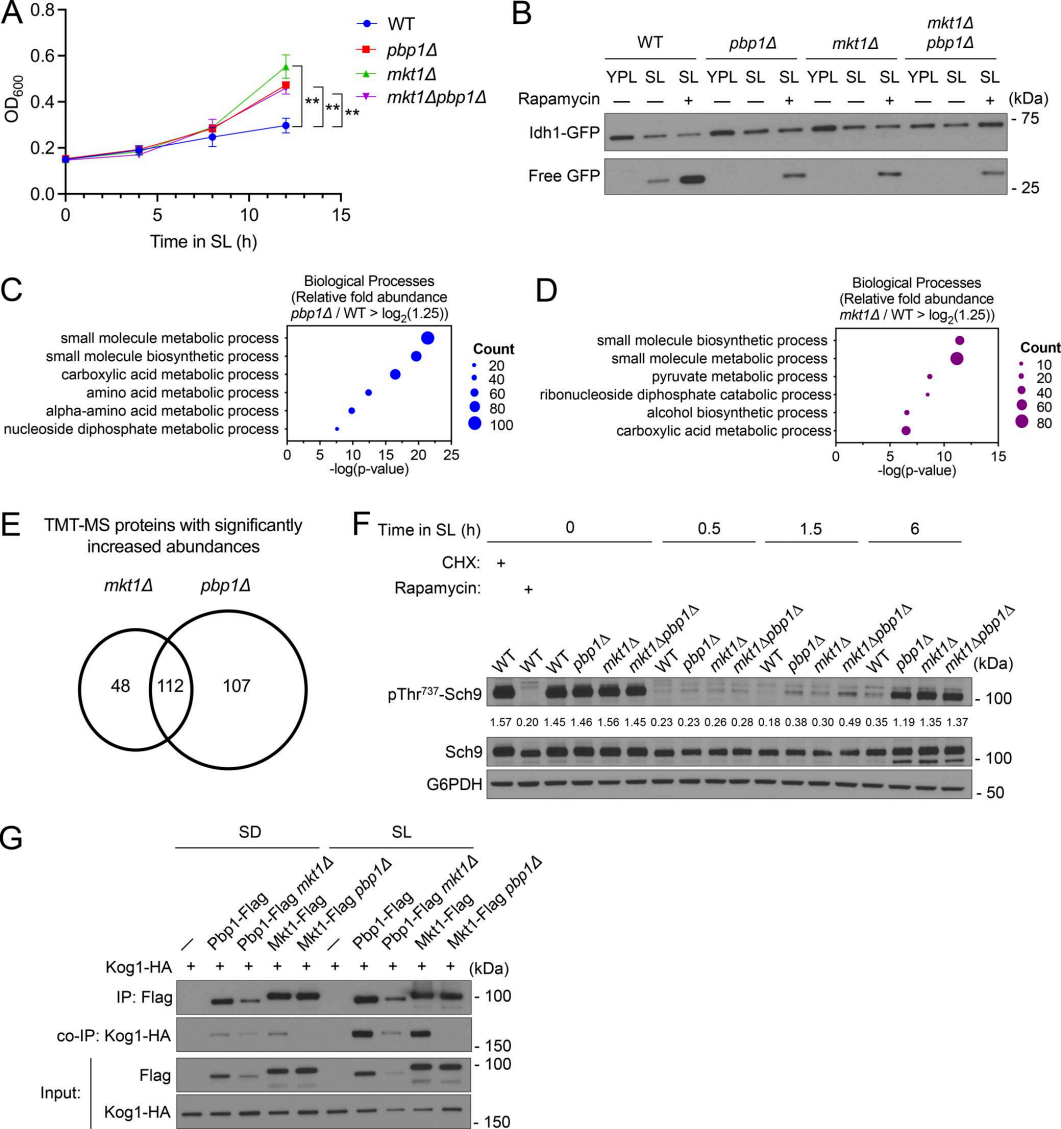
**The Mkt1/Pbp1 complex negatively regulates TORC1 signaling during respiratory growth. (A)** Growth curves of indicated strains grown in batch SL cultures plotted from OD_600_ measurements. *pbp1∆*, *mkt1∆*, and *mkt1∆pbp1∆* cells exhibited increased growth. P values were calculated with unpaired, two-sided *t* test (mean ± SD, *n* = 3). **P < 0.01. **(B)** GFP cleavage assay depicting rescue of autophagy following treatment with 200 nM rapamycin in *pbp1∆*, *mkt1∆*, and *mkt1∆pbp1∆* cells grown in SL medium for 6 h. **(C)** GO term analyses of TMT-MS experiment with *pbp1∆* cells grown for 3 h in SL medium. Protein categories overrepresented among proteins with significantly increased abundances (fold-change > log_2_(1.25)) are depicted. **(D)** GO term analyses of TMT-MS experiment with *mkt1∆* cells grown for 3 h in SL medium. Protein categories overrepresented among proteins with significantly increased abundances (fold-change > log_2_(1.25)) are depicted. **(E)** Venn diagram depicting number of proteins with significantly increased abundances identified in both *mkt1∆* and *pbp1∆* cells in the TMT-MS experiment. **(F)** Western blot depicting the phosphorylation of the TORC1 substrate Sch9 at Thr737 before and following switch from YPL to SL medium. Sch9 phosphorylation was monitored using an antibody specific for phospho-Thr737. All KO cells exhibited robust phospho-Thr737 signal at the 6 h time point. Cells with cycloheximide (25 μg/ml) and rapamycin (200 nM) served as positive and negative controls, respectively. **(G)** co-IP assessing interactions between Flag-tagged Mkt1 and Pbp1 with Kog1-HA 4 h following growth in either SD or SL media. Mkt1-Flag and Pbp1-Flag interacted preferentially in SL medium with Kog1-HA. Mkt1-Flag did not interact with Kog1-HA in *pbp1∆* cells, while Pbp1-Flag interacted with Kog1-HA in *mkt1∆* cells. Source data are available for this figure: SourceData F6.

We next assessed the involvement of the Mkt1/Pbp1 complex in TORC1 signaling repression during respiratory growth. We previously observed *pbp1Δ* cells exhibit a hyperproliferative growth phenotype when cultured in SL medium (Yang et al., 2019). Similarly, *mkt1Δ* cells also exhibited enhanced growth when cultured in SL medium, suggesting Mkt1 may function with Pbp1 to repress TORC1 signaling and induce autophagy (Fig. 6 A). Consistent with this notion, rapamycin treatment was able to rescue autophagy in *mkt1∆* and *pbp1∆* cells following incubation in SL medium (Fig. 6 B). We also performed a TMT-MS experiment and found *mkt1∆* and *pbp1∆* cells grown in SL medium have similar proteomic profiles characterized by increased levels of proteins involved in anabolic metabolism and biosynthesis, consistent with TORC1 hyperactivity (Fig. 6, C–E and Table S5). Next, to monitor TORC1 activity, we assessed phosphorylation of Sch9 at Thr737 by western blot and found that both *mkt1Δ* and *pbp1Δ* cells exhibited markedly increased phosphorylation at this site after 6 h of growth in SL medium (Fig. 6 F). Collectively, these results indicate that the Mkt1/Pbp1 complex is required to repress TORC1 signaling during growth in minimal respiratory medium.

We have shown that Pbp1 interacts with the TORC1 subunit Kog1 preferentially in respiratory conditions (Yang et al., 2019). We next tested the ability of Mkt1 and Pbp1 to interact with Kog1 during growth in minimal glucose (SD) and lactate media. Both Mkt1 and Pbp1 interacted with the TORC1 subunit Kog1 preferentially in SL medium. In addition, as was the case with the Mkt1/Pbp1 complex—Puf3 interaction, the ability of Mkt1 to interact with Kog1 was dependent on Pbp1, but Pbp1 interacted with Kog1 in the absence of Mkt1 (Fig. 6 G). Altogether, these data support the notion that the Mkt1/Pbp1 complex associates with TORC1 during respiratory growth and that the complex is promoting autophagy through repression of TORC1 signaling.

### The Mkt1-G30D point mutation disrupts the abundance and formation of the Mkt1/Pbp1 complex

**Figure 7.**
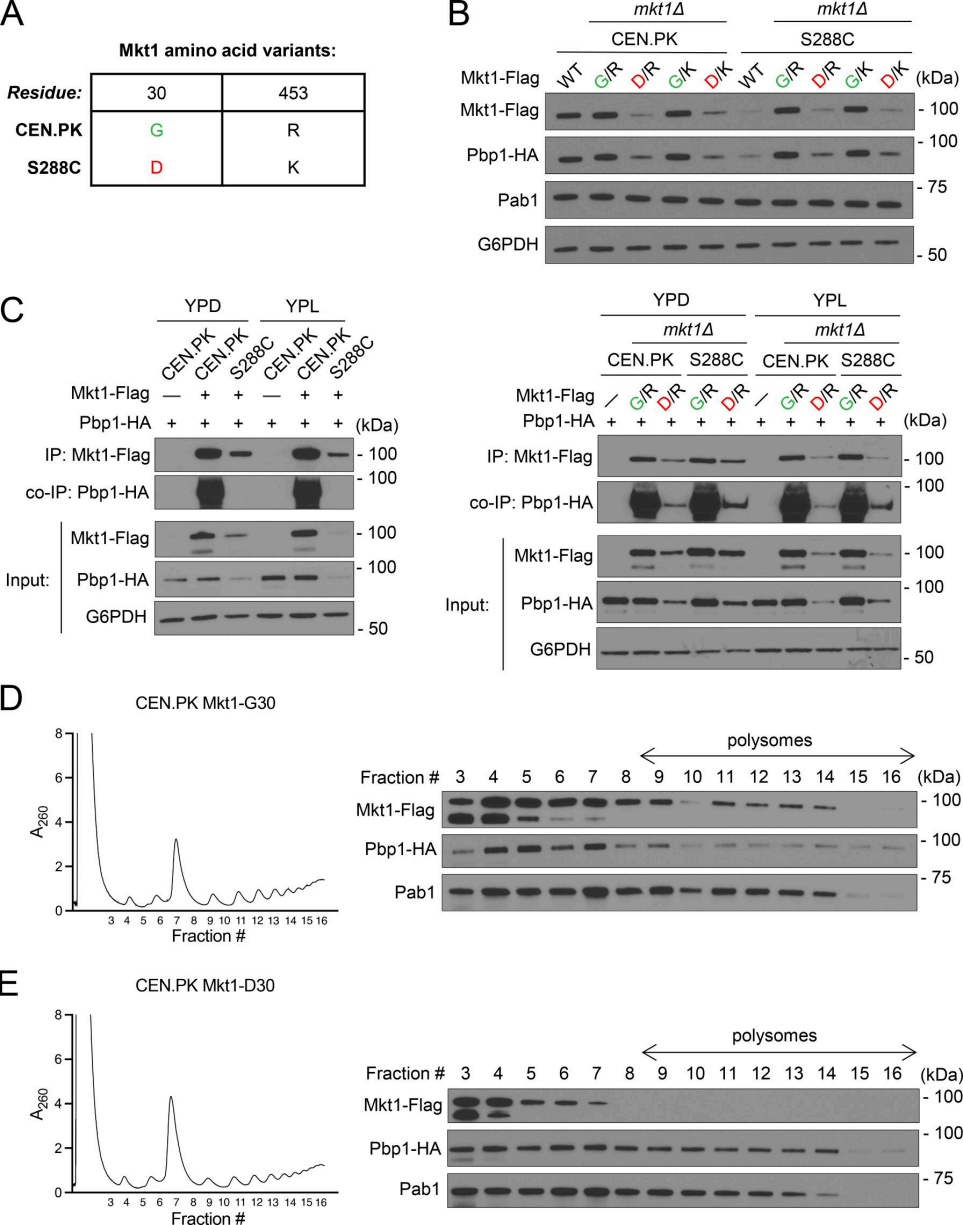
**The Mkt1-G30D point mutation disrupts Mkt1/Pbp1 complex abundance and formation. (A)** Table depicting Mkt1 aa polymorphisms in CEN.PK and S288C strains used in this study. **(B)** Western blot assessing Mkt1-Flag, Pab1, and Pbp1-HA protein levels in CEN.PK and S288C cells collected 3 h following switch from YPD to YPL medium. Note: Mkt1-Flag and Pbp1-HA levels in CEN.PK and S288C strains expressing Mkt1-D30 from the endogenous locus were diminished compared with strains expressing Mkt1-G30. **(C)** co-IP experiments testing interactions between Mkt1-Flag and Pbp1-HA in CEN.PK and S288C cells collected during log phase in YPD and 3 h after switching to YPL medium. Mkt1-Flag interacted poorly with Pbp1-HA in CEN.PK and S288C strains expressing Mkt1-D30 alleles. **(D)** Polysome profile of CEN.PK Mkt1-G30 cells collected 3 h following switch from YPD to YPL medium and associated western blot for Mkt1-Flag and Pbp1-HA in the collected fractions. Mkt1-G30-Flag and Pbp1-HA were detected in polysome fractions. **(E)** Polysome profile of CEN.PK Mkt1-D30 cells collected 3 h following switch from YPD to YPL medium and associated western blot for Mkt1-Flag and Pbp1-HA in the collected fractions. Mkt1-D30-Flag was less present in polysome fractions. Source data are available for this figure: SourceData F7.

The Mkt1-G30D polymorphism has been shown to be a loss-of-function mutation that underlies variation across many yeast complex phenotypes, but the effects of the mutation on Mkt1 are unknown (Table S6). To study the Mkt1-G30D mutation, we decided to perform comparative studies using CEN.PK and S288C yeast strains. Our lab historically has used the CEN.PK strain, which harbors a WT Mkt1-G30 allele, whereas the S288C strain background has the loss-of-function Mkt1-D30 allele (Fig. 7 A). Another polymorphism (R453K) also differentiates the CEN.PK and S288C Mkt1 alleles, and its effects are unknown. We generated CEN.PK and S288C Mkt1 strains expressing alleles derived from substituting aa at the 30th and 453rd positions at their respective endogenous Mkt1 locus (Mkt1-G30/R453, Mkt1-D30/R453, Mkt1-G30/K453, and Mkt1-D30/K453) and used these in our experiments.

When we assessed the abundances of Mkt1 and Pbp1 in our CEN.PK and S288C strains by immunoblot, we noted Mkt1-D30 cells exhibited significantly reduced Mkt1 and Pbp1 levels, suggesting the Mkt1-G30D mutation diminishes complex abundance (Fig. 7 B). Next, we assessed the effects of the Mkt1-G30D point mutation on Mkt1-Pbp1 binding by co-IP and found Mkt1-D30 cells had reduced Mkt1 and Pbp1 protein abundances and diminished binding (Fig. 7 C). These results suggest the Mkt1-G30D mutation disrupts the formation of the Mkt1/Pbp1 complex. To further show this is the case, we performed polysome profiling using CEN.PK Mkt1-G30 and Mkt1-D30 cells and found Mkt1-G30 was present at polysomes while Mkt1-D30 was absent (Fig. 7, D and E). The latter finding in Mkt1-D30 cells mirrors the loss of Mkt1 polysome localization in *pbp1Δ* and Pbp1-4A cells, which have disrupted Mkt1/Pbp1 complex formation (Fig. 4 H and Fig. S4 C). In sum, the Mkt1-G30D point mutation diminishes Mkt1/Pbp1 complex abundance and formation.

### The Mkt1-G30D polymorphism underlies the capacity of yeast to promote Puf3-dependent mitochondrial protein expression

**Figure 8.**
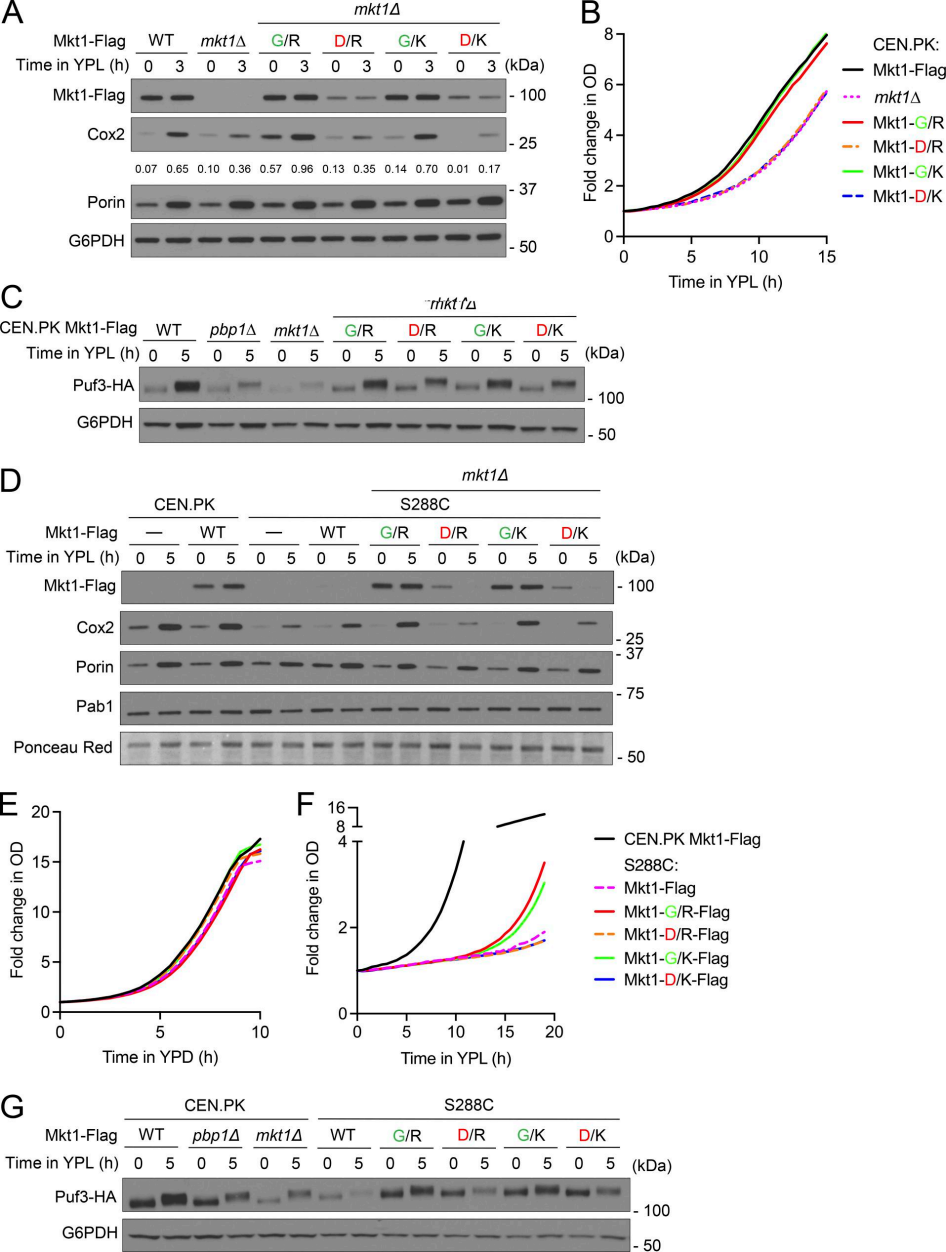
**The Mkt1-G30D point mutation decreases Puf3-dependent protein expression. (A)** Western blot reflecting Cox2, Mkt1-Flag, and Por1 protein levels in CEN.PK cells during growth in YPD and 3 h after switch to YPL medium. Numerical values represent relative densities of Cox2 bands compared with corresponding G6PDH bands. Note: Endogenous substitution of Mkt1-D30 alleles diminished Mkt1-Flag and Cox2 levels. **(B)** Growth curves of indicated strains monitored in an automated plate reader during incubation at 30°C in YPL medium. OD_600_ measurements were taken every 30 min. CEN.PK Mkt1-D30 cells exhibited reduced growth compared with WT and Mkt1-G30 cells. Traces are from one representative experiment (*n* = 3 per group). **(C)** Western blot depicting Puf3-HA levels in CEN.PK cells grown in YPD to log phase and 5 h following switch to YPL medium. CEN.PK Mkt1-D30 cells had reduced Puf3-HA protein accumulation in YPL medium compared with WT and Mkt1-G30 cells. **(D)** Western blot depicting Cox2, Mkt1-Flag, Pab1, and Por1 protein levels from CEN.PK and S288C cells grown to log-phase in YPD and 5 h following switch to YPL medium. Endogenous substitution of Mkt1-G30 alleles rescued Cox2 and Mkt1-Flag protein levels in the S288C strain background. **(E)** Growth curves of CEN.PK and S288C cells during incubation in YPD medium obtained using the method described in B. All strains grew at similar rates (*n* = 3 per group). **(F)** Growth curves of CEN.PK and S288C cells during incubation in YPL medium obtained using the same method described in B. Endogenous substitution of Mkt1-G30 alleles in S288C yeast enhanced growth in YPL medium (*n* = 3 per group). **(G)** Western blot depicting Puf3-HA levels in CEN.PK and S288C cells grown in YPD and 5 h following switch to YPL medium. Puf3-HA levels in cells collected from YPL medium were reduced in S288C WT and Mkt1-D30 cells and rescued in S288C Mkt1-G30 cells. Source data are available for this figure: SourceData F8.

We next determined the effect of the Mkt1-G30D point mutation on Puf3-dependent mitochondrial protein expression in CEN.PK yeast. We found cells expressing Mkt1-D30 alleles exhibited reduced Cox2 and Mkt1 protein levels and reduced growth in YPL medium like *mkt1Δ* cells (Fig. 8, A and B). In addition, CEN.PK Mkt1-D30 cells had reduced Puf3 protein levels compared with WT and Mkt1-G30 cells (Fig. 8 C). These results indicate Mkt1-D30 cells phenocopy the Puf3-dependent protein expression and respiratory growth defects of *mkt1Δ* cells.

Next, we reasoned that a yeast strain that naturally harbors the Mkt1-D30 allele, such as the S288C strain, may have an underlying defect in Puf3-dependent protein expression that can potentially be rescued by substituting it for an Mkt1-G30 allele. To test this hypothesis, we monitored Cox2 and Por1 protein levels in CEN.PK and S288C WT, Mkt1-G30, and Mkt1-D30 strains. We found S288C cells have a marked Cox2 protein deficit suggestive of an underlying defect in Puf3-dependent protein expression (Fig. 8 D). In addition, we observed Cox2 protein levels were rescued in S288C Mkt1-G30 cells compared with S288C WT and Mkt1-D30 cells. We also observed CEN.PK and S288C cells grew similarly when incubated in YPD medium, but S288C yeast grew poorly in YPL medium compared with CEN.PK yeast (Fig. 8, E and F). Despite this defect, the introduction of the Mkt1-G30 alleles was able to partially rescue the respiratory growth of S288C yeast (Fig. 8 F). In addition, we observed Puf3 protein abundance was diminished in S288C WT and Mkt1-D30 cells and was rescued in S288C Mkt1-G30 cells (Fig. 8 G). Together, these results indicate the Mkt1-G30D polymorphism underlies the capacity of yeast to promote Puf3-dependent expression of mitochondrial proteins that are important for respiratory growth.

### The Mkt1-G30D polymorphism underlies the capacity for yeast to undergo autophagy in respiratory conditions

**Figure 9.**
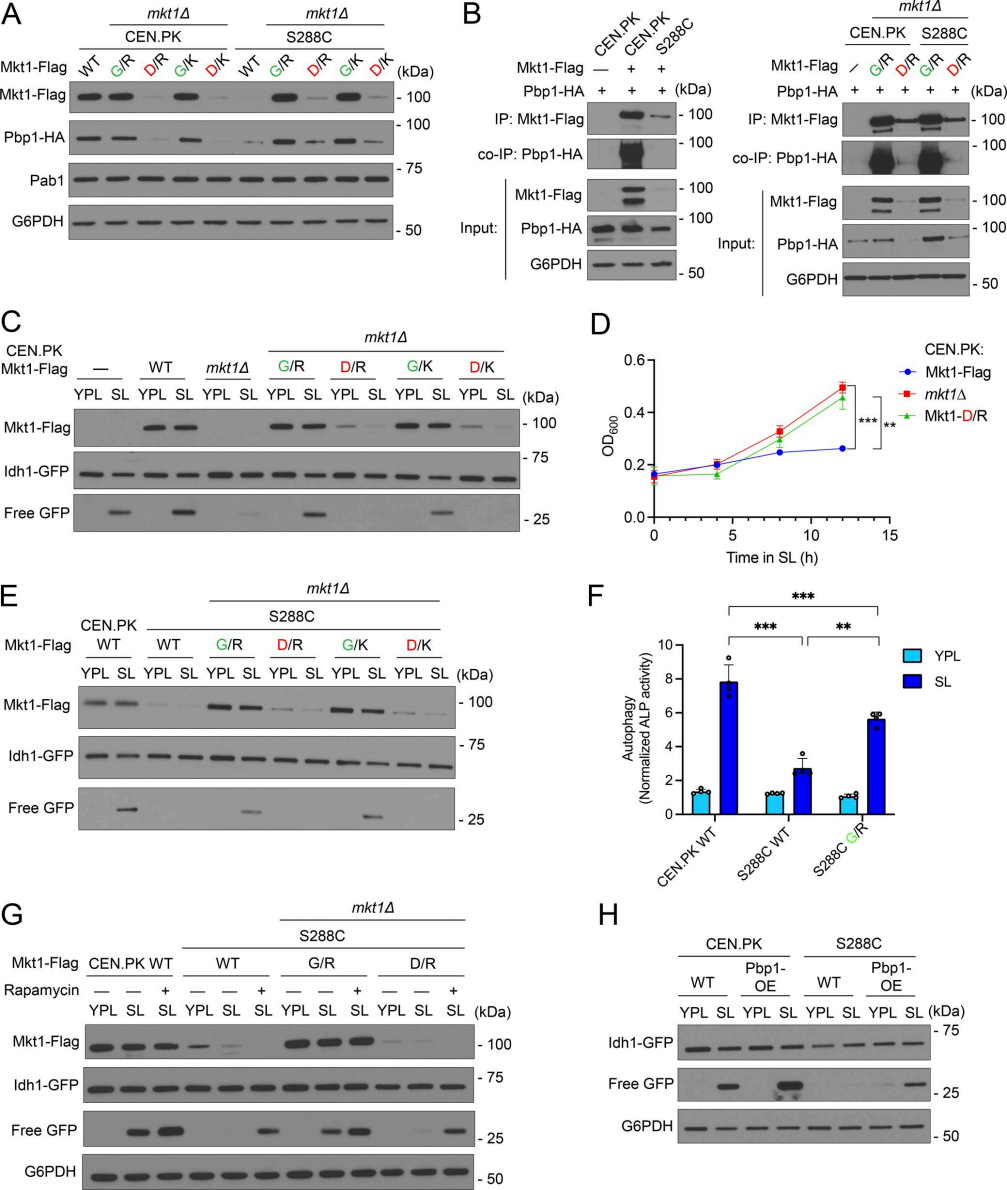
**The Mkt1-G30D polymorphism underlies the capacity of yeast to undergo autophagy during respiratory growth. (A)** Western blot showing CEN.PK and S288C cells expressing endogenously substituted Mkt1-D30 alleles had reduced Mkt1-Flag and Pbp1-HA protein levels 3 h following switch from YPL to SL medium. **(B)** co-IP experiments testing interactions between Mkt1-Flag and Pbp1-HA in CEN.PK and S288C cells 3 h following switch from YPL to SL medium. Mkt1-Flag interacted poorly with Pbp1-HA in CEN.PK and S288C cells expressing Mkt1-D30 alleles. **(C)** GFP cleavage assay depicting impaired mitophagy and reduced Mkt1 protein levels in CEN.PK Mkt1-D30 cells 6 h following switch from YPL to SL medium. **(D)** Growth curves of CEN.PK strains incubated in batch SL cultures plotted from OD_600_ measurements. CEN.PK Mkt1-D30 cells phenocopied hyperproliferative growth of *mkt1∆* cells. P values were calculated using unpaired, two-sided t test (mean ± SD, *n* = 4). ***P < 0.001. **(E)** GFP cleavage assay reflecting endogenous substitution of Mkt1-G30 alleles rescued mitophagy and Mkt1-Flag protein levels in S288C yeast. Cells were collected before and 6 h following switch from YPL to SL medium. **(F)** Alkaline phosphatase assay to monitor general autophagy 6 h following growth in SL medium. S288C WT cells had decreased general autophagy, which was rescued by an endogenously substituted Mkt1-G30 allele. P values were calculated using unpaired, two-sided *t* test (mean ± SD, *n* = 4). **P < 0.01; ***P < 0.001. **(G)** GFP cleavage assay representing partially rescued mitophagy in S288C WT and Mkt1-D30 cells treated with rapamycin following 6 h in SL medium. **(H)** GFP cleavage assay showing Pbp1 overexpression partially rescued mitophagy defect in S288C WT cells grown in SL medium for 6 h. Source data are available for this figure: SourceData F9.

Given the effects of the Mkt1-G30D mutation on Mkt1/Pbp1 complex abundance and formation, Puf3-dependent protein expression, and its reported links to TORC1 signaling variation, we next decided to test the effects of the mutation on autophagy during respiratory growth (Gagneur et al., 2013; Weith et al., 2023). We observed during growth in SL medium that CEN.PK and S288C Mkt1-D30 cells had reduced Mkt1/Pbp1 complex abundance and highly disrupted Mkt1-Pbp1 binding (Fig. 9, A and B). CEN.PK Mkt1-D30 cells also had defective Idh1-GFP cleavage and increased growth in SL medium (Fig. 9, C and D). In addition, in our first report describing yeast autophagy during growth in medium containing non-fermentable carbon sources, we observed CEN.PK and W303, but not S288C yeast, could undergo autophagy (Wu and Tu, 2011). At the time, we hypothesized the S288C autophagy defect might be due to the presence of SNPs that are absent in the autophagy-competent CEN.PK and W303 yeast strains. Since we have shown CEN.PK Mkt1-D30 cells have defective autophagy during growth in SL medium, we hypothesized the Mkt1-G30D mutation in S288C yeast may underlie the autophagy defect in this strain and could be corrected by substitution with the Mkt1-G30 alleles. Remarkably, expression of the Mkt1-G30 alleles in S288C yeast rescued autophagy during growth in SL medium, presumably due to a restoration of Pbp1 protein abundance and TORC1 signaling repression (Fig. 9, E and F). In support of this notion, treatment with rapamycin partially rescued autophagy in S288C WT and Mkt1-D30 cells (Fig. 9 G). Furthermore, we found we were able to rescue autophagy in S288C WT yeast by overexpressing Pbp1, consistent with the previous finding that Pbp1 overexpression represses TORC1 signaling in S288C yeast and a role for Pbp1 as the effector of TORC1 signaling repression in the Mkt1/Pbp1 complex (Takahara and Maeda, 2012) (Fig. 9 H). Overall, these results indicate that the Mkt1-G30D polymorphism largely underlies the capacity for yeast to undergo autophagy during nutritional stress under respiratory conditions.

## Discussion

In this study, we show Mkt1 exists in a complex with Pbp1 that functions to promote both the expression of Puf3-target nuclear-encoded mitochondrial proteins during growth in a replete respiratory (YPL) medium and the repression of TORC1 signaling to drive autophagy during growth in a minimal respiratory (SL) medium. We also show for the first time that the Mkt1-G30D loss-of-function mutation present in laboratory yeast decreases the abundance of the Mkt1/Pbp1 complex and disrupts both Puf3-dependent protein expression and autophagy during respiratory growth, helping explain previous genetics studies that identified the Mkt1-G30D polymorphism as a QTL underlying variation in mitochondrial function-related phenotypes. Collectively, these results point to a fundamental role for the Mkt1/Pbp1 complex in promoting adaptive processes to respiratory growth and help explain why Mkt1 is a mutational hotspot for evolutionary adaptation to changes in fermentable carbon source availability (Anderson et al., 2010).

Mkt1 was first proposed to be a Pbp1-binding partner over 20 years ago, but to date little is known regarding their interaction (Tadauchi et al., 2004). Using IP assays guided by AlphaFold predictions, we identified a previously uncharacterized region of Pbp1 (aa 500–503) that is required for Mkt1/Pbp1 binding. Based on our complex structure predictions, we proposed Pbp1 aa 491–549 encompasses an MBR throughout which Mkt1 possibly contacts Pbp1. We speculate this region contains short linear motifs composed of helical structures that mediate binding to Mkt1 analogous to other mRNA processing proteins (Jonas and Izaurralde, 2013). The MBR aa sequence is only conserved in yeast, but we speculate Pbp1/ataxin-2 homologs in other organisms contain similar protein regions which mediate binding to Mkt1 and other proteins.

To identify Mkt1-like proteins in higher organisms, we used Foldseek and found Mkt1 is predicted to bear structural homology to ASTE1 and FAM120 proteins. We tentatively propose that Mkt1 is conserved in higher organisms in the forms of these proteins and that Mkt1 and these Mkt1-like proteins represent a previously undefined protein group within the Rad2/XPG protein family. FAM120A has been shown to be a mammalian stress granule protein and has been reported to interact with miRNA proteins to control miRNA fate, while ASTE1 and FAM120B have been reported to exhibit nuclease activity and function in DNA repair (Jain et al., 2016; Kelly et al., 2019; Andrews et al., 2018; Zhao et al., 2021). These studies point to a broad range of functions performed by this poorly understood group of proteins and suggest other possible functions for Mkt1 in yeast and other organisms.

We recently reported Pbp1 is required for optimal translation of Puf3-target mRNAs (van de Poll et al., 2023). In our current study, we found that loss of Mkt1 as well as genetic disruption of the Mkt1-Pbp1 complex compromised Puf3-dependent protein expression. How the Mkt1-Pbp1 complex helps promote the translation of Puf3-target mRNAs remains unclear but presumably involves factors thought to bind Mkt1 and Pbp1. For instance, the Mkt1/Pbp1 complex may act in a manner dependent on Pab1, which has been reported to bind Pbp1 and be involved in Pumilio protein–dependent translational repression and mRNA deadenylation (Mangus et al., 1998; Lee et al., 2010; Chritton and Wickens, 2010). In addition, Pbp1 interacts with the DEAD-box helicase Dhh1, which has been reported to induce translational repression, mediate 5′-3′ mRNA decay, and to function in Pumilio protein–mediated control of mRNA fate along with the CCR4–Not complex (Goldstrohm et al., 2006; Nonhoff et al., 2007; Swisher and Parker, 2010; Carroll et al., 2011). Mkt1 has also been reported in trypanosomes to interact with Pumilio proteins and other factors implicated in Pumilio protein–dependent posttranscriptional regulation, and in yeast has been shown to localize to P-bodies and stress granules, suggesting it may interact with posttranscriptional regulators in the control of Puf3-target mRNA fate (Singh et al., 2014; Lewis et al., 2014; Jain et al., 2016). In sum, we speculate that the Mkt1/Pbp1 complex may promote the translation of Puf3-target mRNAs by opposing translational repression and/or mRNA decay by factors previously implicated in Pumilio protein–dependent posttranscriptional regulation.

We have previously shown Pbp1 forms condensates specifically during respiratory growth which function to repress TORC1 signaling and drive autophagy under conditions that require mitochondrial respiration (Yang et al., 2019). In this study, we report that loss of Mkt1 also causes defective autophagy during respiratory growth. Based on these data, we propose that this is due to reduced protein levels of Pbp1, which we hypothesize acts as the primary repressor of TORC1 signaling in the complex. This hypothesis is supported by our findings showing Pbp1 truncation strains lacking Mkt1 binding exhibit residual autophagy compared with *pbp1Δ* cells, Pbp1 overexpression can rescue autophagy in *mkt1Δ* cells, and a previous study reporting Pbp1 overexpression represses TORC1 signaling in S288C yeast (Takahara and Maeda, 2012). In the future, identifying the TORC1 signaling events that are regulated by the Mkt1/Pbp1 complex will lead to a more nuanced understanding of how yeast autophagy is regulated during respiratory growth.

Mkt1 was first reported by Reed Wickner, with the observation that laboratory strains in his collection exhibited temperature-sensitive defects in the maintenance of the dsRNA encoding the viral killer toxin K2 (Wickner, 1980; Wickner, 1987). Since the identification of the Mkt1-G30D polymorphism in the early 2000s, it has been shown in laboratory yeast to underlie variation in many complex phenotypes (Table S6), but surprisingly the effects of the mutation on the Mkt1 protein had not been studied. We show for the first time using CEN.PK and S288C yeast that the Mkt-G30D point mutation causes a marked reduction in the abundance and formation of the Mkt1/Pbp1 complex, providing an explanation for its loss-of-function effects. Functionally, CEN.PK Mkt1-D30 strains phenocopied *mkt1Δ* and *pbp1Δ* cells with respect to loss of Puf3-dependent mitochondrial protein expression and autophagy. Strikingly, WT S288C yeast exhibited the same defects seen in CEN.PK Mkt1-D30 cells and were largely rescued by endogenous expression of the CEN.PK Mkt1-G30 allele. Therefore, it appears that the Mkt1-G30D point mutation significantly underlies the differences in Puf3-dependent protein expression and autophagy in our strains. We hypothesize that the effects of the Mkt1-G30D polymorphism on the Mkt1/Pbp1 complex reported here also explain the mutation’s effects on other traits, which have previously been linked to it.

This work underscores the importance of strain selection for the interpretation and reproducibility of previously published work in yeast biology. We speculate that yeast strains harboring the loss-of-function Mkt1-D30 allele, such as the S288C strain, may not be ideal for studying processes that are impacted by the Mkt1/Pbp1 complex. For example, Puf3 has been proposed to function primarily to drive the degradation of its target mRNAs, but we and others have also shown Puf3 undergoes phosphorylation during growth in non-fermentable medium that switches its function to promoting their translation (Olivas and Parker, 2000; Lee and Tu, 2015; Liu et al., 2021b). Given that many yeast studies on Puf3 have utilized strains containing the Mkt1-G30D mutation, we suspect that inconsistencies in reported Puf3 function can be explained by whether the strain used harbored this mutation or not. In addition, we previously reported S288C yeast exhibited an autophagy defect when cultured in minimal respiratory medium and speculated at the time that this was due to the presence of polymorphisms in S288C yeast (Wu and Tu, 2011). Gratifyingly, we have found that autophagy in S288C yeast under this condition was largely rescued by Mkt1-G30 alleles, a result which reconciled our previous work and highlighted the impact of the Mkt1-G30D mutation on autophagy and growth signaling (Gagneur et al., 2013; Weith et al., 2023). Furthermore, given the highly penetrant and pleiotropic effects of the Mkt1-G30D mutation, we speculate that the usage of S288C yeast and other strains that harbor Mkt1-D30 alleles may have caused discrepancies between yeast studies on processes known to be impacted by the Mkt1-G30D polymorphism. Because of this, we caution against the use of Mkt1-D30 strains to study biology impacted by the Mkt1-G30D polymorphism and advocate for a comparative approach in which more than one strain is used to study processes with complex genetic underpinnings.

Finally, Pbp1 is the yeast homolog of ataxin-2, a well-known genetic modifier of amyotrophic lateral sclerosis and spinocerebellar ataxia in humans (Pulst et al., 1996; Sanpei et al., 1996; Elden et al., 2010). Ataxin-2 is thought to form complexes with proteins involved in RNA processing and translation that are conserved from yeast to humans such as the poly(A)-binding protein, the Lsm protein LSM12, and the mRNA decay factor DDX6 (Satterfield and Pallanck, 2006; Nonhoff et al., 2007; Ayache et al., 2015). Based on this work, we speculate ataxin-2 complexes with these and other proteins, including possibly FAM120 proteins, may regulate processes not limited to posttranscriptional regulation but also mTORC1 signaling, mitochondrial biogenesis, and stress responses. Future work is needed to further establish the biochemical, functional, and structural characteristics of ataxin-2 complexes in yeast and higher organisms to improve our understanding of their roles in biology and disease.

## Materials and methods

### Yeast strains

All strains were made using prototrophic CEN.PK or *HAP1*^*+*^ S288C yeast strains and are listed in Table S7 (van Dijken et al., 2000; Hickman and Winston, 2007). Gene deletions and C-terminal tagging were performed by gene targeting of PCR-based transformation cassettes (Longtine et al., 1998). Mkt1 and Pbp1 deletion and point mutation alleles were constructed using the Q5 Site-Directed Mutagenesis Kit (New England Biolabs) and then integrated into the endogenous locus in *mkt1∆* or *pbp1∆* strains. Overexpression strains were constructed by swapping the endogenous *PBP1* promoter for the *TEF1* promoter.

### Media used in this study

Liquid culture media used were YPD (1% yeast extract [Bio Basic], 2% peptone [BD Biosciences], and 2% glucose), YPL (1% yeast extract, 2% peptone, and 2% lactate [Sigma-Aldrich]), SL (0.67% yeast nitrogen base without aa [BD Biosciences] and 2% lactate), SD (0.67% yeast nitrogen base without aa and 2% glucose), SCL (0.79% complete supplement mixture [Sunrise Science Products] and 2% lactate), and SD-N (0.17% yeast nitrogen base without aa and ammonium sulfate [BD Biosciences] and 2% glucose).

### Media switches

For time-course experiments with YPD and YPL media, cells were incubated in 1 ml of YPD medium overnight, then diluted to OD_600_ = 0.2 in fresh YPD and grown in log phase for 3–4 h. Samples were collected when indicated, then cells were spun down, washed, and resuspended to OD_600_ = 0.7 in YPL medium, and samples were collected at the indicated time points.

For time-course experiments with SD, SD-N, and SL media, cells were incubated overnight in 1 ml of YPD cultures, then diluted to OD_600_ = 0.2 in 5 ml of fresh YPD and grown for at least two generations. Cells were spun down, washed, and resuspended in YPL medium at a low OD and incubated overnight to mid-log phase. Samples were collected when indicated, then cells were spun down, washed, and resuspended to OD_600_ = 0.7 in either SD, SD-N, or SL media, and samples were collected at the indicated time points.

### Growth assays

Growth assays in YPD and YPL media were performed in clear-bottom 96-well plates with the SPARK multimode plate reader (Tecan). Overnight YPD cultures were diluted into 5 ml of fresh YPD cultures and grown to log phase for 3–4 h. Cells were then diluted to OD_600_ = 0.1 in fresh YPD or YPL media, and 100 μl of each culture was pipetted into indicated number of wells. Plates were incubated at 30°C, and OD_600_ measurements were obtained every 30 min. For growth assays in SL medium, cells were prepared in the same manner described in the Media switches method, diluted into flasks at an OD_600_ = 0.15, and OD_600_ measurements were obtained at the indicated time points. Each growth assay figure depicts results from one representative experiment performed at least twice.

### TCA-precipitated protein lysate preparation

Cell pellets (5 ODs) were quenched in 20% TCA for at least 15 min on ice, pelleted, and frozen at −80°C until further use. Pellets were washed with ice-cold 100% acetone, air-dried, and resuspended in 250 μl of urea extraction buffer (6 M urea, 50 mM Tris-HCl, pH 7.5, 1 mM PMSF, 1% SDS, 5 mM EDTA, 1 mM DTT, 5 µM pepstatin A, 10 µM leupeptin, and 2× protease inhibitor cocktail [Roche]) and lysed by five rounds of bead beating (30 s of bead-beating/2 min of cooling on ice). Lysates were then incubated for 5 min at 65°C, centrifuged at maximum speed for 3 min, vortexed briefly, and then centrifuged again at maximum speed for 1 min. The supernatants were collected, and protein concentrations were normalized using a bicinchoninic acid assay kit (Thermo Fisher Scientific).

### Western blot

Protein lysate samples were run on NuPAGE 4–12% Bis-Tris gels (Thermo Fisher Scientific), except Puf3-HA samples, which were run on 3–8% Tris-acetate gels (Thermo Fisher Scientific). Wet transfers were performed at 350 mA for 80 min onto 0.45-µm nitrocellulose membranes. Membranes were stained with Ponceau Red to verify protein loading and transfer. Membranes were blocked with 5% milk/TBST and incubated in target primary antibodies at room temperature for 1–2 h or overnight at 4°C. Membranes were then washed three times in TBST and incubated in HRP-conjugated secondary antibodies at room temperature for 1–2 h or overnight at 4°C. Following incubation, membranes were washed three times in TBST before visualizing with either Pierce ECL Western Blotting Substrate or SuperSignal West Pico PLUS Chemiluminescent Substrate (Thermo Fisher Scientific) using X-ray film. The following antibodies were used in this study: mouse monoclonal anti-Flag (Cat# F1804; Sigma-Aldrich, RRID:AB_262044), rabbit anti-FLAG M2 antibody (Cat# 2368; Cell Signaling Technology, RRID:AB_2217020), mouse monoclonal anti-Pab1 antibody (Cat# MCA-1G1; EnCor Biotechnology, RRID:AB_2572370), mouse anti-Cox2 antibody (Cat# 459150; Thermo Fisher Scientific, AB_2532228), mouse anti-porin antibody (Cat# 459500; Thermo Fisher Scientific, RRID:AB_2532239), rabbit anti-G6PD antibody (Cat# A9521; Sigma-Aldrich, RRID:AB_258454), anti-GFP from mouse IgG1κ (clones 7.1 and 13.1) (Cat# 11814460001; Roche, RRID:AB_390913), rabbit anti-HA antibody (Cat# 3724; Cell Signaling Technology, RRID:AB_1549585), and rabbit anti-Rpn10 (Cat# ab98843; Abcam, RRID:AB_10672806). Densitometry quantification was performed using ImageJ.

### Detection of phospho-Thr737 and total Sch9 by western blot

Phospho-Thr737-Sch9 and anti-732–743 Sch9 antibodies were used to monitor Sch9 phosphorylation at Thr737 and total Sch9 protein by SDS-PAGE, respectively (Kingsbury et al., 2014). As positive and negative controls, WT cells growing in YPL medium were treated with either cycloheximide (25 µg/ml) or rapamycin (200 nM), respectively, for 30 min prior to switching to SL medium.

### co-IP

At the indicated time points, 50 OD_600_ units of cells were collected, pelleted, and stored at −80°C until cell lysis. Cell pellets were resuspended in 350 μl of lysis buffer A (50 mM HEPES, pH 7.5, 150 mM NaCl, 1 mM EDTA, 0.5% NP-40, 2× protease inhibitor cocktail, 1 mM PMSF, 1 mM Na_3_VO_4_⋅2H_2_O, 10 μM pepstatin A, 10 μM leupeptin, and 5 mM NaF) and subjected to five rounds of bead beating (30 s of beating/2 min of cooling on ice). The lysates were collected using the hole-punch method by centrifugation at 6,000 rpm for 2 min at 4°C, diluted with 525 μl of lysis buffer B (buffer A devoid of NP-40), and clarified by two successive centrifugation spins at maximum speed for 10 min at 4°C. The protein concentrations of the lysates were measured and normalized using the bicinchoninic acid assay. For input samples, 30 μl of the lysates was mixed with 15 μl of the sample buffer, denatured for 5 min at 65°C, and frozen until further use. For each co-IP reaction, 25 μl of Dynabeads Protein G (Life Technologies) were washed with a buffer A/B mixture (2:3 ratio, A:B) and incubated with 5 μg of mouse anti-Flag antibody (M2; Sigma-Aldrich) for 1–2 h at 4°C. Unbound antibody was removed by centrifugation at 500 × *g* for 1 min at 4°C. The Dynabeads-antibody slurries were then added to the cleared lysates and incubated for 1–2 h at 4°C. The slurries were then washed three times with wash buffer (50 mM HEPES, pH 7.5, 150 mM NaCl, 1 mM EDTA, and 0.2% NP-40) to remove unbound proteins. RNase treatment was performed by resuspending washed slurries in 50 μl 1× NEBuffer 3 (New England Biolabs) and incubating with 1 μl of RNase I_f_ (New England Biolabs) for 15 min at 37°C. IP samples were collected by boiling the beads in sample buffer for 5 min at 95°C.

### Protein expression and purification

Full-length Mkt1 and His-tagged Pbp1 (aa 482–550) were cloned into the petDuet plasmid and co-expressed in Rosetta cells cultured in 1 liter of Terrific broth. Expression was induced by adding 1 mM IPTG and incubating overnight at 16°C. Cells were spun down and resuspended in 60 ml of lysis buffer (50 mM Tris-HCl, pH 8, 500 mM NaCl, 10% glycerol, 1% NP-40, 5 mM imidazole, pH 8, 1 mM TCEP, 1X protease inhibitor cocktail, 5 mM PMSF, 0.625 mg/ml lysozyme, and 2 μl benzonase). The sample was lysed using a handheld homogenizer, and the supernatant was collected after spinning at 50,000 × *g* for 20 min and filtered. The filtered supernatant was added to 4 ml Ni Sepharose 6 Fast Flow (Cytiva) and gently tilted at 4°C for 1 h. The flow-through was passed twice through the column, the latter was washed with 25 column volumes of lysis buffer, and then the complex was eluted by passing 6.25–12.5 column volumes of elution buffers (50 mM Tris, pH 8, 500 mM NaCl, 10% glycerol, and 1 mM TCEP) with imidazole concentrations ranging from 50 to 500 mM. The complex was concentrated using a 50-ml ultracentrifugal filter with a 50-kDa molecular weight cutoff and further purified by gel filtration using a HiPrep 26/60 Sephacryl S-200 High Resolution column (Cytiva) equilibrated in elution buffer containing no imidazole.

### RT-qPCR analysis

For each sample, 1 ml of cells was collected, flash-frozen, and stored at −80°C until further use. Total RNA was extracted using the MasterPure Yeast RNA Purification Kit (LGC BioSearch Technologies) following the manufacturer’s protocol. cDNA was synthesized using the Superscript III reverse transcriptase (Invitrogen) and random hexamers, and gene expression levels were analyzed on a CFX384 Real-Time System (BioRad) with SYBR Green (Invitrogen). Relative mRNA levels were calculated using the ΔΔCq method and normalized to actin mRNA levels. RT-PCR targets and primer sequences used: *ACT1* (F: 5′-TCC​GGT​GAT​GGT​GTT​ACT​CA-3′; R: 5′-GGC​CAA​ATC​GAT​TCT​CAA​AA-3′), *ATP2* (5′-CCC​AGT​TGG​GAG​AGA​AAC​TTT​A-3′; R: 5′-CTG​CGT​GAA​TTG​GCT​TTC​TTA​G-3′), *COX17* (F: 5′-CCA​GAA​AAG​GAG​GAG​CGG​GAT​A-3′; R: 5′-CGA​AGC​CAT​AAC​CCT​TCA​TGC​AC-3′), *MEF1* (F: 5′-ACT​GAT​GGT​AGC​GTT​CAA​TAC​TC-3′; R: 5′-CAC​TCT​GAA​TGT​AGG​GTC​TTC​C-3′), *POR1* (F: 5′-GGC​TAC​AAT​GAA​CTG​CAA​ACT​AC-3′; R: 5′-AAT​CGG​ACA​CCT​TAG​CCT​TAA​C-3′), and *RSM19* (F: 5′-ACC​GGC​AGC​TAG​ACT​TTT​ATC-3′; R: 5′-ATT​GGA​GTG​CCC​TTA​GTC​ATG-3′).

### Polysome profiling

Polysome profiling was performed as previously described (Liu et al., 2021a). Upon sample collection, cycloheximide (0.1 mg ml^−1^ final concentration) was added to block translation elongation, followed by ice pellets (2 g/10 ml medium) to rapidly chill cells for 5 min. Cells were centrifuged at 4°C, snap frozen in liquid nitrogen, and stored at −80°C until processing. Frozen cell pellets were thawed on ice, washed twice in polysome extraction buffer (20 mM Tris-HCl, pH 7.5, 140 mM KCl, 5 mM MgCl_2_, 0.1 mg ml^−1^ cycloheximide, 1% Triton X-100, and 0.5 mM DTT), and lysed by three rounds of bead beating (30 s of beating/2 min of cooling on ice). Lysates were then clarified by centrifugation at 8,000 × *g* for 5 min at 4°C, and the supernatants were collected and concentrations measured by spectrophotometry at 260 nm. 10–50% (wt/vol) sucrose gradients were prepared in polysome extraction buffer devoid of Triton X-100 using the BIOCOMP Gradient Station *ip*. Approximately one A_260_ unit of cell lysate was layered onto the top of each sucrose gradient and centrifuged at 41,000 × *g* for 2 h at 4°C. Polysome profiles were recorded using BIOCOMP Gradient Station *ip* by measuring absorbance at 260 nm. Fractions were collected in tubes containing 1.4 ml of ethanol and 1 μl of glycogen and left at −20°C overnight. Fractions were then spun at 14,000 × *g* for 15 min, the supernatants were aspirated, and the pellets were washed in 70% ethanol. Pellets were spun at 14,000 × *g* for 15 min, supernatants were removed, and the pellets were left to air-dry and then resuspended in SDS-PAGE loading buffer.

### Microscopy image acquisition and processing

Single-plane images were acquired using a Zeiss Axio Observer.Z1 inverted confocal fluorescence microscope (Carl Zeiss Microscopy GmbH) equipped with a Plan-Apochromat 100×/1.40 oil DIC M27 objective. Yeast cells were imaged live at room temperature (∼22°C). Cells grown in YPL medium were resuspended in SCL medium prior to imaging, while cells grown in SL medium were imaged directly. Immersion oil (Immersol 518 F, *n* = 1.518 at 23°C; Carl Zeiss Microscopy GmbH) was used for the objective. Imaging was performed using three tracks: transmitted light (T-PMT-T1; 300–900 nm), mCherry (excitation 587 nm, emission 610 nm; 543–658-nm detection), and EGFP (excitation 488 nm, emission 509 nm; 481–543-nm detection). Fluorescence was detected using photomultiplier tubes (PMTs): GaAsP PMTs (600 V for mCherry and 750 V for EGFP) and a multialkali PMT (340 V) for transmitted light. Excitation was provided by 405-nm (0.5%), 488-nm (7.0%), and 561-nm (7.0%) lasers via standard dichroic mirrors (MBS 405 and MBS 488/561). Images were acquired in bidirectional scan mode with a zoom factor of 2.0, line averaging of 1×, and 0.67-µs pixel dwell time at 512 × 512 resolution (0.083 µm/pixel; 42.43 × 42.43-µm field of view). The confocal pinhole was set to 1 Airy unit. Data were acquired in 16-bit depth using ZEN Blue software and saved in uncompressed .czi format. Postacquisition processing was performed in ZEN (LSM Plus), with only linear brightness/contrast adjustments and channel merging. Raw .czi files were imported into Fiji using the Bio-Formats Importer with autoscaling disabled and channels split. For each channel, fixed brightness and contrast values were applied uniformly across all images.

### Alkaline phosphatase activity assays

Protocol used was previously described with some modifications (Noda et al., 1995; Yang et al., 2019). General autophagy and mitophagy were assessed in cells that express Pho8Δ60p or mitochondria outer membrane–targeted Om-pho8Δ60p, respectively. Cell pellets for each strain were collected in quadruplicate and resuspended in 400 μl of lysis buffer (250 mM Tris-HCl, pH 9, 25 mM MgSO_4_, 1% Triton X-100, and 2× protease inhibitor cocktail) and lysed by three rounds of bead beating (1 min of beating/1 min of cooling on ice). Cell lysates were clarified by centrifugation at maximum speed for 10 min at 4°C. For each sample, 70 μl of lysate was added in triplicate to a 96-well, flat-bottom plate, which was kept on ice before adding substrate. Reactions were initiated by adding 70 μl of substrate solution (250 mM Tris-HCl, pH 9, 25 mM MgSO_4_, 1% Triton X-100, and 2.7 mM *p*-nitrophenyl phosphate [MP Biomedicals Life Sciences]) and allowed to proceed at room temperature for 5 min before halting with 140 μl of stop buffer (1 M glycine, pH 11). The plate was read at 400 nm to measure the production of *p*-nitrophenol.

### Protein sequence alignments

Protein sequence alignments were generated using the online Clustal Omega program (Madeira et al., 2024). Pbp1 sequences were obtained by accessing the following UniProt profiles: *S*. *pombe* (Q9USW1), *Kluyveromyces lactis* (A0A5P2UCT88), *Kluyveromyces marxianus* (W0T9L5), *Zygosaccharomyces rouxii* (C5DRS7), *Saccharomyces eubayanus* (A0A0L8RJR8), *Saccharomyces kudriavzevii* (J4TY66), *Saccharomyces paradoxus* (A0A8B8URZ4), *S. cerevisiae* (P53297), *Yarrowia lipolytica* (A0A1D8N902), *Candida parapsilosis* (G8BH42), *Candida albicans* (A0A8H6C640), and *Candida dubliniensis* (B9WCZ5).

### Protein structure prediction analyses

The *S. cerevisiae* Mkt1 and Pbp1 AlphaFold structures were accessed from the AlphaFold Protein Structure Database (Jumper et al., 2021; Varadi et al., 2024). Complex structure predictions were performed by submitting *S. cerevisiae* Mkt1 (G30/K453 allele) and Pbp1 sequences to the AlphaFold Server (Abramson et al., 2024). Models and confidence scores depicted are from the top ranked predictions. Foldseek was accessed through the *S. cerevisiae* Mkt1 UniProt profile (P53297) to search for structurally similar proteins in the *Drosophila*, mouse, and human AFDB proteomes (van Kempen et al., 2024).

### Proteomics methods

#### IP/MS experiments to identify protein interactors

IP samples were collected and prepared in the same manner as described above. In-gel samples were digested overnight with trypsin (Pierce) following reduction and alkylation with DTT and iodoacetamide (Sigma-Aldrich). The samples then underwent solid-phase extraction cleanup with an Oasis HLB plate (Waters), and the resulting samples were injected onto an Orbitrap Fusion Lumos mass spectrometer coupled to an Ultimate 3000 RSLC-Nano liquid chromatography system. Samples were injected onto a 75 μm-i.d., 75-cm–long EasySpray column (Thermo Fisher Scientific) and eluted with a gradient from 0 to 28% buffer B over 90 min. Buffer A contained 2% (vol/vol) ACN and 0.1% formic acid in water, and buffer B contained 80% (vol/vol) ACN, 10% (vol/vol) trifluoroethanol, and 0.1% formic acid in water. The mass spectrometer operated in positive ion mode with a source voltage of 2.5 kV and an ion transfer tube temperature of 300°C. MS scans were acquired at 120,000 resolution in the Orbitrap, and MS/MS spectra were obtained for up to 1 s in the ion trap for each full spectrum acquired using higher-energy collisional dissociation for ions with charges 2–7. Dynamic exclusion was set for 30 s after an ion was selected for fragmentation.

Raw MS data files were analyzed using Proteome Discoverer v. 3.0 (Thermo Fisher Scientific), with peptide identification performed using a trypsin digest search with Sequest HT (cleavage after Lys and Arg except when followed by Pro). The *S*. *cerevisiae* protein database from UniProt (downloaded Sept. 21, 2023, 6,650 entries) was used. Fragment and precursor tolerances of 10 ppm and 0.6 Da were specified, and three missed cleavages were allowed. A minimum peptide length of six residues was required, and at least two unique peptides were required for positive protein identification. Carbamidomethylation of Cys was set as a fixed modification, and oxidation of Met was set as a variable modification. The false discovery rate cutoff was 1% for all peptides and proteins. Peptide peak intensities were summed for all peptides matched to a protein for protein quantitation.

#### TMT/MS experiments to determine protein abundances

Protein lysates were prepared from ∼5 OD_600_ units of cells using the TCA lysis method described above, and 15 μg of each lysate was used for downstream TMT/MS sample preparation. Following disulfide bond reduction and alkylation, samples were digested overnight with trypsin using an S-Trap (Protifi). The peptide eluate from the S-Trap was dried and reconstituted in 100 mM TEAB buffer. For the TMT9-plex and TMTpro18-plex experiments, TMT10-plex and TMTpro18-plex kits (Thermo Fisher Scientific) were used to label the samples as per the manufacturer’s instructions (TMT131 label not used for TMT9-plex samples), and equal amounts of each sample was mixed based on NanoDrop A205 (Thermo Fisher Scientific) values. The combined samples then underwent solid-phase extraction cleanup with an Oasis HLB plate (Waters) and were dried in a SpeedVac. The samples were then reconstituted in a 2% acetonitrile and 0.1% TFA buffer and diluted such that ∼1 μg of peptides were injected.

Peptides were analyzed on a Thermo Orbitrap Eclipse MS system coupled to an Ultimate 3000 RSLC-Nano liquid chromatography system. Samples were injected onto a 75-μm i.d., 75-cm–long EasySpray column (Thermo Fisher Scientific) and eluted with a gradient from 0 to 28% buffer B over 180 min at a flow rate of 250 nl/min. Buffer A contained 2% (vol/vol) ACN and 0.1% formic acid in water, and buffer B contained 80% (vol/vol) ACN, 10% (vol/vol) trifluoroethanol, and 0.1% formic acid in water at a flow rate of 250 nl/min. Spectra were continuously acquired in a data-dependent manner throughout the gradient, acquiring a full scan in the Orbitrap (at 120,000 resolution with a standard AGC target) followed by MS/MS scans on the most abundant ions in 2.5 s in the ion trap (turbo scan type with an intensity threshold of 5,000, CID collision energy of 35%, standard AGC target, maximum injection time of 35 ms, and isolation width of 0.7 m/z). Charge states from two to six were included. Dynamic exclusion was enabled with a repeat count of 1, an exclusion duration of 25 s, and an exclusion mass width of ±10 ppm. Real-time search was used for selection of peaks for SPS-MS3 analysis, with searched performed against the *S*. *cerevisiae* protein database from UniProt (downloaded Sept. 21, 2023, 6,650 entries). Up to 2 missed tryptic cleavages were allowed, with carbamidomethylation (+57.0215) of cysteine and either TMT reagent (+229.1629) or TMTpro reagent (+304.2071) of lysine and peptide N termini used as static modifications, and oxidation (+15.9949) of methionine used as a variable modification. MS3 data were collected for up to 10 MS2 peaks, which matched to fragments from the real-time peptide search identification, in the Orbitrap at a resolution of 50,000, higher-energy collisional dissociation collision energy of either 65% for TMT or 55% for TMTpro, and a scan range of 100–500.

Raw MS data files were analyzed using Proteome Discoverer v. 3.0 (Thermo Fisher Scientific), with peptide identification performed using a trypsin digest search with both Comet and Sequest HT (cleavage after Lys and Arg except when followed by Pro). The *S. cerevisiae* protein database from UniProt (downloaded Sept. 21, 2023, 6,650 entries) was used. Fragment and precursor tolerances of 10 ppm and 0.6 Da were specified, and up to 2 missed tryptic cleavages were allowed. Carbamidomethylation (+57.0215) of cysteine and either TMT reagent (+229.1629) or TMTpro reagent (+304.2071) of lysine and peptide N termini were used as static modifications, with oxidation (+15.9949) of methionine used as a variable modification. A minimum peptide length of six residues was required, and at least two unique peptides were required for positive protein identification. Carbamidomethylation of Cys was set as a fixed modification, and oxidation of Met was set as a variable modification. The false discovery rate cutoff was 1% for all peptides and proteins. Reporter ion peak intensities were summed for all peptides matched to a protein for protein quantitation.

### Gene ontology analysis

The Gene Ontology Resource GO Enrichment Analysis tool (https://geneontology.org) was used (Ashburner et al., 2000; Aleksander et al., 2023). Analyses were performed with Fisher’s exact test with Bonferroni correction for multiple testing.

### Statistical analyses

For TMT-MS experiments, the unpaired, two-sided *t* test was performed to calculate P values. Fisher’s exact test was used for qualitative gene set analyses of TMT-MS experiment in Fig. 3. The unpaired, two-sided *t* test was also performed to calculate P values for RT-qPCR, growth curve, and alkaline phosphatase assays, and pooled data are presented as mean ± SD. Significance cutoff was P < 0.05 for all statistical tests. The number of biological replicates (*n*) for each experiment is reported in the corresponding figure legend.

### Online supplemental material

Fig. S1 shows the AlphaFold complex prediction of Mkt1 and Pbp1 full-length proteins, AlphaFold complex predictions of full-length Mkt1 and Pbp1 fragments, and an alignment of yeast Pbp1 protein sequences representing putative MBRs. Fig. S2 shows the AlphaFold prediction of Mkt1 and a co-IP experiment testing the interactions between truncated Mkt1 proteins and Pbp1. Fig. S3 shows the mitochondrial protein levels in Mkt1 and Pbp1 truncation stains during growth in YPD and YPL media. Fig. S4 shows the polysome profiles of yeast cells collected 3 h following switch from YPD to YPL. Fig. S5 shows the GFP cleavage assays using Mkt1 and Pbp1 truncation strains cultured in SL medium. Table S1 shows the lists of proteins detected in IP/MS experiment with Mkt1-Flag and Pbp1-Flag IP lysates (related to Fig. 1). Table S2 shows the lists of proteins detected in IP/MS experiment with silver stain gel slices corresponding to Mkt1-Flag and Pbp1-Flag proteins (related to Fig. 1). Table S3 shows the Foldseek results for *S. cerevisiae* Mkt1 homologs in *Drosophila*, mouse, and human AFDB proteomes. Table S4 shows the lists of proteins detected and associated GO term analyses from TMT-MS experiment with *mkt1∆* and *pbp1∆* cells cultured in YPD and YPL media (related to Fig. 3). Table S5 shows the lists of proteins detected and associated GO term analyses from TMT-MS experiment with *mkt1∆* and *pbp1∆* cells cultured in SL medium (related to Fig. 6). Table S6 shows the list of yeast genetics and QTL studies reporting associations between Mkt1 and complex phenotypes. Table S7 shows the list of strains used in this study.

## Supplementary Material

Table S1 shows the lists of proteins detected in IP/MS experiment with Mkt1-Flag and Pbp1-Flag IP lysates (related to Fig. 1).

Table S2 shows the lists of proteins detected in IP/MS experiment with silver stain gel slices corresponding to Mkt1-Flag and Pbp1-Flag proteins (related to Fig. 1).

Table S3 shows the Foldseek results for *S. cerevisiae* Mkt1 homologs in *Drosophila*, mouse, and human AFDB proteomes.

Table S4 shows the lists of proteins detected and associated GO term analyses from TMT-MS experiment with *mkt1∆* and *pbp1∆* cells cultured in YPD and YPL media (related to Fig. 3).

Table S5 shows the lists of proteins detected and associated GO term analyses from TMT-MS experiment with *mkt1∆* and *pbp1∆* cells cultured in SL medium (related to Fig. 6).

Table S6 shows the list of yeast genetics and QTL studies reporting associations between Mkt1 and complex phenotypes.

Table S7 shows the list of strains used in this study.

SourceData F1 is the source file for Fig. 1.

SourceData F2 is the source file for Fig. 2.

SourceData F3 is the source file for Fig. 3.

SourceData F4 is the source file for Fig. 4.

SourceData F5 is the source file for Fig. 5.

SourceData F6 is the source file for Fig. 6.

SourceData F7 is the source file for Fig. 7.

SourceData F8 is the source file for Fig. 8.

SourceData F9 is the source file for Fig. 9.

SourceData FS2 is the source file for Fig. S2.

SourceData FS3 is the source file for Fig. S3.

SourceData FS4 is the source file for Fig. S4.

SourceData FS5 is the source file for Fig. S5.

## Data Availability

Primary data not available in the published article and its online supplemental material can be requested by contacting the corresponding author.

## References

[bib1] Abramson, J., J. Adler, J. Dunger, R. Evans, T. Green, A. Pritzel, O. Ronneberger, L. Willmore, A.J. Ballard, J. Bambrick, . 2024. Accurate structure prediction of biomolecular interactions with AlphaFold 3. Nature. 630:493–500. 10.1038/s41586-024-07487-w 38718835 PMC11168924

[bib2] Aleksander, S.A., S.A. Aleksander, J. Balhoff, S. Carbon, J.M. Cherry, H.J. Drabkin, D. Ebert, M. Feuermann, P. Gaudet, N.L. Harris, . 2023. The gene ontology knowledgebase in 2023. Genetics. 224:iyad031. 10.1093/genetics/iyad031 36866529 PMC10158837

[bib3] Anderson, J.B., J. Funt, D.A. Thompson, S. Prabhu, A. Socha, C. Sirjusingh, J.R. Dettman, L. Parreiras, D.S. Guttman, A. Regev, and L.M. Kohn. 2010. Determinants of divergent adaptation and dobzhansky-Muller interaction in experimental yeast populations. Curr. Biol. 20:1383–1388. 10.1016/j.cub.2010.06.022 20637622 PMC2938792

[bib4] Andrews, A.M., H.J. Mccartney, T.M. Errington, A.D. D’andrea, and I.G. Macara. 2018. A senataxin-associated exonuclease SAN1 is required for resistance to DNA interstrand cross-links. Nat. Commun. 9:2592. 10.1038/s41467-018-05008-8 29968717 PMC6030175

[bib5] Ashburner, M., C.A. Ball, J.A. Blake, D. Botstein, H. Butler, J.M. Cherry, A.P. Davis, K. Dolinski, S.S. Dwight, J.T. Eppig, . 2000. Gene ontology: Tool for the unification of biology. The gene ontology consortium. Nat. Genet. 25:25–29. 10.1038/75556 10802651 PMC3037419

[bib6] Ayache, J., M. Bénard, M. Ernoult-Lange, N. Minshall, N. Standart, M. Kress, and D. Weil. 2015. P-body assembly requires DDX6 repression complexes rather than decay or Ataxin2/2L complexes. Mol. Biol. Cell. 26:2579–2595. 10.1091/mbc.E15-03-0136 25995375 PMC4501357

[bib7] Buschlen, S., J.-M. Amillet, B. Guiard, A. Fournier, C. Marcireau, and M. Bolotin-Fukuhara. 2003. The S. Cerevisiae HAP complex, a key regulator of mitochondrial function, coordinates nuclear and mitochondrial gene expression. Comp. Funct. Genomics. 4:37–46. 10.1002/cfg.254 18629096 PMC2447382

[bib8] Carroll, J.S., S.E. Munchel, and K. Weis. 2011. The DExD/H box ATPase Dhh1 functions in translational repression, mRNA decay, and processing body dynamics. J. Cell Biol. 194:527–537. 10.1083/jcb.201007151 21844211 PMC3160580

[bib9] Chaithanya, K.V., and H. Sinha. 2023. MKT1 alleles regulate stress responses through posttranscriptional modulation of Puf3 targets in budding yeast. Yeast. 40:616–627. 10.1002/yea.3908 37990816

[bib10] Chritton, J.J., and M. Wickens. 2010. Translational repression by PUF proteins in vitro. Rna. 16:1217–1225. 10.1261/rna.2070110 20427513 PMC2874173

[bib11] Collins, M.A., R. Avery, and F.W. Albert. 2023. Substrate-specific effects of natural genetic variation on proteasome activity. PLoS Genet. 19:e1010734. 10.1371/journal.pgen.1010734 37126494 PMC10174532

[bib12] Collins, M.A., G. Mekonnen, and F.W. Albert. 2022. Variation in ubiquitin system genes creates substrate-specific effects on proteasomal protein degradation. Elife. 11:e79570. 10.7554/eLife.79570 36218234 PMC9634822

[bib13] Demogines, A., E. Smith, L. Kruglyak, and E. Alani. 2008. Identification and dissection of a complex DNA repair sensitivity phenotype in Baker's yeast. PLoS Genet. 4:e1000123. 10.1371/journal.pgen.1000123 18617998 PMC2440805

[bib14] Deutschbauer, A.M., and R.W. Davis. 2005. Quantitative trait loci mapped to single-nucleotide resolution in yeast. Nat. Genet. 37:1333–1340. 10.1038/ng1674 16273108

[bib15] Dimitrov, L.N., R.B. Brem, L. Kruglyak, and D.E. Gottschling. 2009. Polymorphisms in multiple genes contribute to the spontaneous mitochondrial genome instability of Saccharomyces cerevisiae S288C strains. Genetics. 183:365–383. 10.1534/genetics.109.104497 19581448 PMC2746160

[bib16] Dunn, C.D., and R.E. Jensen. 2003. Suppression of a defect in mitochondrial protein import identifies cytosolic proteins required for viability of yeast cells lacking mitochondrial DNA. Genetics. 165:35–45. 10.1093/genetics/165.1.35 14504216 PMC1462761

[bib17] Ehrenreich, I.M., N. Torabi, Y. Jia, J. Kent, S. Martis, J.A. Shapiro, D. Gresham, A.A. Caudy, and L. Kruglyak. 2010. Dissection of genetically complex traits with extremely large pools of yeast segregants. Nature. 464:1039–1042. 10.1038/nature08923 20393561 PMC2862354

[bib18] Elden, A.C., H.-J. Kim, M.P. Hart, A.S. Chen-Plotkin, B.S. Johnson, X. Fang, M. Armakola, F. Geser, R. Greene, M.M. Lu, . 2010. Ataxin-2 intermediate-length polyglutamine expansions are associated with increased risk for ALS. Nature. 466:1069–1075. 10.1038/nature09320 20740007 PMC2965417

[bib19] Fleischer, T.C., C.M. Weaver, K.J. Mcafee, J.L. Jennings, and A.J. Link. 2006. Systematic identification and functional screens of uncharacterized proteins associated with eukaryotic ribosomal complexes. Genes Dev. 20:1294–1307. 10.1101/gad.1422006 16702403 PMC1472904

[bib20] Gagneur, J., O. Stegle, C. Zhu, P. Jakob, M.M. Tekkedil, R.S. Aiyar, A.K. Schuon, D. Pe'er, and L.M. Steinmetz. 2013. Genotype-environment interactions reveal causal pathways that mediate genetic effects on phenotype. PLoS Genet. 9:e1003803. 10.1371/journal.pgen.1003803 24068968 PMC3778020

[bib21] Gibney, P.A., A. Chen, A. Schieler, J.C. Chen, Y. Xu, D.G. Hendrickson, R.S. Mcisaac, J.D. Rabinowitz, and D. Botstein. 2020. A tps1Δ persister-like state in Saccharomyces cerevisiae is regulated by MKT1. PLoS One. 15:e0233779. 10.1371/journal.pone.0233779 32470059 PMC7259636

[bib22] Goldstrohm, A.C., B.A. Hook, D.J. Seay, and M. Wickens. 2006. PUF proteins bind Pop2p to regulate messenger RNAs. Nat. Struct. Mol. Biol. 13:533–539. 10.1038/nsmb1100 16715093

[bib23] Gou, L., J.S. Bloom, and L. Kruglyak. 2019. The genetic basis of mutation rate variation in yeast. Genetics. 211:731–740. 10.1534/genetics.118.301609 30504363 PMC6366923

[bib24] Gupta, S., A. Radhakrishnan, P. Raharja-Liu, G. Lin, L.M. Steinmetz, J. Gagneur, and H. Sinha. 2015. Temporal expression profiling identifies pathways mediating effect of causal variant on phenotype. PLoS Genet. 11:e1005195. 10.1371/journal.pgen.1005195 26039065 PMC4454590

[bib25] Hickman, M.J., and F. Winston. 2007. Heme levels switch the function of Hap1 of Saccharomyces cerevisiae between transcriptional activator and transcriptional repressor. Mol. Cell. Biol. 27:7414–7424. 10.1128/mcb.00887-07 17785431 PMC2169065

[bib26] Hou, J., and J. Schacherer. 2017. Fitness trade-offs lead to suppressor tolerance in yeast. Mol. Biol. Evol. 34:110–118. 10.1093/molbev/msw225 28007972 PMC5854122

[bib27] Jain, S., J.R. Wheeler, R.W. Walters, A. Agrawal, A. Barsic, and R. Parker. 2016. ATPase-modulated stress granules contain a diverse proteome and substructure. Cell. 164:487–498. 10.1016/j.cell.2015.12.038 26777405 PMC4733397

[bib28] Jonas, S., and E. Izaurralde. 2013. The role of disordered protein regions in the assembly of decapping complexes and RNP granules. Genes Dev. 27:2628–2641. 10.1101/gad.227843.113 24352420 PMC3877753

[bib29] Jumper, J., R. Evans, A. Pritzel, T. Green, M. Figurnov, O. Ronneberger, K. Tunyasuvunakool, R. Bates, A. Žídek, A. Potapenko, . 2021. Highly accurate protein structure prediction with AlphaFold. Nature. 596:583–589. 10.1038/s41586-021-03819-2 34265844 PMC8371605

[bib30] Kanki, T., and D.J. Klionsky. 2008. Mitophagy in yeast occurs through a selective mechanism. J. Biol. Chem. 283:32386–32393. 10.1074/jbc.M802403200 18818209 PMC2583303

[bib31] Kato, M., Y.-S. Yang, B.M. Sutter, Y. Wang, S.L. Mcknight, and B.P. Tu. 2019. Redox state controls phase separation of the yeast ataxin-2 protein via reversible oxidation of its methionine-rich low-complexity domain. Cell. 177:711–721.e8. 10.1016/j.cell.2019.02.044 30982603 PMC6752730

[bib32] Kelly, T.J., H.I. Suzuki, J.R. Zamudio, M. Suzuki, and P.A. Sharp. 2019. Sequestration of microRNA-mediated target repression by the Ago2-associated RNA-binding protein FAM120A. Rna. 25:1291–1297. 10.1261/rna.071621.119 31289130 PMC6800481

[bib33] Kim, H.S., and J.C. Fay. 2009. A combined-cross analysis reveals genes with drug-specific and background-dependent effects on drug sensitivity in Saccharomyces cerevisiae. Genetics. 183:1141–1151. 10.1534/genetics.109.108068 19720856 PMC2778966

[bib34] Kingsbury, J.M., N.D. Sen, T. Maeda, J. Heitman, and M.E. Cardenas. 2014. Endolysosomal membrane trafficking complexes drive nutrient-dependent TORC1 signaling to control cell growth in Saccharomyces cerevisiae. Genetics. 196:1077–1089. 10.1534/genetics.114.161646 24514902 PMC3982701

[bib35] Kohn, L.M., and J.B. Anderson. 2014. The underlying structure of adaptation under strong selection in 12 experimental yeast populations. Eukaryot. Cell. 13:1200–1206. 10.1128/ec.00122-14 25016004 PMC4187625

[bib36] Lapointe, C.P., J.A. Stefely, A. Jochem, P.D. Hutchins, G.M. Wilson, N.W. Kwiecien, J.J. Coon, M. Wickens, and D.J. Pagliarini. 2018. Multi-omics reveal specific targets of the RNA-binding protein Puf3p and its orchestration of mitochondrial biogenesis. Cell Syst. 6:125–135.e6. 10.1016/j.cels.2017.11.012 29248374 PMC5799006

[bib37] Lee, C.-D., and B.P. Tu. 2015. Glucose-regulated phosphorylation of the PUF protein Puf3 regulates the translational fate of its bound mRNAs and association with RNA granules. Cell Rep. 11:1638–1650. 10.1016/j.celrep.2015.05.014 26051939 PMC4472502

[bib38] Lee, D., T. Ohn, Y.-C. Chiang, G. Quigley, G. Yao, Y. Liu, and C.L. Denis. 2010. PUF3 acceleration of deadenylation in vivo can operate independently of CCR4 activity, possibly involving effects on the PAB1-mRNP structure. J. Mol. Biol. 399:562–575. 10.1016/j.jmb.2010.04.034 20435044 PMC2904828

[bib39] Lee, S.-I., A.M. Dudley, D. Drubin, P.A. Silver, N.J. Krogan, D. Pe’er, and D. Koller. 2009. Learning a prior on regulatory potential from eQTL data. PLoS Genet. 5:e1000358. 10.1371/journal.pgen.1000358 19180192 PMC2627940

[bib40] Lewis, J.A., A.T. Broman, J. Will, and A.P. Gasch. 2014. Genetic architecture of ethanol-responsive transcriptome variation in Saccharomyces cerevisiae strains. Genetics. 198:369–382. 10.1534/genetics.114.167429 24970865 PMC4174948

[bib41] Linder, R.A., F. Seidl, K. Ha, and I.M. Ehrenreich. 2016. The complex genetic and molecular basis of a model quantitative trait. Mol. Biol. Cell. 27:209–218. 10.1091/mbc.E15-06-0408 26510497 PMC4694759

[bib42] Liu, K., D.A. Santos, J.A. Hussmann, Y. Wang, B.M. Sutter, J.S. Weissman, and B.P. Tu. 2021a. Regulation of translation by methylation multiplicity of 18S rRNA. Cell Rep. 34:108825. 10.1016/j.celrep.2021.108825 33691096 PMC8063911

[bib43] Liu, S., S. Liu, B. He, L. Li, L. Li, J. Wang, T. Cai, S. Chen, and H. Jiang. 2021b. OXPHOS deficiency activates global adaptation pathways to maintain mitochondrial membrane potential. EMBO Rep. 22:e51606. 10.15252/embr.202051606 33655635 PMC8025004

[bib44] Longtine, M.S., A. McKenzie 3rd, D.J. Demarini, N.G. Shah, A. Wach, A. Brachat, P. Philippsen, and J.R. Pringle. 1998. Additional modules for versatile and economical PCR-based gene deletion and modification in Saccharomyces cerevisiae. Yeast. 14:953–961. 10.1002/(sici)1097-0061(199807)14:10<953::Aid-yea293>3.0.Co;2-u 9717241

[bib45] Madeira, F., N. Madhusoodanan, J. Lee, A. Eusebi, A. Niewielska, A.R.N. Tivey, R. Lopez, and S. Butcher. 2024. The EMBL-EBI job dispatcher sequence analysis tools framework in 2024. Nucleic Acids Res. 52:W521–w525. 10.1093/nar/gkae241 38597606 PMC11223882

[bib46] Mangus, D.A., N. Amrani, and A. Jacobson. 1998. Pbp1p, a factor interacting with Saccharomyces cerevisiae poly(A)-binding protein, regulates polyadenylation. Mol. Cell. Biol. 18:7383–7396. 10.1128/mcb.18.12.7383 9819425 PMC109320

[bib47] Mangus, D.A., M.M. Smith, J.M. Mcsweeney, and A. Jacobson. 2004. Identification of factors regulating poly(A) tail synthesis and maturation. Mol. Cell. Biol. 24:4196–4206. 10.1128/mcb.24.10.4196-4206.2004 15121841 PMC400472

[bib48] Melo Do Nascimento, L., M. Terrao, K.K. Marucha, B. Liu, F. Egler, and C. Clayton. 2020. The RNA-associated proteins MKT1 and MKT1L form alternative PBP1-containing complexes in Trypanosoma brucei. J. Biol. Chem. 295:10940–10955. 10.1074/jbc.RA120.013306 32532821 PMC7415969

[bib49] Noda, T., A. Matsuura, Y. Wada, and Y. Ohsumi. 1995. Novel system for monitoring autophagy in the yeast Saccharomyces cerevisiae. Biochem. Biophys. Res. Commun. 210:126–132. 10.1006/bbrc.1995.1636 7741731

[bib50] Nonhoff, U., M. Ralser, F. Welzel, I. Piccini, D. Balzereit, M.-L. Yaspo, H. Lehrach, and S. Krobitsch. 2007. Ataxin-2 interacts with the DEAD/H-box RNA helicase DDX6 and interferes with P-bodies and stress granules. Mol. Biol. Cell. 18:1385–1396. 10.1091/mbc.e06-12-1120 17392519 PMC1838996

[bib51] Olivas, W., and R. Parker. 2000. The Puf3 protein is a transcript-specific regulator of mRNA degradation in yeast. Embo J. 19:6602–6611. 10.1093/emboj/19.23.6602 11101532 PMC305854

[bib52] Parreiras, L.S., L.M. Kohn, and J.B. Anderson. 2011. Cellular effects and epistasis among three determinants of adaptation in experimental populations of Saccharomyces cerevisiae. Eukaryot. Cell. 10:1348–1356. 10.1128/ec.05083-11 21856932 PMC3187067

[bib53] Paysan-Lafosse, T., M. Blum, S. Chuguransky, T. Grego, B.L. Pinto, G.A. Salazar, M.L. Bileschi, P. Bork, A. Bridge, L. Colwell, . 2023. InterPro in 2022. Nucleic Acids Res. 51:D418–d427. 10.1093/nar/gkac993 36350672 PMC9825450

[bib54] Pulst, S.M., A. Nechiporuk, T. Nechiporuk, S. Gispert, X.N. Chen, I. Lopes-Cendes, S. Pearlman, S. Starkman, G. Orozco-Diaz, A. Lunkes, . 1996. Moderate expansion of a normally biallelic trinucleotide repeat in spinocerebellar ataxia type 2. Nat. Genet. 14:269–276. 10.1038/ng1196-269 8896555

[bib55] Sanpei, K., H. Takano, S. Igarashi, T. Sato, M. Oyake, H. Sasaki, A. Wakisaka, K. Tashiro, Y. Ishida, T. Ikeuchi, . 1996. Identification of the spinocerebellar ataxia type 2 gene using a direct identification of repeat expansion and cloning technique, DIRECT. Nat. Genet. 14:277–284. 10.1038/ng1196-277 8896556

[bib56] Satterfield, T.F., and L.J. Pallanck. 2006. Ataxin-2 and its Drosophila homolog, ATX2, physically assemble with polyribosomes. Hum. Mol. Genet. 15:2523–2532. 10.1093/hmg/ddl173 16835262

[bib57] Schell, R., J.J. Hale, M.N. Mullis, T. Matsui, R. Foree, and I.M. Ehrenreich. 2022. Genetic basis of a spontaneous mutation's expressivity. Genetics. 220:iyac013. 10.1093/genetics/iyac013 35078232 PMC8893249

[bib58] Singh, A., I. Minia, D. Droll, A. Fadda, C. Clayton, and E. Erben. 2014. Trypanosome MKT1 and the RNA-binding protein ZC3H11: Interactions and potential roles in post-transcriptional regulatory networks. Nucleic Acids Res. 42:4652–4668. 10.1093/nar/gkt1416 24470144 PMC3985637

[bib59] Sinha, H., B.P. Nicholson, L.M. Steinmetz, and J.H. Mccusker. 2006. Complex genetic interactions in a quantitative trait locus. PLoS Genet. 2:e13. 10.1371/journal.pgen.0020013 16462944 PMC1359075

[bib60] Son, Y.-E., C. Fu, W.-H. Jung, S.-H. Oh, J.-H. Kwak, M.E. Cardenas, J. Heitman, and H.-S. Park. 2019. Pbp1-Interacting protein Mkt1 regulates virulence and sexual reproduction in Cryptococcus neoformans. Front. Cell. Infect. Microbiol. 9:355. 10.3389/fcimb.2019.00355 31681631 PMC6811503

[bib61] Steinmetz, L.M., H. Sinha, D.R. Richards, J.I. Spiegelman, P.J. Oefner, J.H. Mccusker, and R.W. Davis. 2002. Dissecting the architecture of a quantitative trait locus in yeast. Nature. 416:326–330. 10.1038/416326a 11907579

[bib62] Swinnen, S., K. Schaerlaekens, T. Pais, J. Claesen, G. Hubmann, Y. Yang, M. Demeke, M.R. Foulquié-Moreno, A. Goovaerts, K. Souvereyns, . 2012. Identification of novel causative genes determining the complex trait of high ethanol tolerance in yeast using pooled-segregant whole-genome sequence analysis. Genome Res. 22:975–984. 10.1101/gr.131698.111 22399573 PMC3337442

[bib63] Swisher, K.D., and R. Parker. 2010. Localization to, and effects of Pbp1, Pbp4, Lsm12, Dhh1, and Pab1 on stress granules in Saccharomyces cerevisiae. PLoS One. 5:e10006. 10.1371/journal.pone.0010006 20368989 PMC2848848

[bib64] Tadauchi, T., T. Inada, K. Matsumoto, and K. Irie. 2004. Posttranscriptional regulation of HO expression by the Mkt1-Pbp1 complex. Mol. Cell. Biol. 24:3670–3681. 10.1128/mcb.24.9.3670-3681.2004 15082763 PMC387745

[bib65] Taglini, F., E. Chapman, R. van Nues, E. Theron, and E.H. Bayne. 2020. Mkt1 is required for RNAi-mediated silencing and establishment of heterochromatin in fission yeast. Nucleic Acids Res. 48:1239–1253. 10.1093/nar/gkz1157 31822915 PMC7026591

[bib66] Takahara, T., and T. Maeda. 2012. Transient sequestration of TORC1 into stress granules during heat stress. Mol. Cell. 47:242–252. 10.1016/j.molcel.2012.05.019 22727621

[bib67] Van De Poll, F., B.M. Sutter, M.G. Acoba, D. Caballero, S. Jahangiri, Y.-S. Yang, C.D. Lee, and B.P. Tu. 2023. Pbp1 associates with Puf3 and promotes translation of its target mRNAs involved in mitochondrial biogenesis. PLoS Genet. 19:e1010774. 10.1371/journal.pgen.1010774 37216416 PMC10237644

[bib68] van Dijken, J.P., J. Bauer, L. Brambilla, P. Duboc, J.M. Francois, C. Gancedo, M.L. Giuseppin, J.J. Heijnen, M. Hoare, H.C. Lange, . 2000. An interlaboratory comparison of physiological and genetic properties of four Saccharomyces cerevisiae strains. Enzyme Microb. Technol. 26:706–714. 10.1016/s0141-0229(00)00162-9 10862876

[bib69] van Kempen, M., S.S. Kim, C. Tumescheit, M. Mirdita, J. Lee, C.L.M. Gilchrist, J. Söding, and M. Steinegger. 2024. Fast and accurate protein structure search with Foldseek. Nat. Biotechnol. 42:243–246. 10.1038/s41587-023-01773-0 37156916 PMC10869269

[bib70] Varadi, M., D. Bertoni, P. Magana, U. Paramval, I. Pidruchna, M. Radhakrishnan, M. Tsenkov, S. Nair, M. Mirdita, J. Yeo, . 2024. AlphaFold protein structure database in 2024: Providing structure coverage for over 214 million protein sequences. Nucleic Acids Res. 52:D368–d375. 10.1093/nar/gkad1011 37933859 PMC10767828

[bib71] Waldherr, M., A. Ragnini, B. Jank, R. Teply, G. Wiesenberger, and R.J. Schweyen. 1993. A multitude of suppressors of group II intron-splicing defects in yeast. Curr. Genet. 24:301–306. 10.1007/bf00336780 8252639

[bib72] Wang, X., and X.J. Chen. 2015. A cytosolic network suppressing mitochondria-mediated proteostatic stress and cell death. Nature. 524:481–484. 10.1038/nature14859 26192197 PMC4582408

[bib73] Wang, X., and L. Kruglyak. 2014. Genetic basis of haloperidol resistance in Saccharomyces cerevisiae is complex and dose dependent. PLoS Genet. 10:e1004894. 10.1371/journal.pgen.1004894 25521586 PMC4270474

[bib74] Weith, M., J. Großbach, M. Clement-Ziza, L. Gillet, M. Rodríguez-López, S. Marguerat, C.T. Workman, P. Picotti, J. Bähler, R. Aebersold, and A. Beyer. 2023. Genetic effects on molecular network states explain complex traits. Mol. Syst. Biol. 19:e11493. 10.15252/msb.202211493 37485750 PMC10407735

[bib75] Wickner, R.B. 1980. Plasmids controlled exclusion of the K2 killer double-stranded RNA plasmid of yeast. Cell. 21:217–226. 10.1016/0092-8674(80)90129-4 6996833

[bib76] Wickner, R.B. 1987. MKT1, a nonessential Saccharomyces cerevisiae gene with a temperature-dependent effect on replication of M2 double-stranded RNA. J. Bacteriol. 169:4941–4945. 10.1128/jb.169.11.4941-4945.1987 2822656 PMC213890

[bib77] Wilkening, S., G. Lin, E.S. Fritsch, M.M. Tekkedil, S. Anders, R. Kuehn, M. Nguyen, R.S. Aiyar, M. Proctor, N.A. Sakhanenko, . 2014. An evaluation of high-throughput approaches to QTL mapping in Saccharomyces cerevisiae. Genetics. 196:853–865. 10.1534/genetics.113.160291 24374355 PMC3948811

[bib78] Wu, X., and B.P. Tu. 2011. Selective regulation of autophagy by the Iml1-Npr2-Npr3 complex in the absence of nitrogen starvation. Mol. Biol. Cell. 22:4124–4133. 10.1091/mbc.E11-06-0525 21900499 PMC3204073

[bib79] Yang, Y., M.R. Foulquié-Moreno, L. Clement, E. Erdei, A. Tanghe, K. Schaerlaekens, F. Dumortier, and J.M. Thevelein. 2013. QTL analysis of high thermotolerance with superior and downgraded parental yeast strains reveals new minor QTLs and converges on novel causative alleles involved in RNA processing. PLoS Genet. 9:e1003693. 10.1371/journal.pgen.1003693 23966873 PMC3744412

[bib80] Yang, Y.-S., M. Kato, X. Wu, A. Litsios, B.M. Sutter, Y. Wang, C.-H. Hsu, N.E. Wood, A. Lemoff, H. Mirzaei, . 2019. Yeast Ataxin-2 forms an intracellular condensate required for the inhibition of TORC1 signaling during respiratory growth. Cell. 177:697–710.e17. 10.1016/j.cell.2019.02.043 30982600 PMC6752053

[bib81] Zhao, F., W. Kim, H. Gao, C. Liu, Y. Zhang, Y. Chen, M. Deng, Q. Zhou, J. Huang, Q. Hu, . 2021. ASTE1 promotes shieldin-complex-mediated DNA repair by attenuating end resection. Nat. Cell Biol. 23:894–904. 10.1038/s41556-021-00723-9 34354233

[bib82] Zhu, J., B. Zhang, E.N. Smith, B. Drees, R.B. Brem, L. Kruglyak, R.E. Bumgarner, and E.E. Schadt. 2008. Integrating large-scale functional genomic data to dissect the complexity of yeast regulatory networks. Nat. Genet. 40:854–861. 10.1038/ng.167 18552845 PMC2573859

