# Supplementary figures and images for: The yeast Mkt1/Pbp1 complex promotes adaptive responses to respiratory growth
Source: J Cell Biol. 2025 Aug 13;224(10):e202411169. doi: 10.1083/jcb.202411169 (PMC12345631; doi:10.1083/jcb.202411169)

A

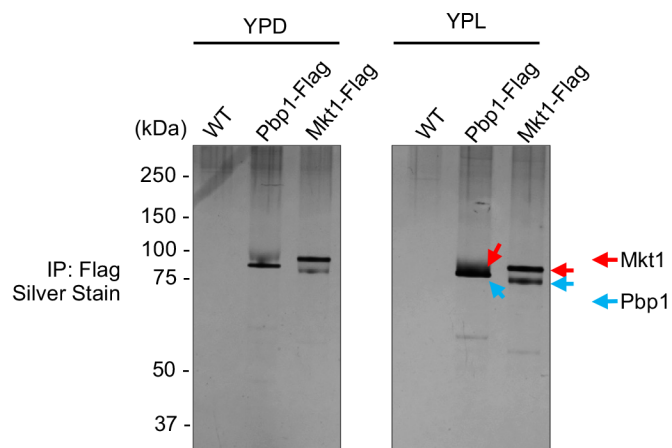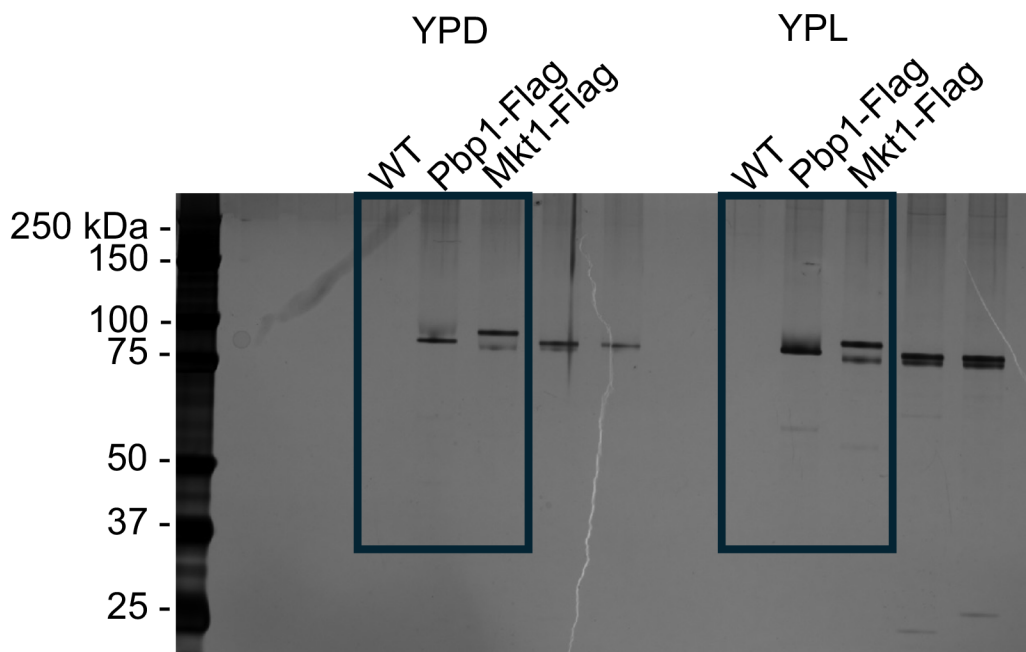

C

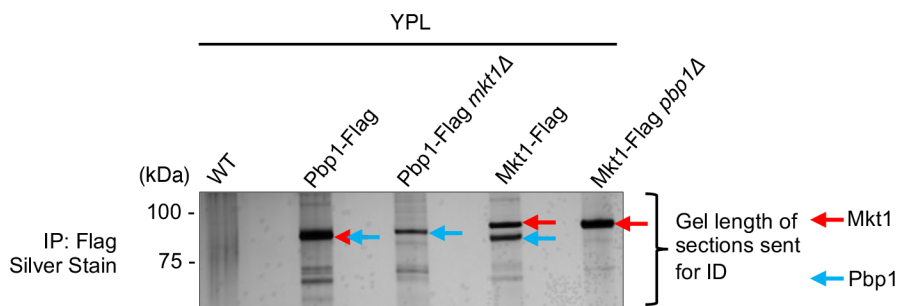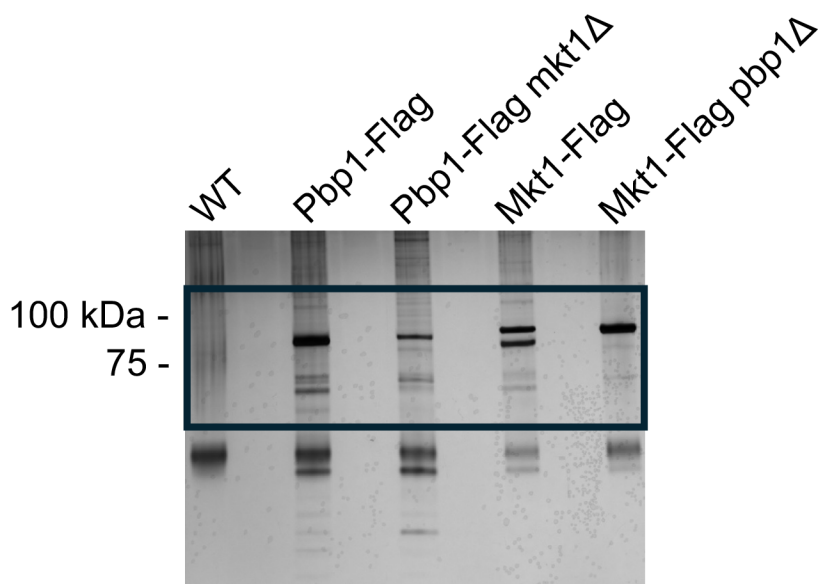

D

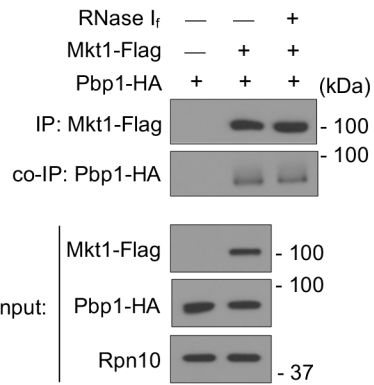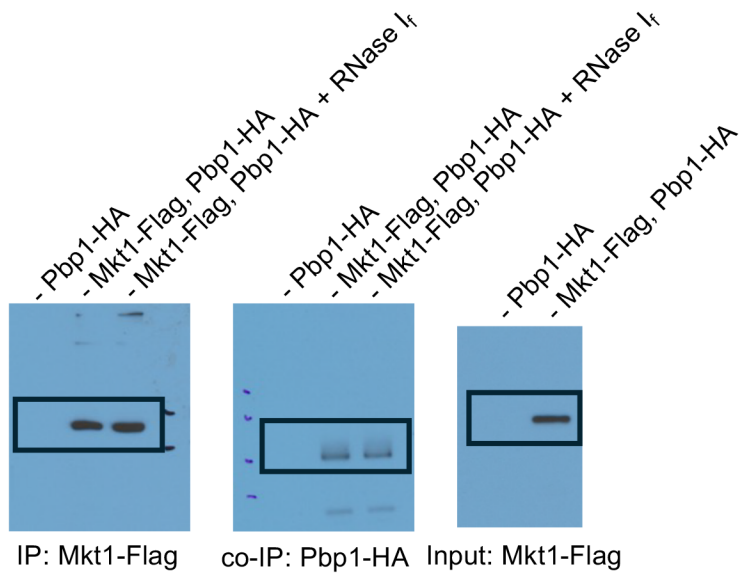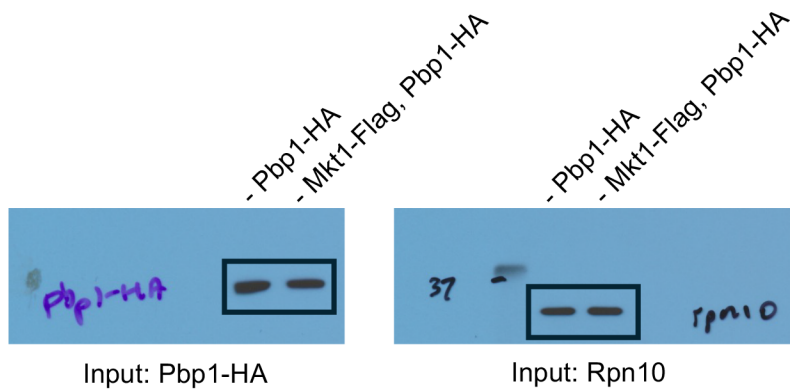

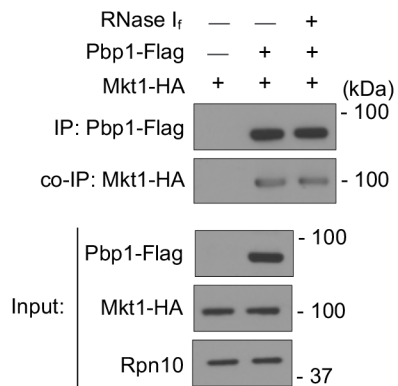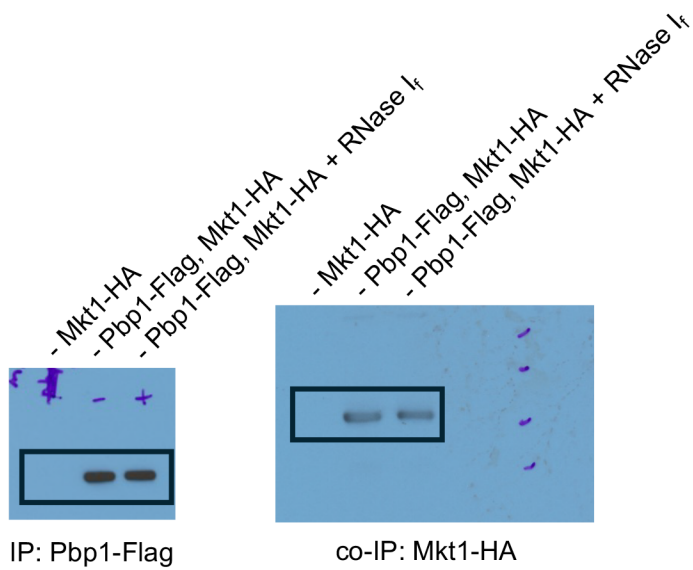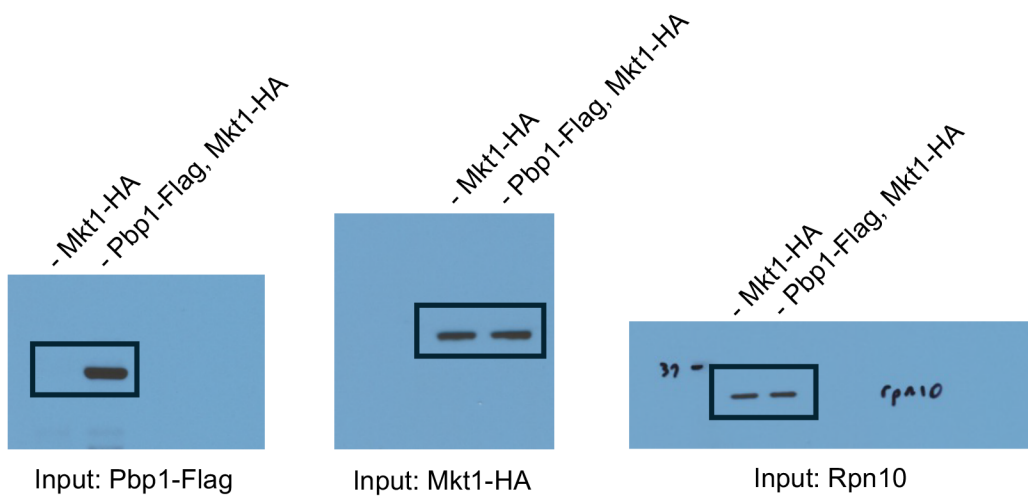

Figure 1A, Additional experiment

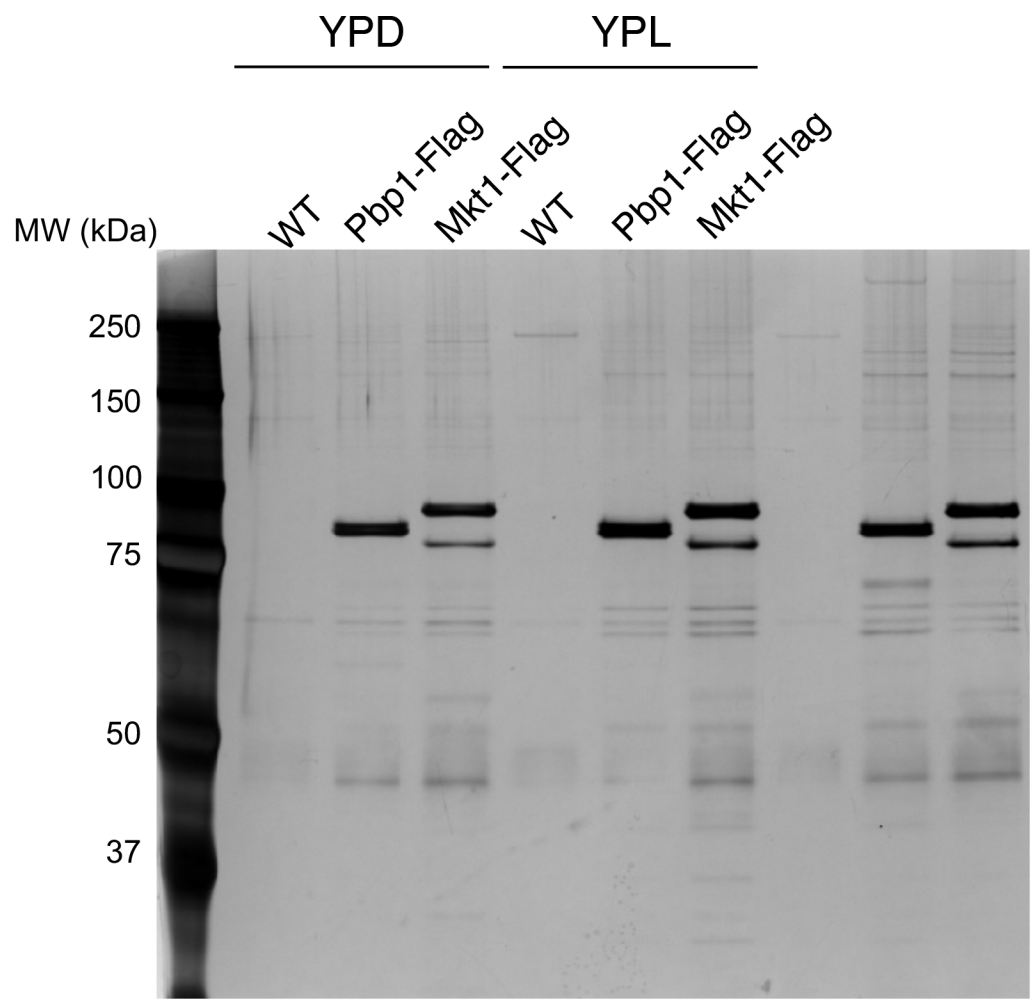

Supplement: SourceData F1 — is the source file for Fig. 1. [file jcb_202411169_sourcedataf1.pdf]

A

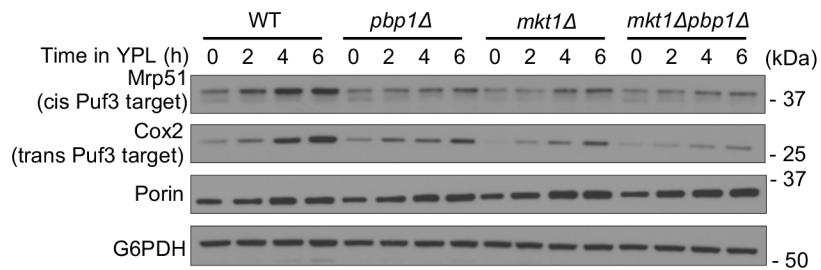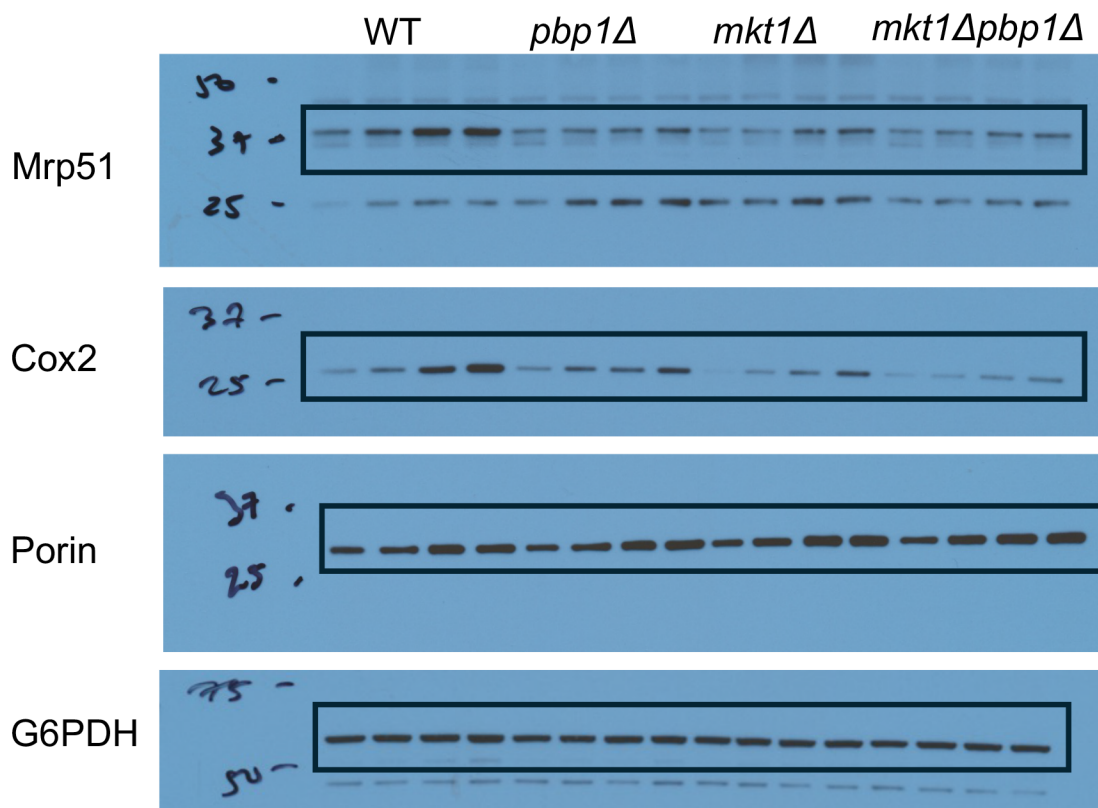

H

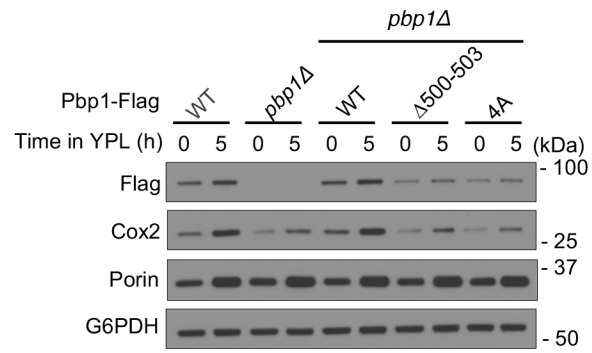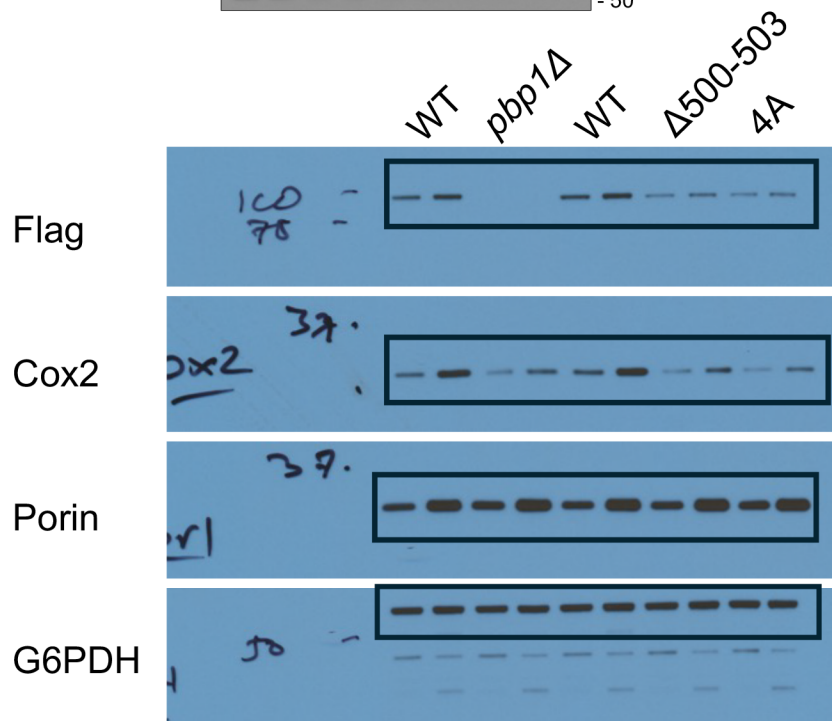

Figure 3A, Additional experiment

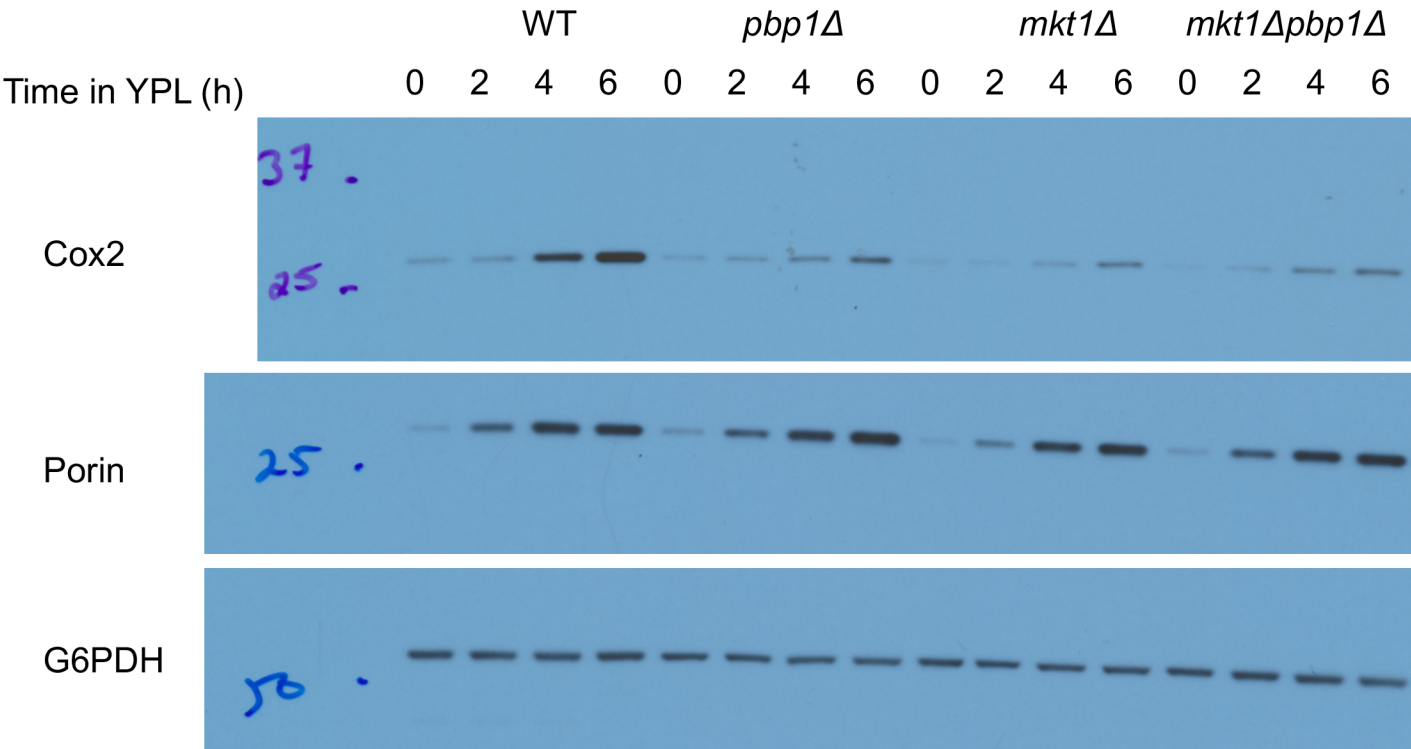

Supplement: SourceData F3 — is the source file for Fig. 3. [file jcb_202411169_sourcedataf3.pdf]

A

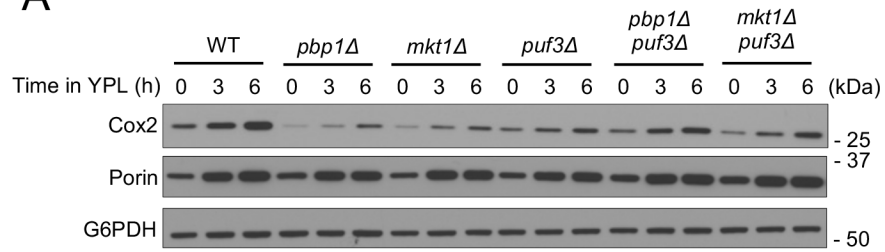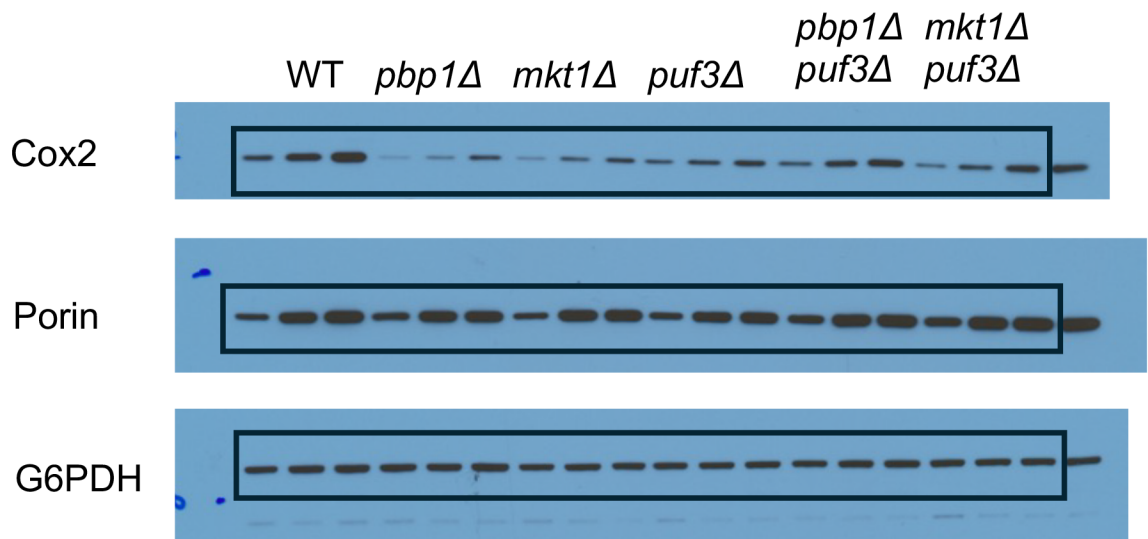

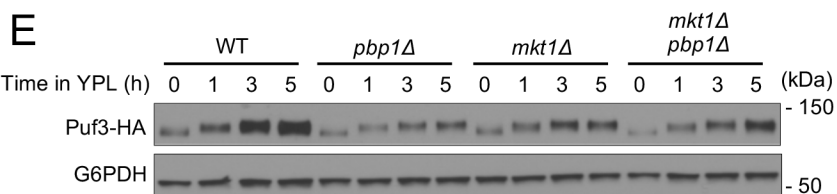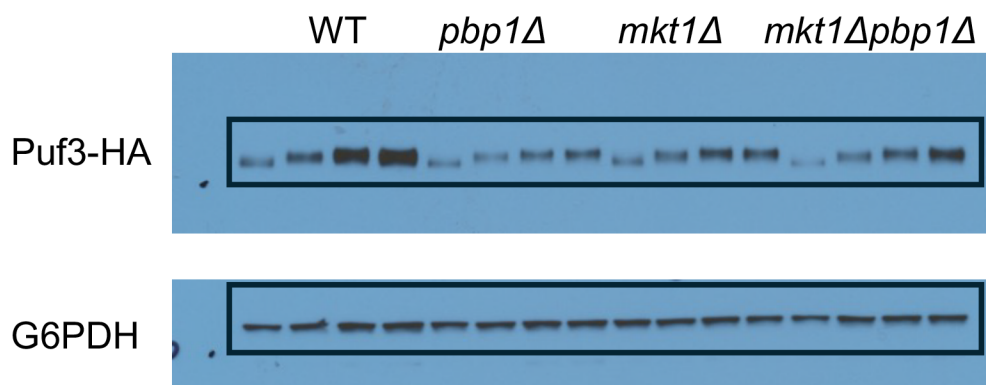

F

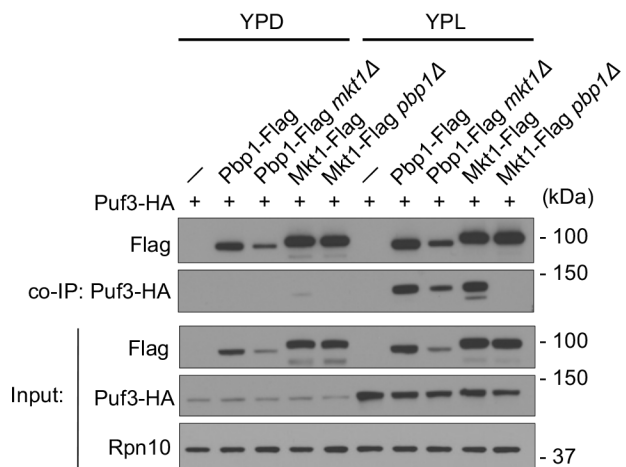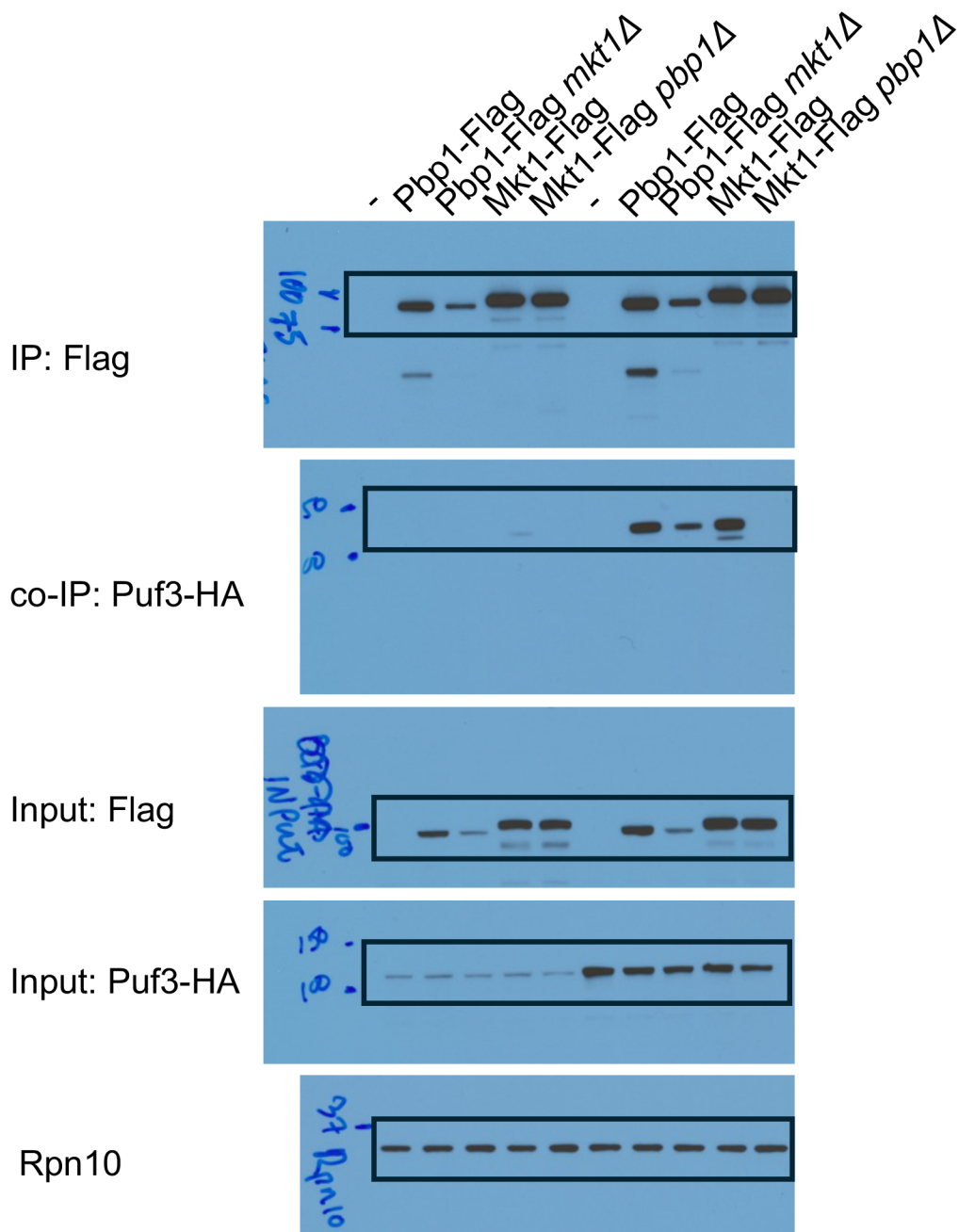

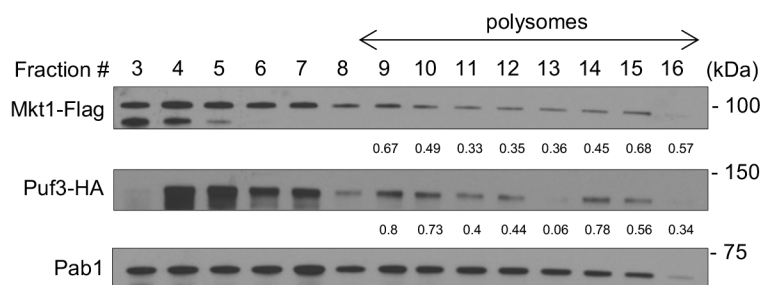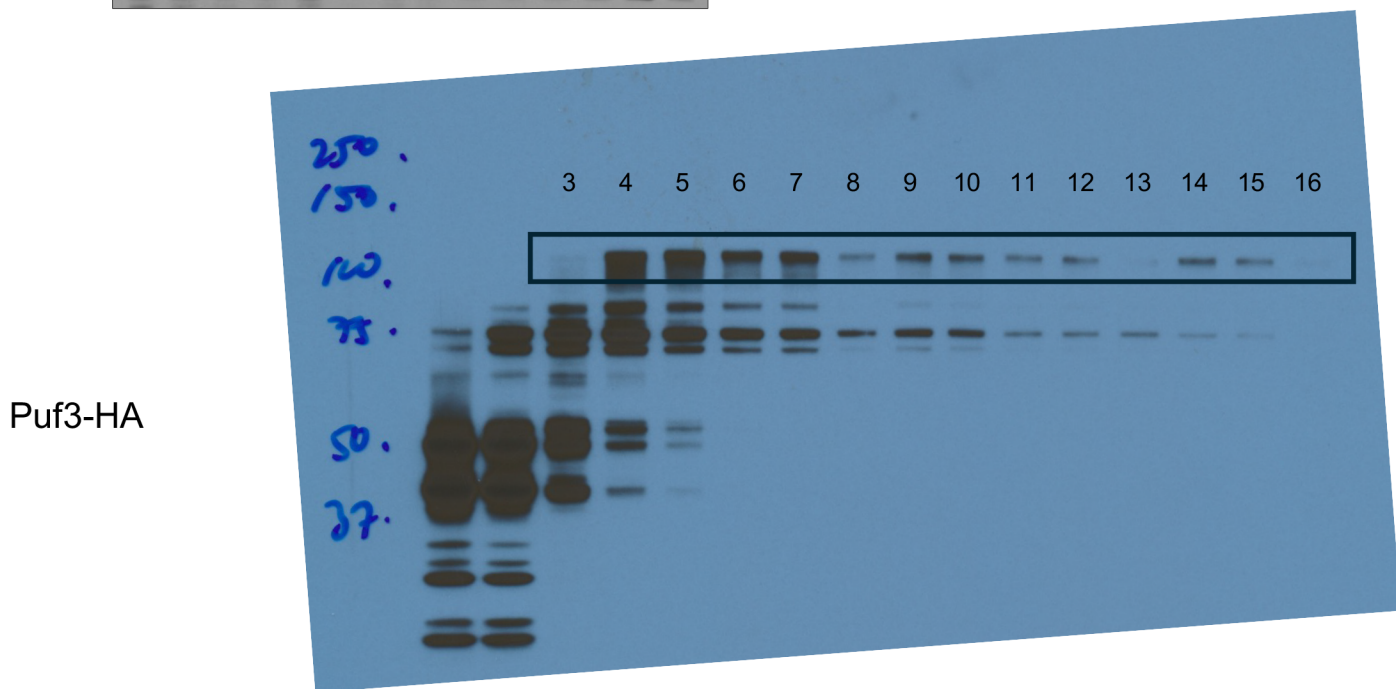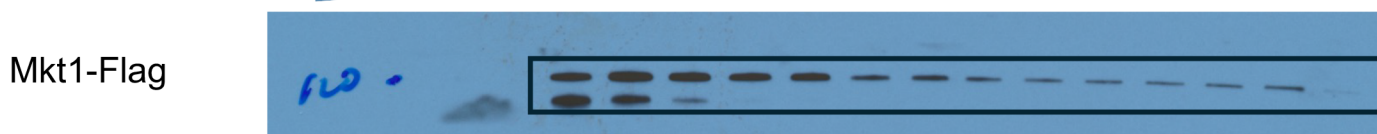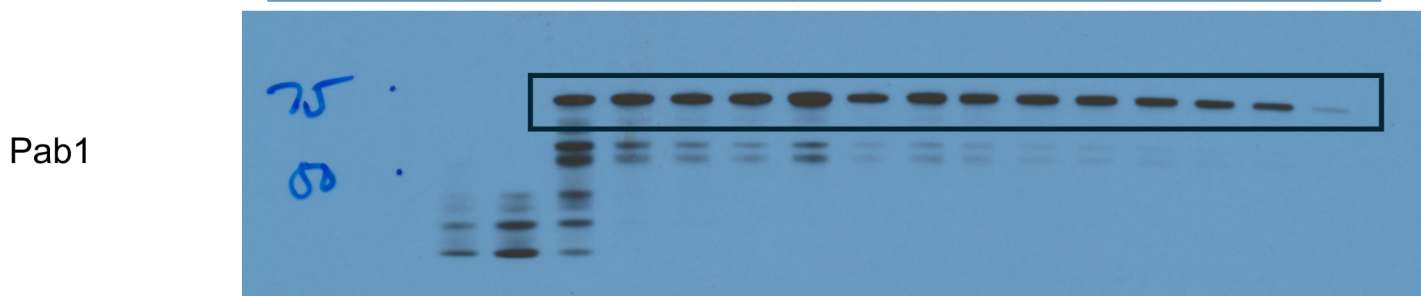

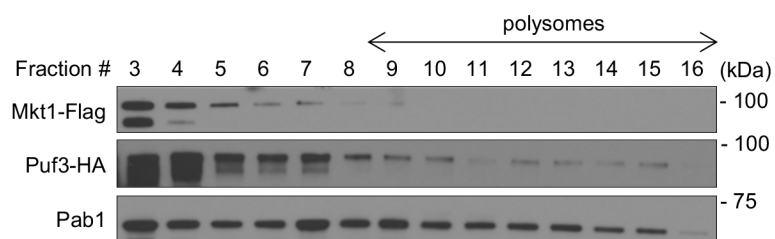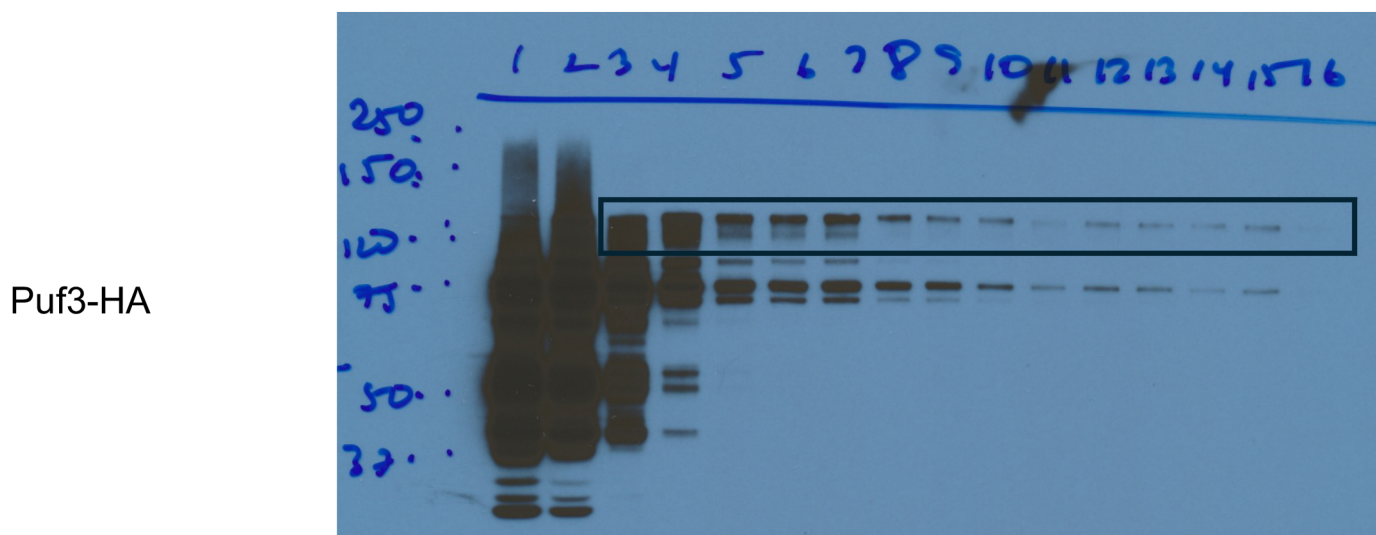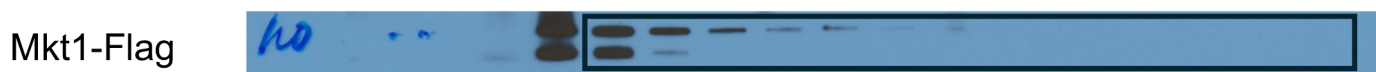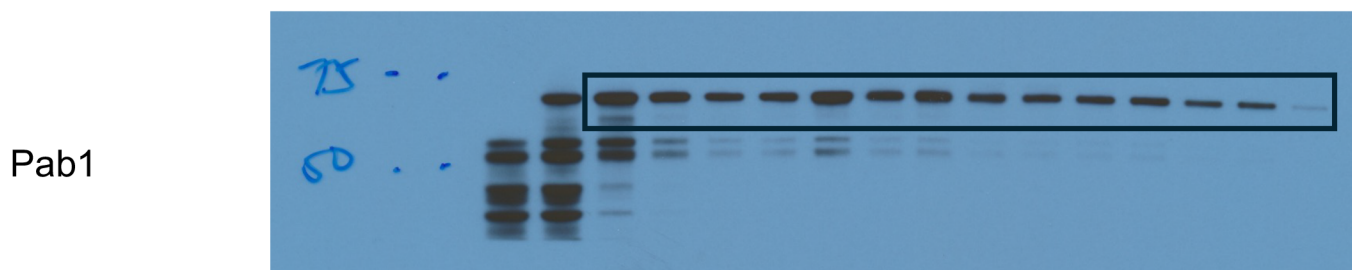

Figure 4E, Additional experiment

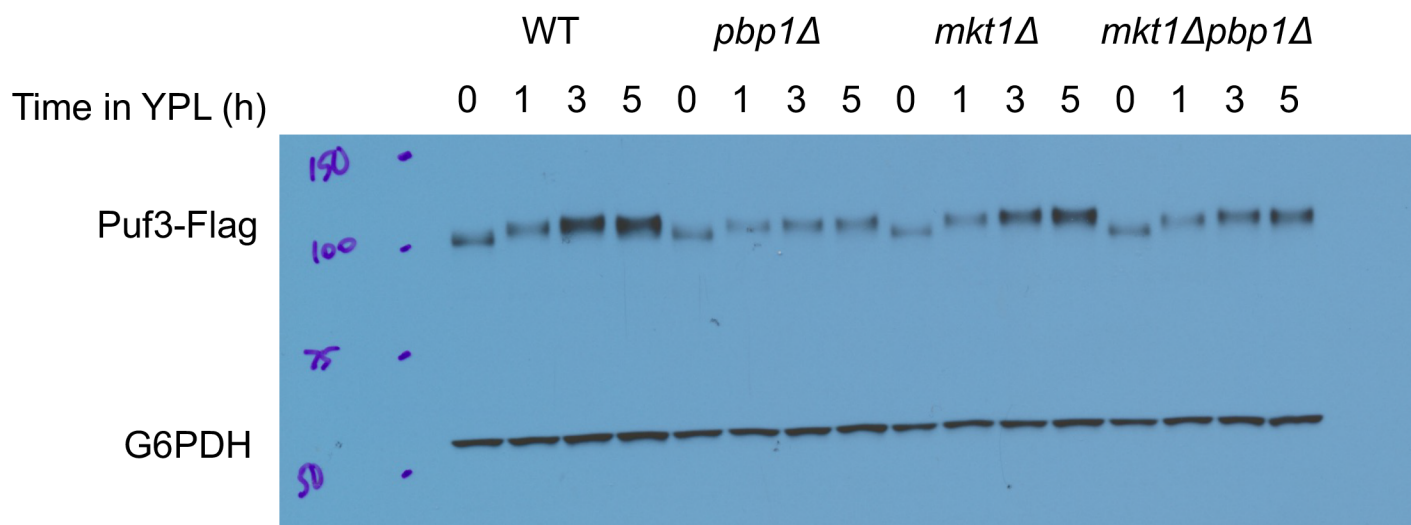

Figure 4F, Additional experiment

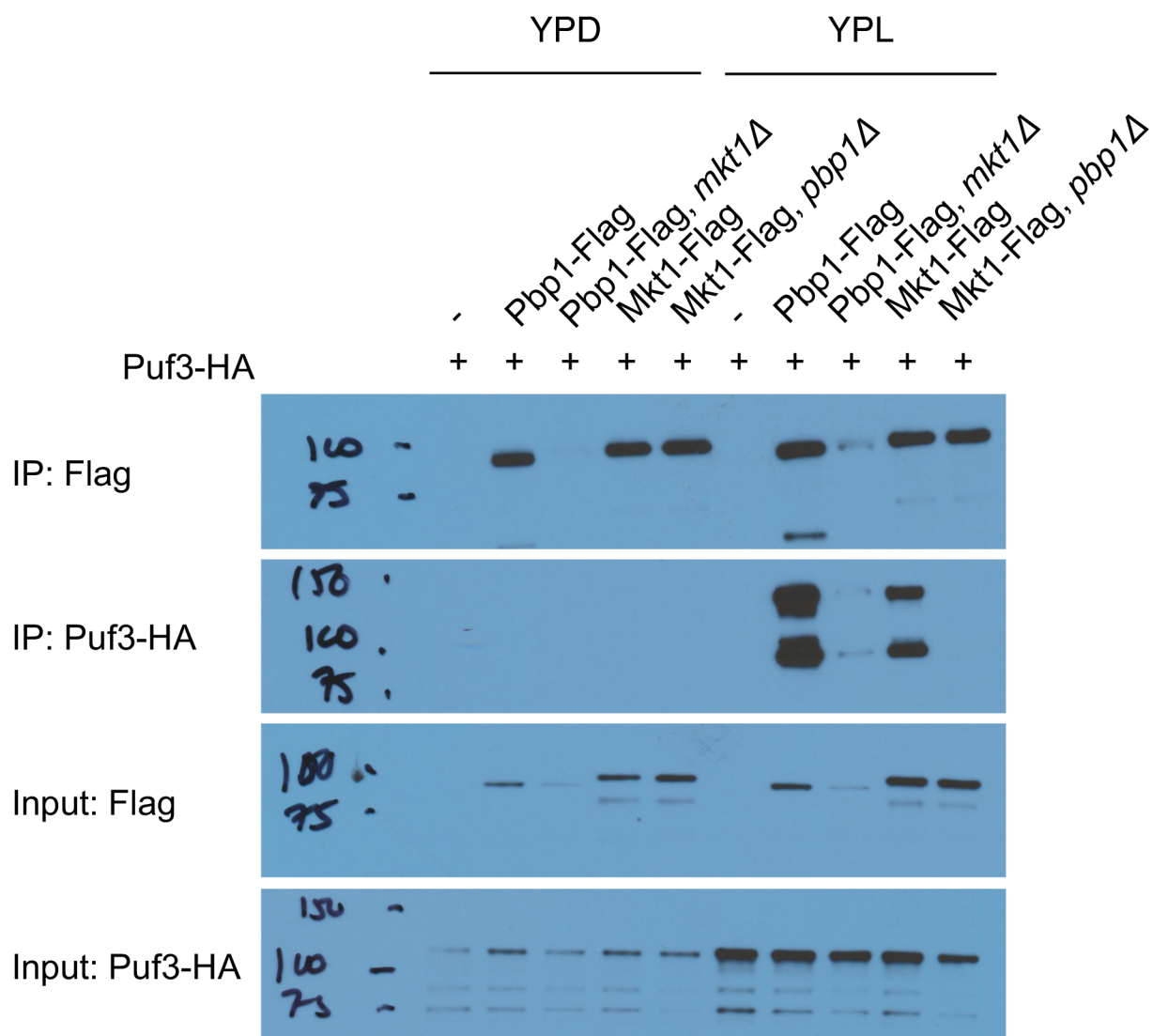

Supplement: SourceData F4 — is the source file for Fig. 4. [file jcb_202411169_sourcedataf4.pdf]

B

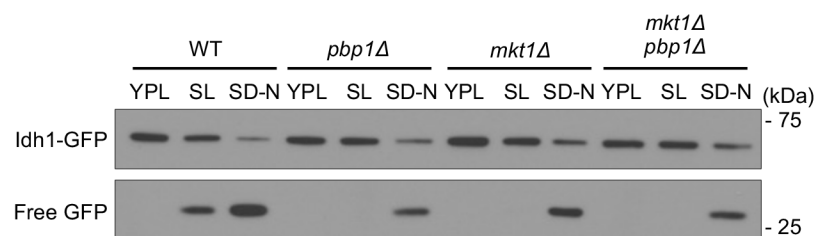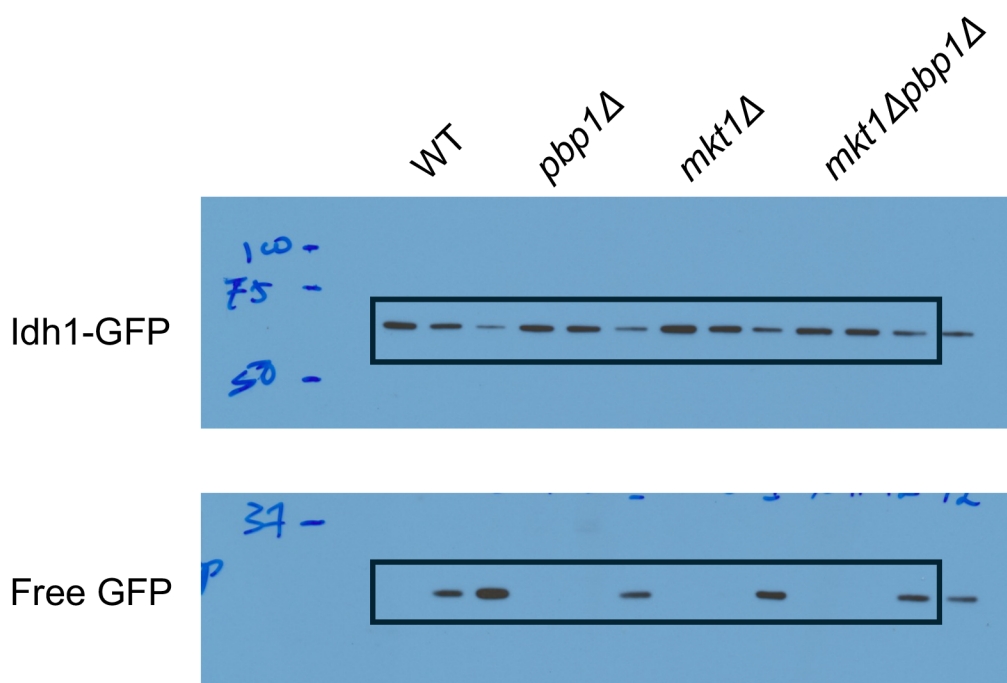

C

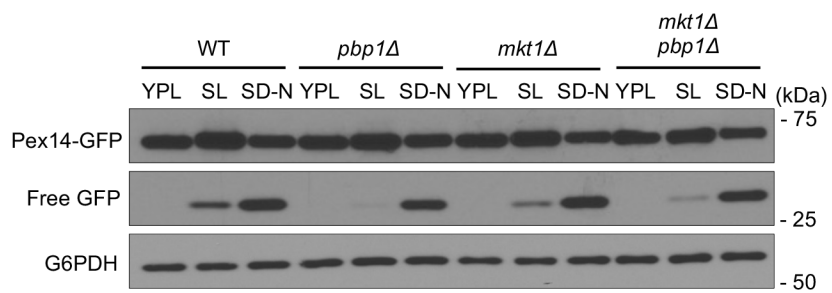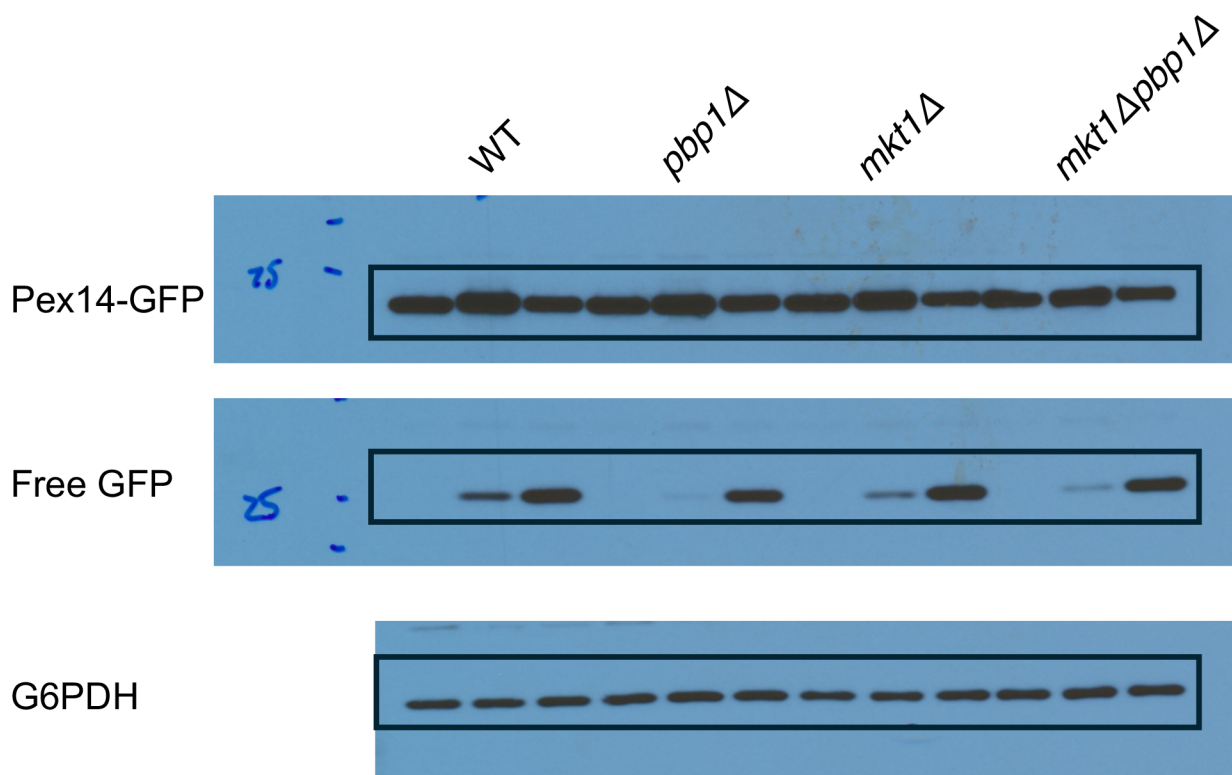

F

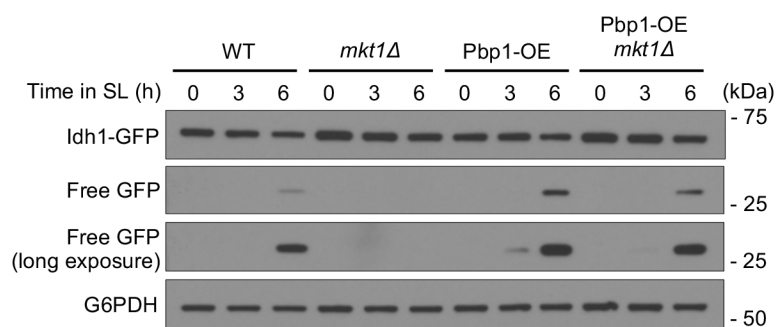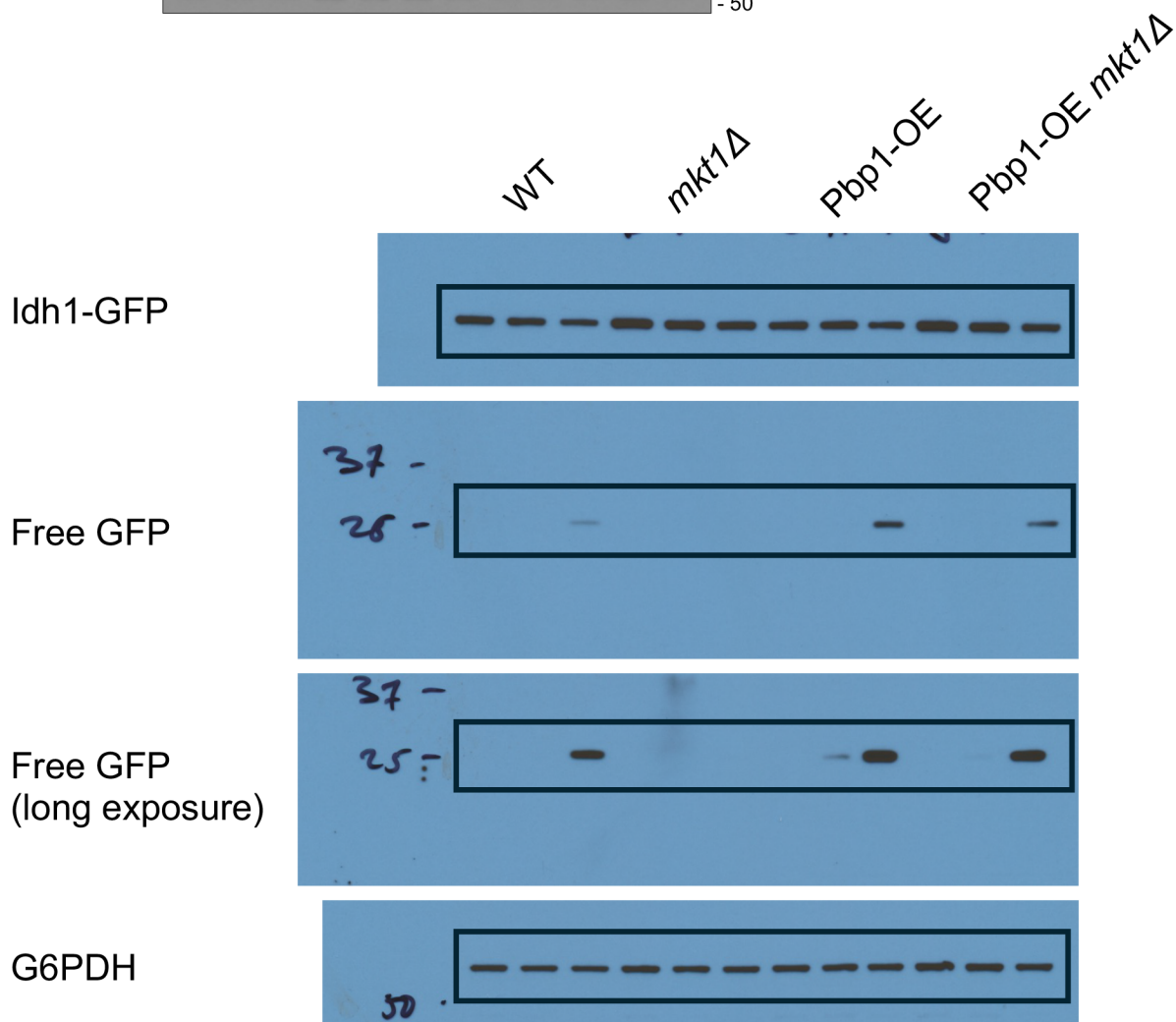

G

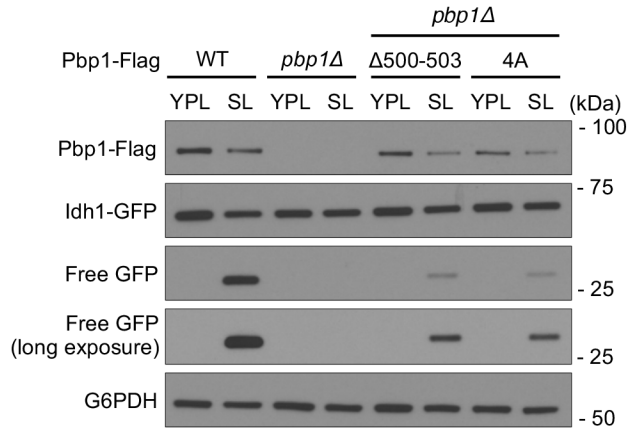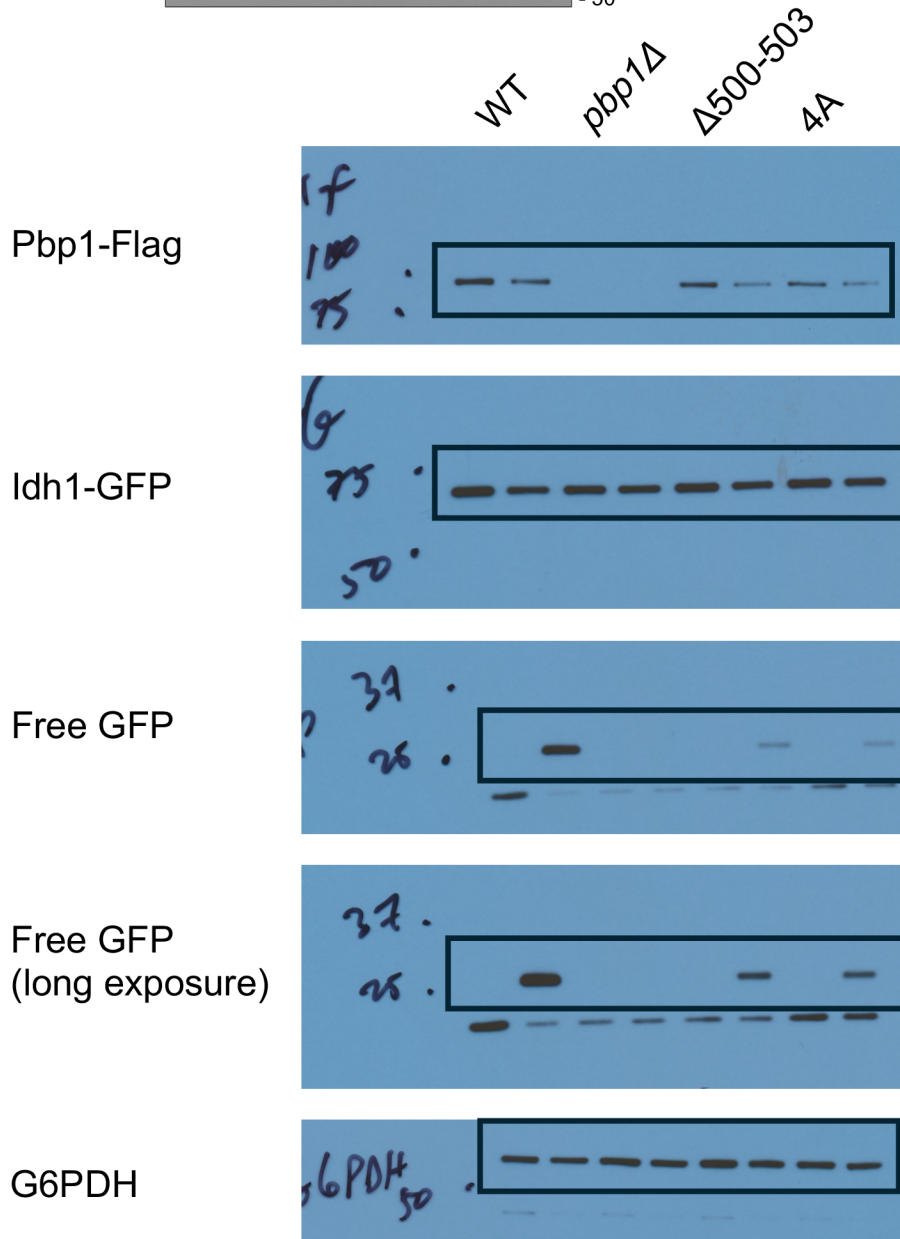

Figure 5B, Additional experiment

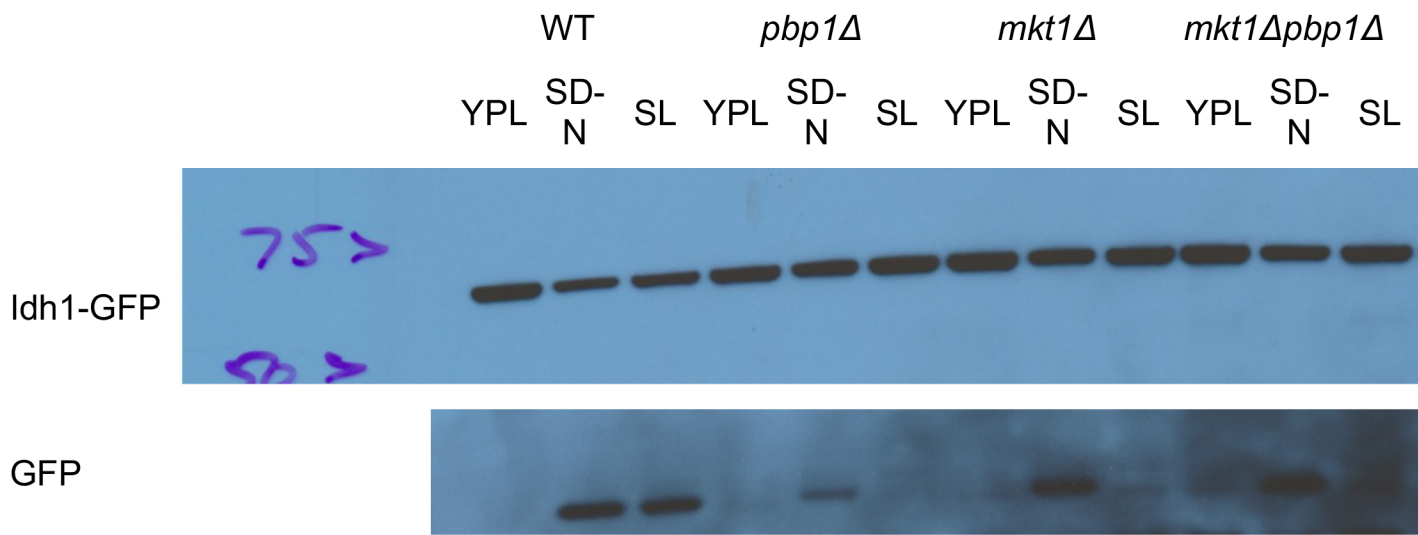

Supplement: SourceData F5 — is the source file for Fig. 5. [file jcb_202411169_sourcedataf5.pdf]

B

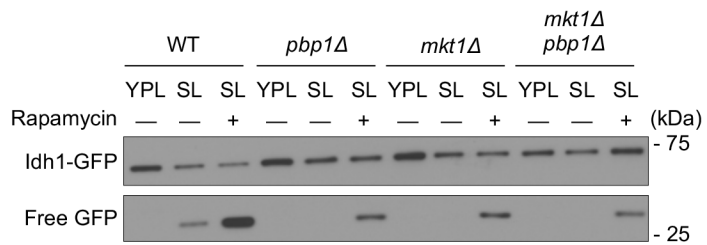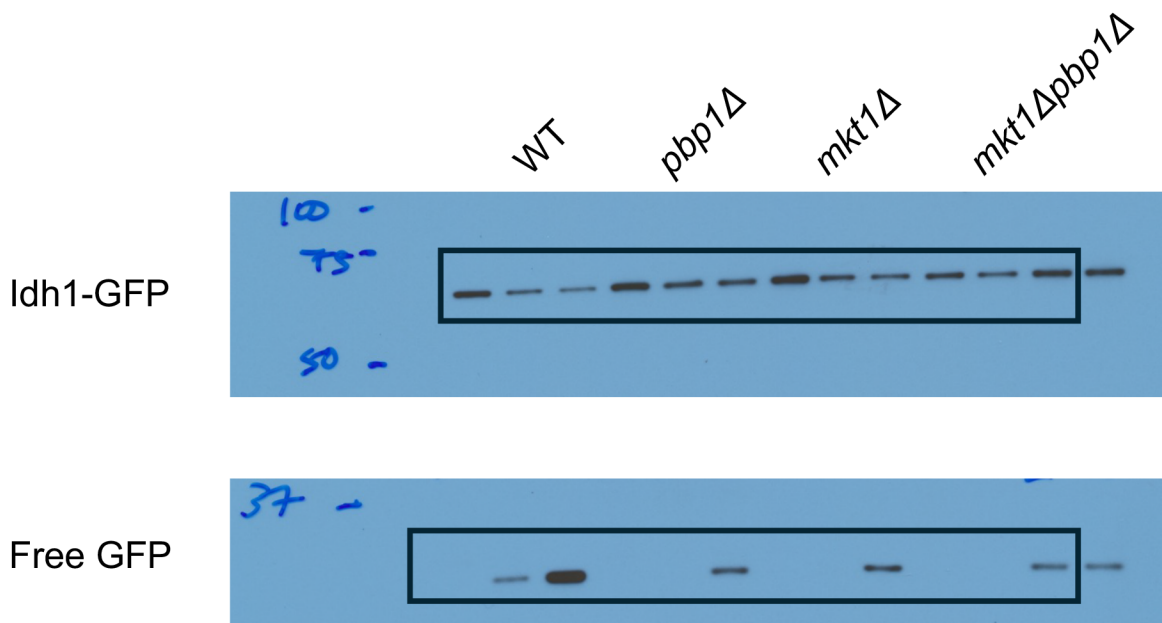

F

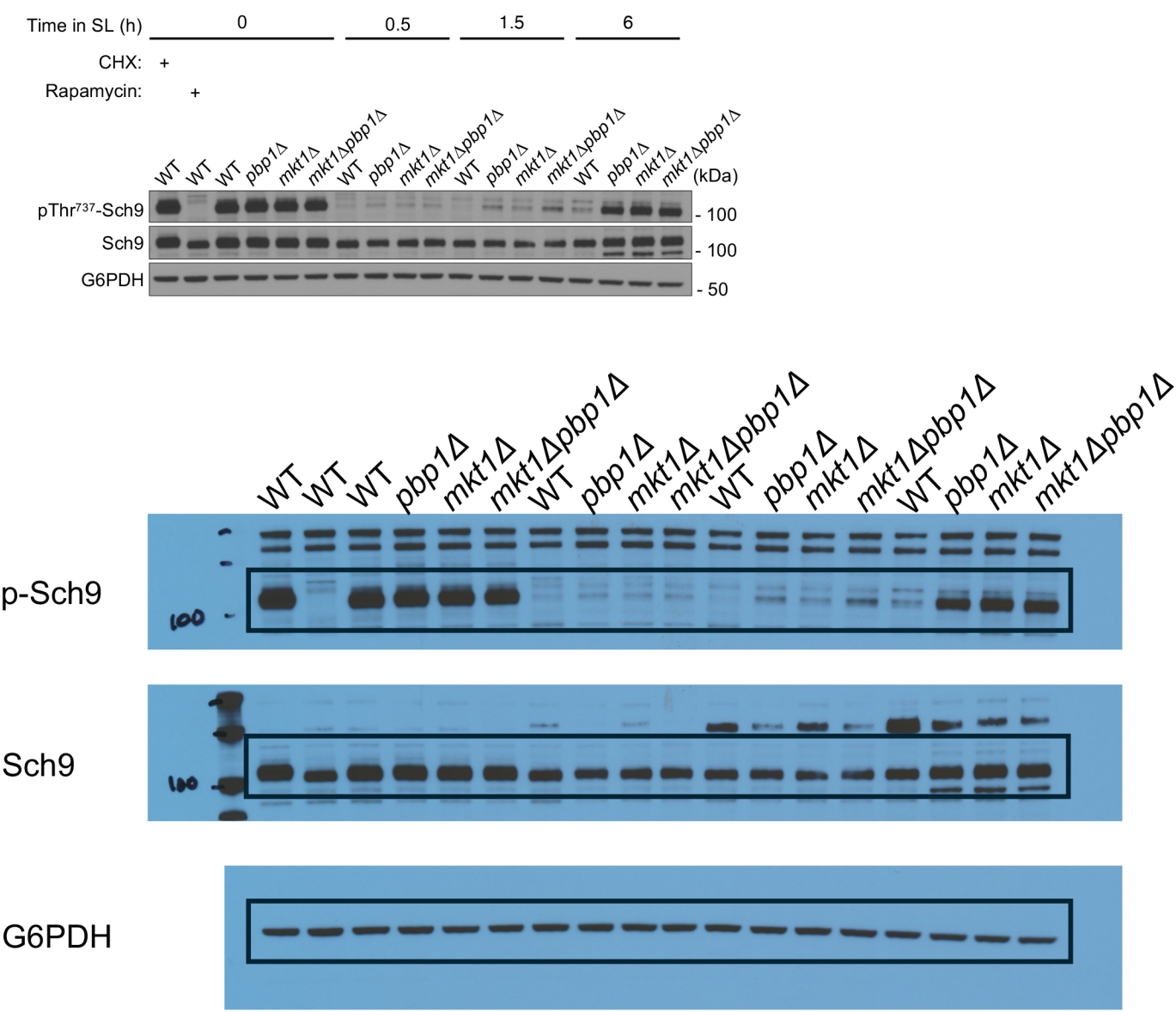

G

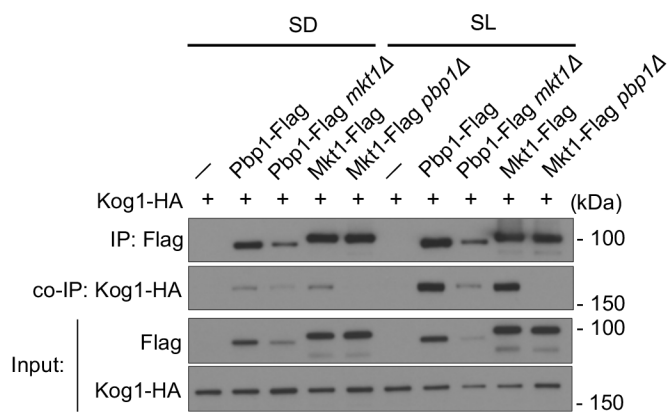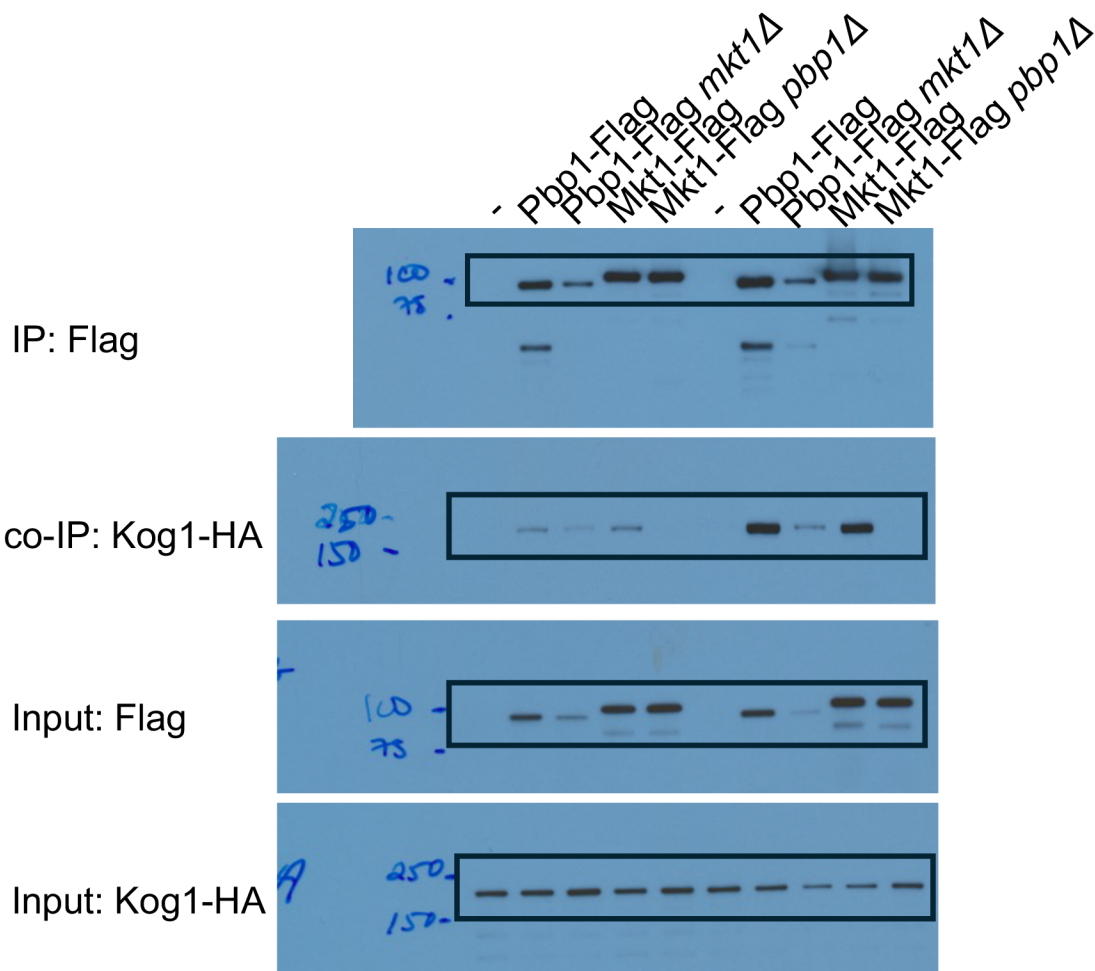

Figure 6G, Additional experiment

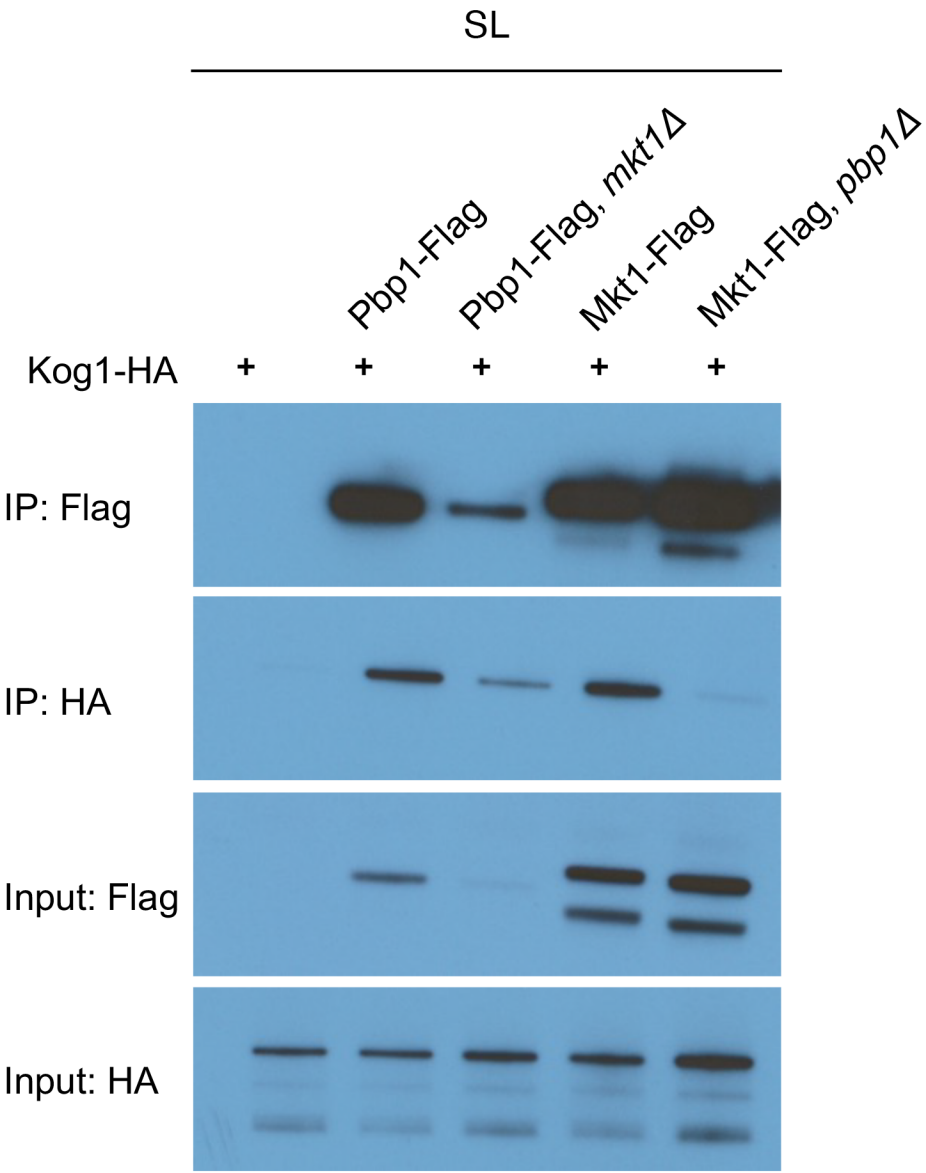

Supplement: SourceData F6 — is the source file for Fig. 6. [file jcb_202411169_sourcedataf6.pdf]

B

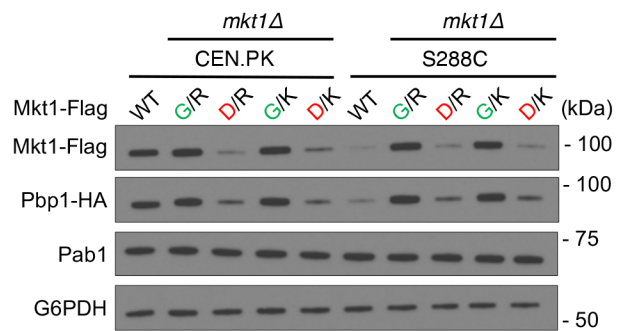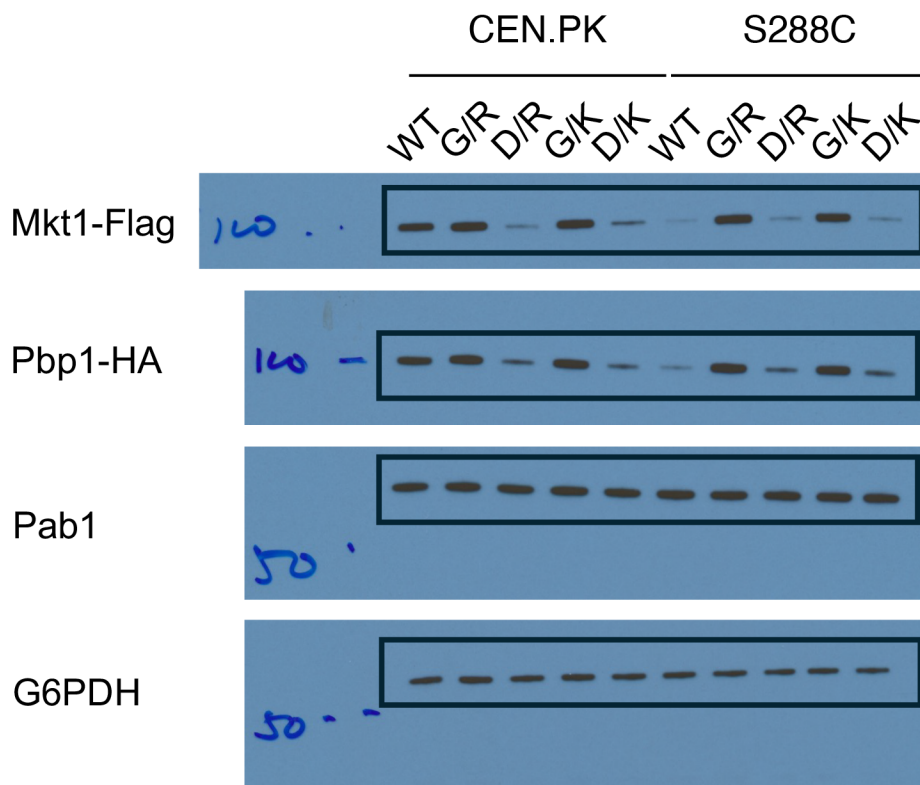

C

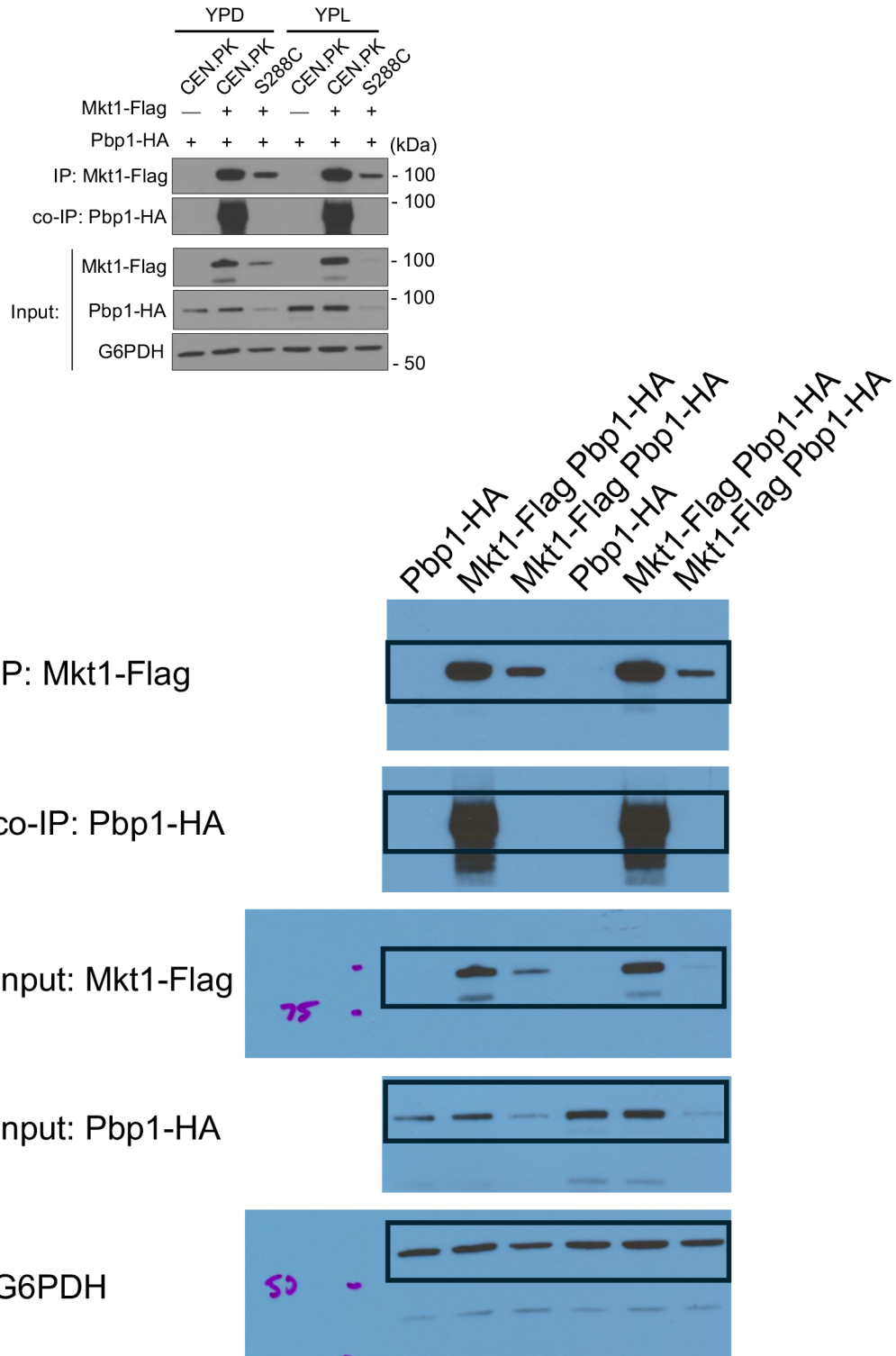

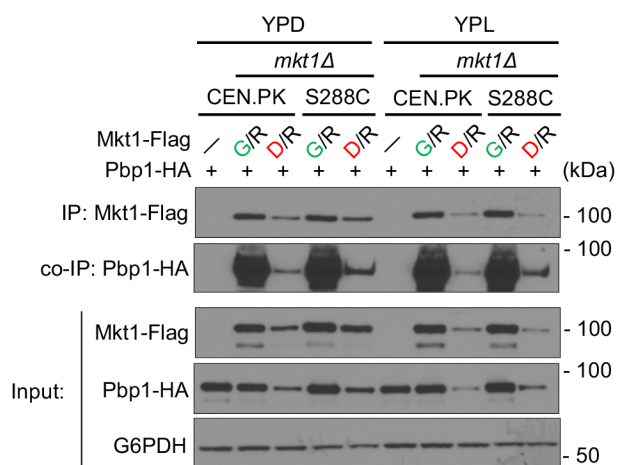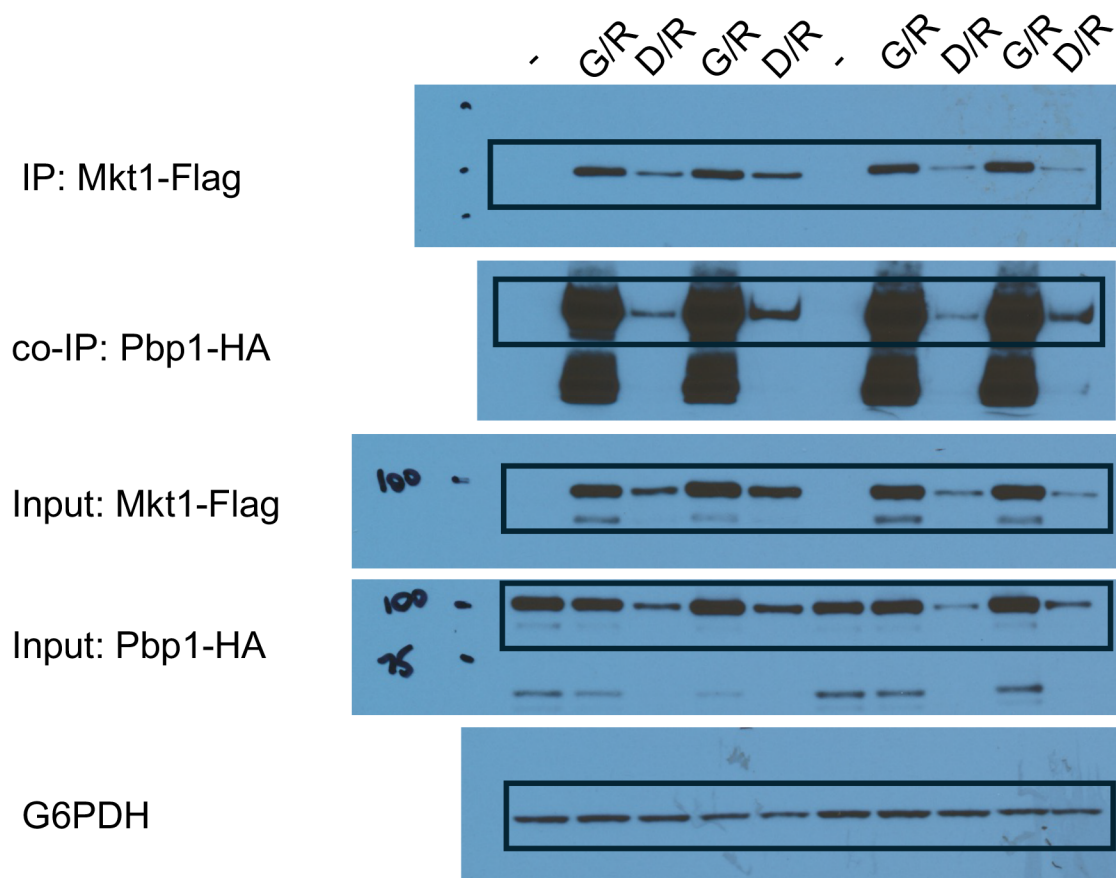

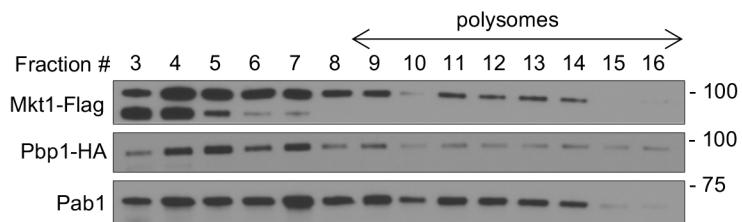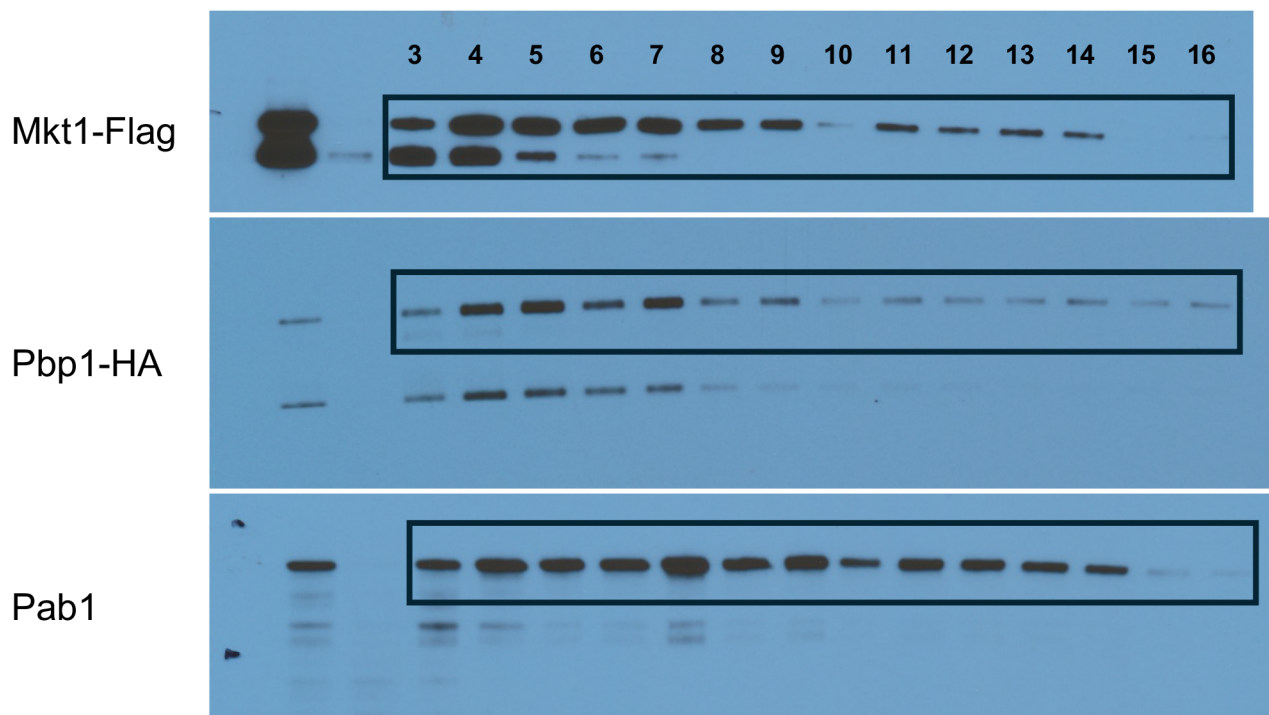

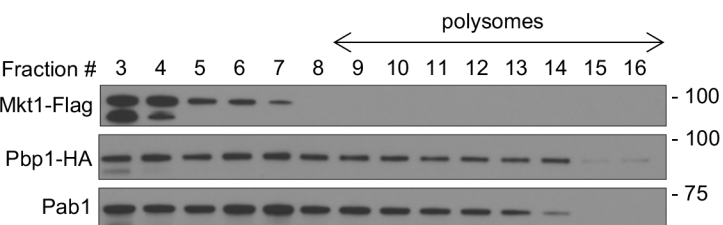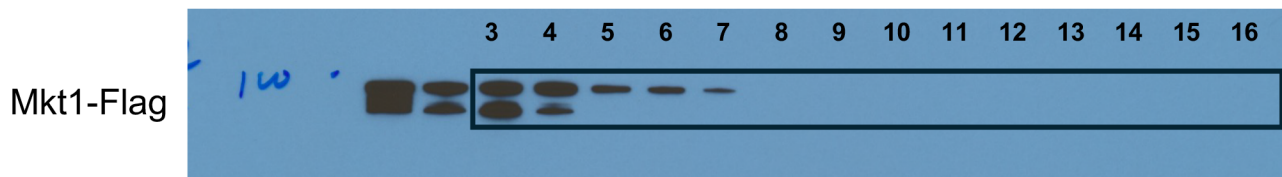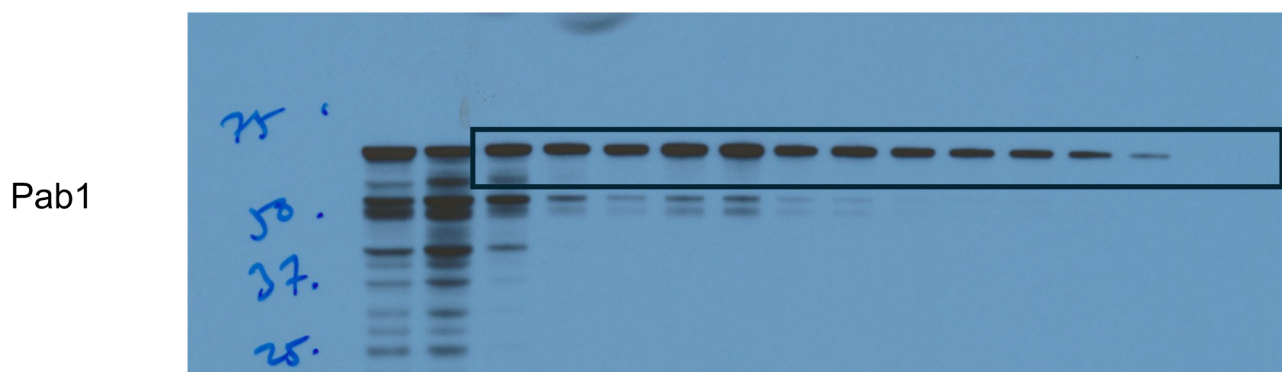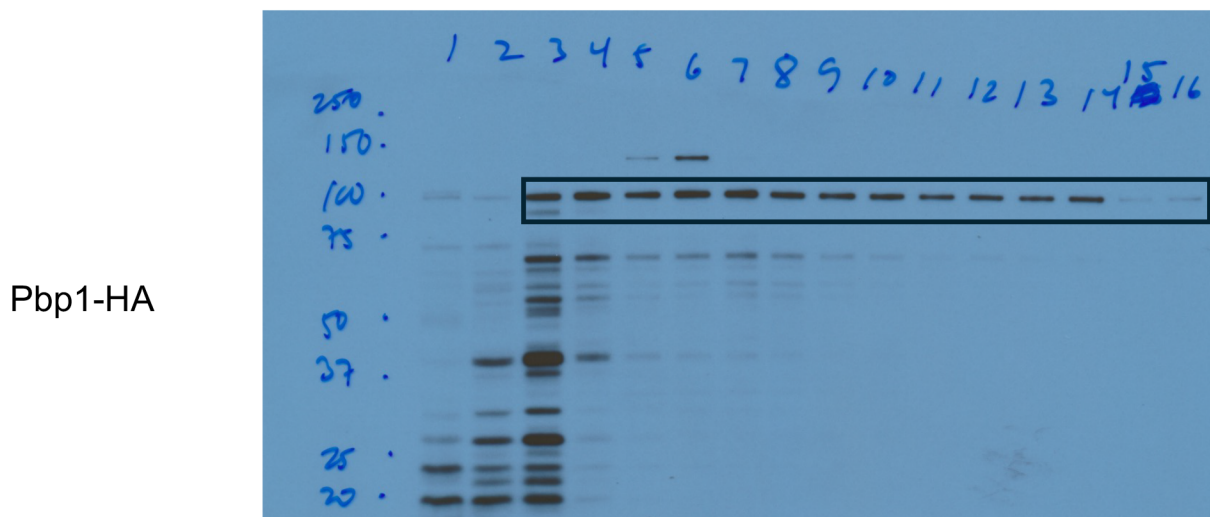

Figure 7B, Additional experiment

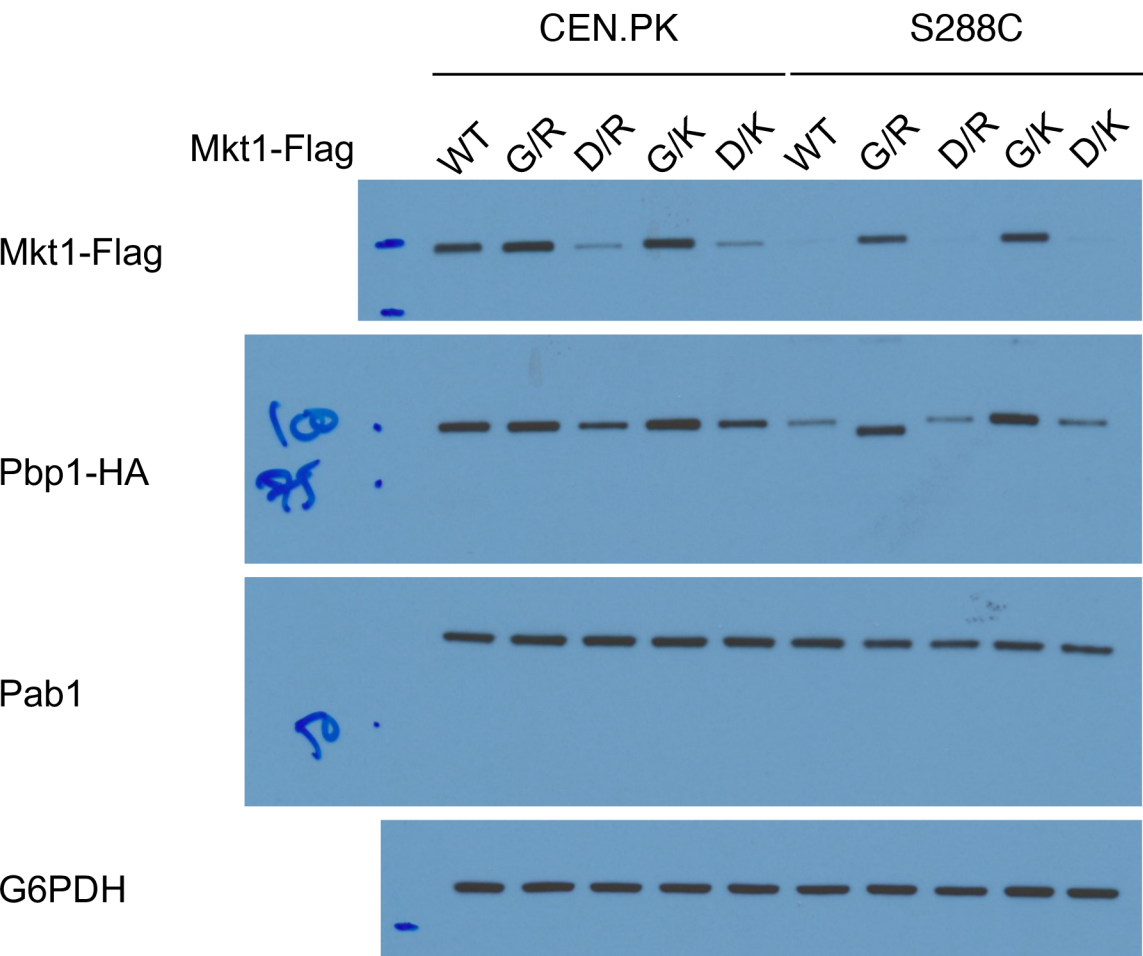

Supplement: SourceData F7 — is the source file for Fig. 7. [file jcb_202411169_sourcedataf7.pdf]

C

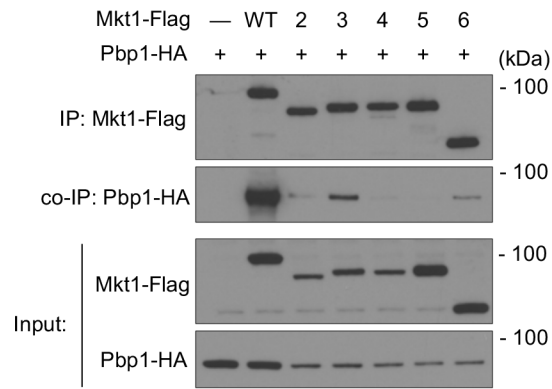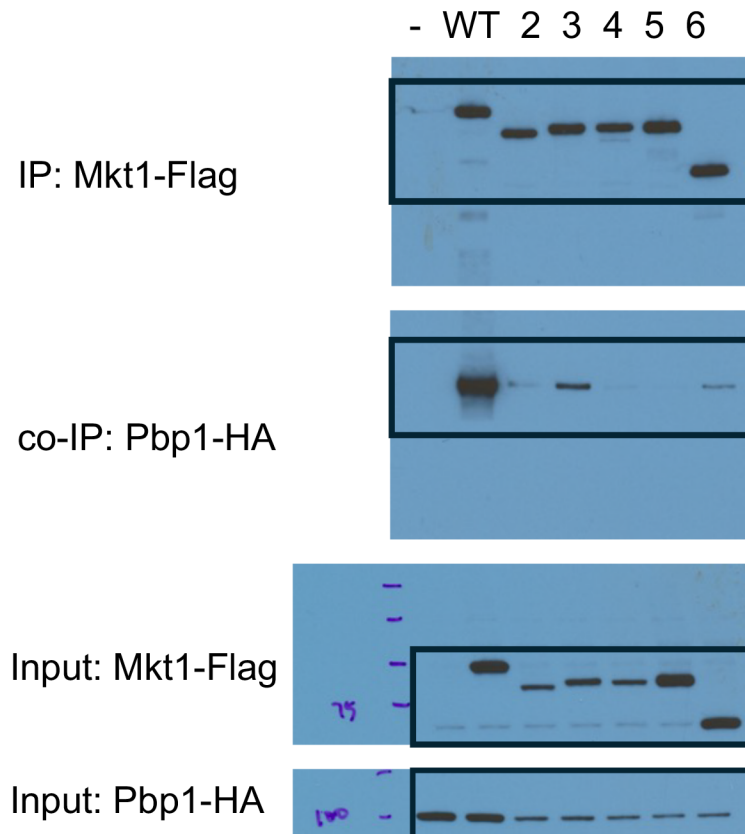

Figure S2C, Additional experiment

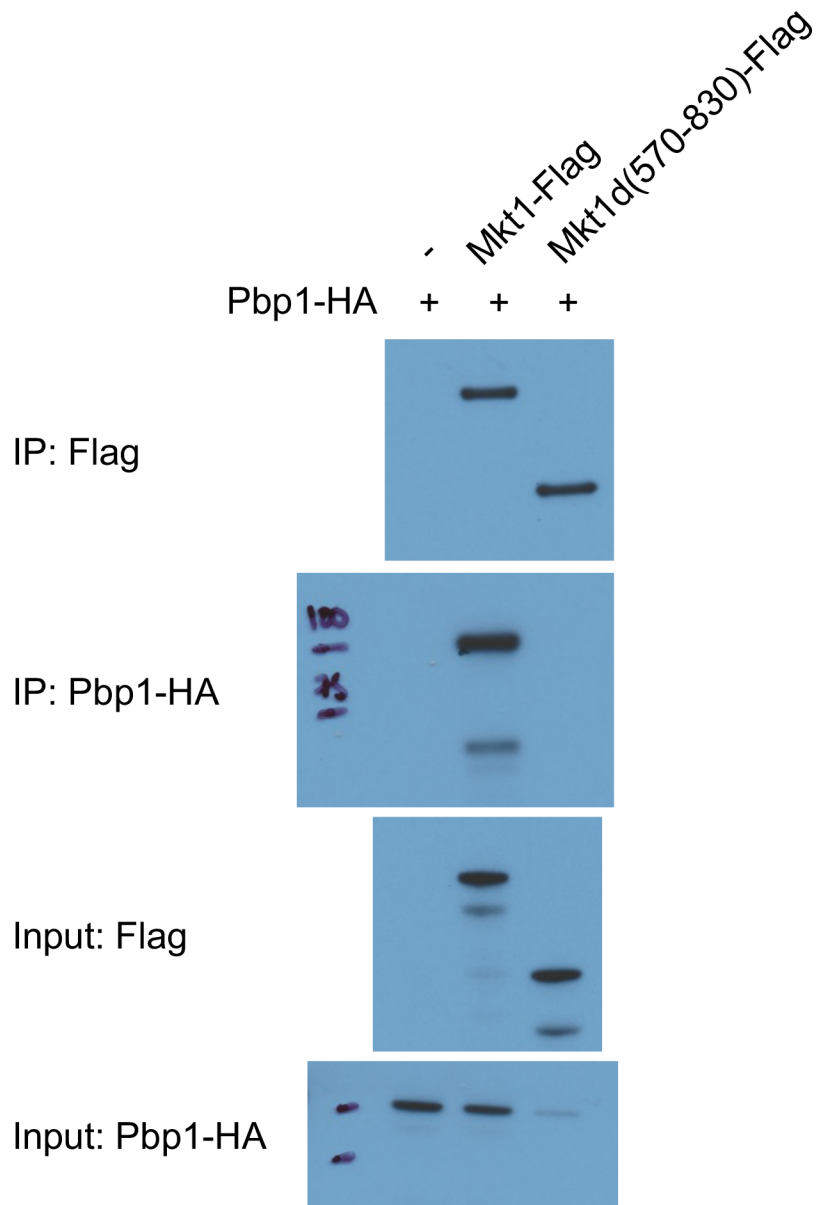

Supplement: SourceData FS2 — is the source file for Fig. S2. [file jcb_202411169_sourcedatafs2.pdf]

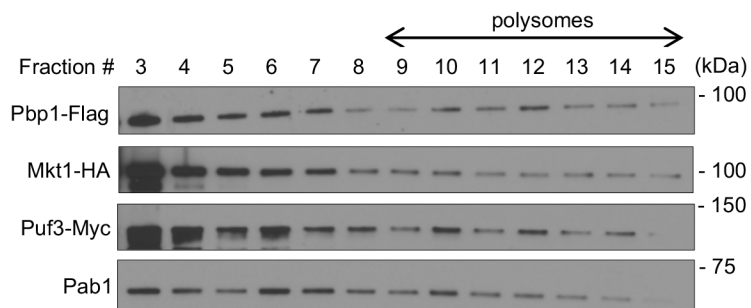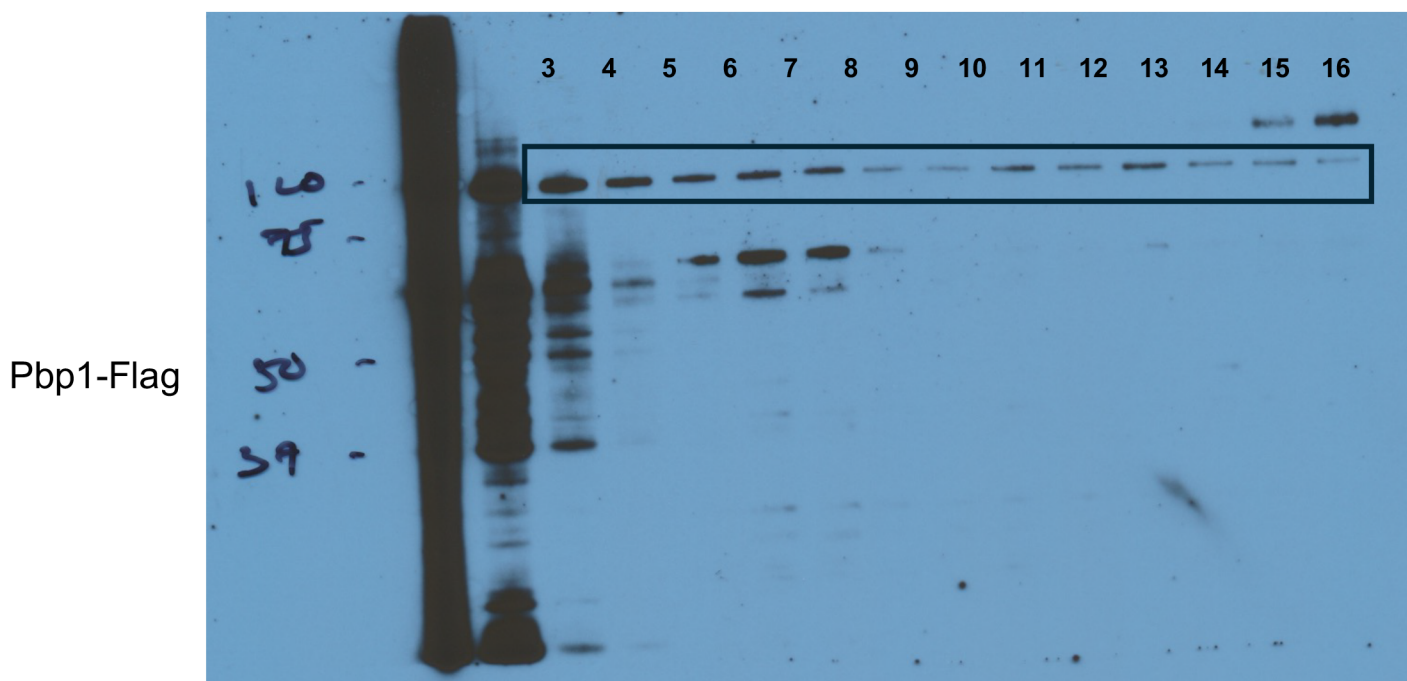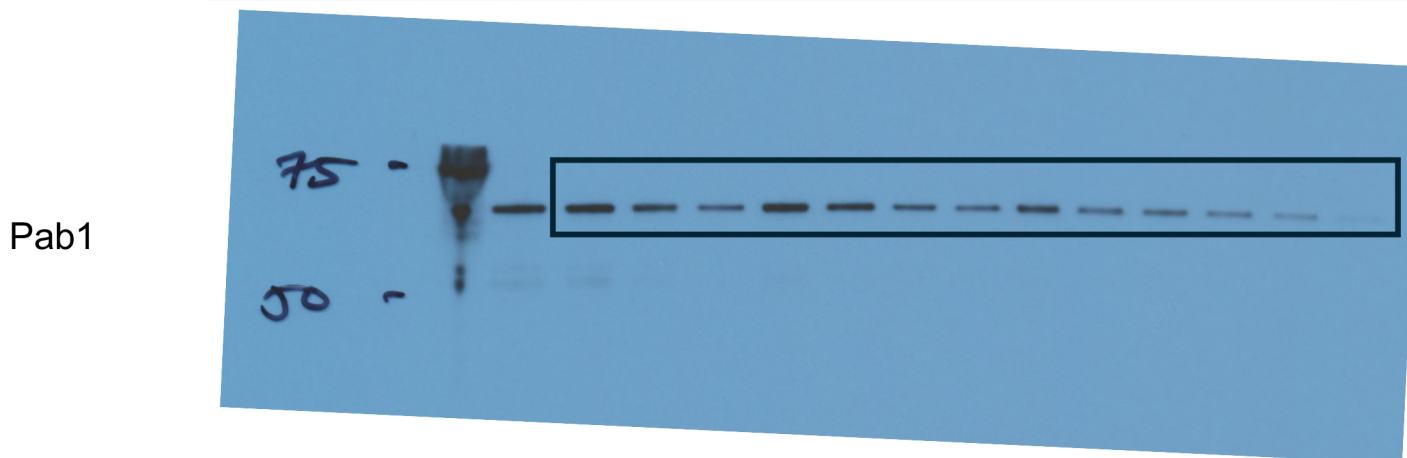

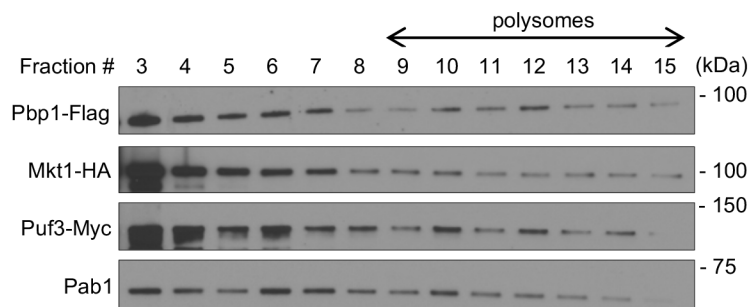

Mkt1-HA

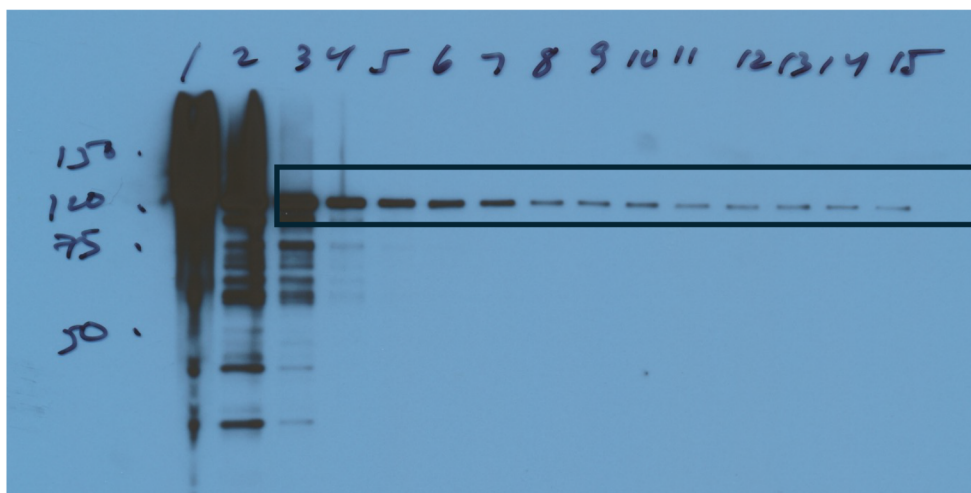

Puf3-myc

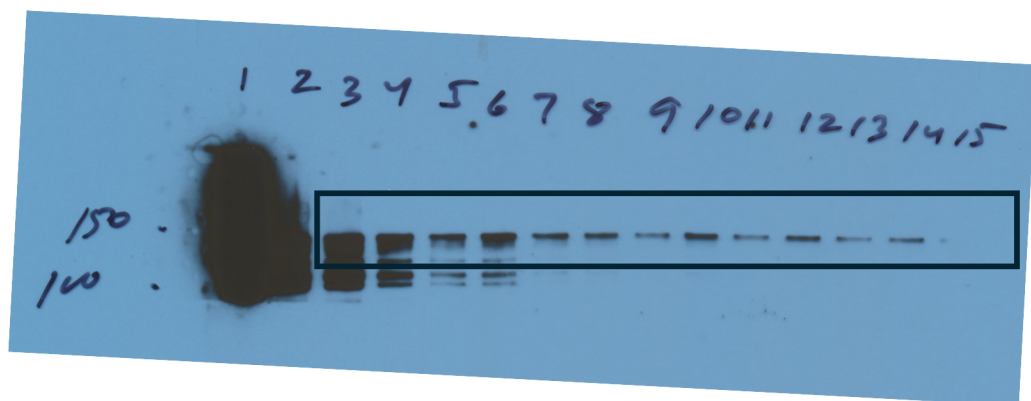



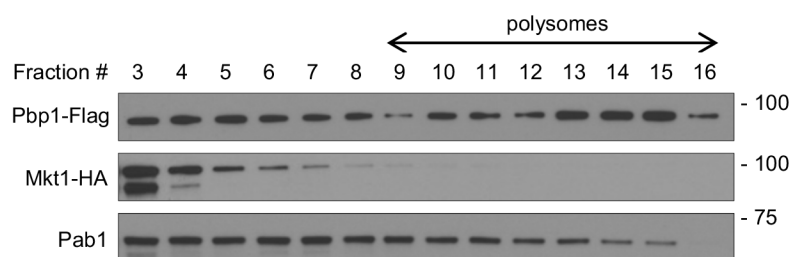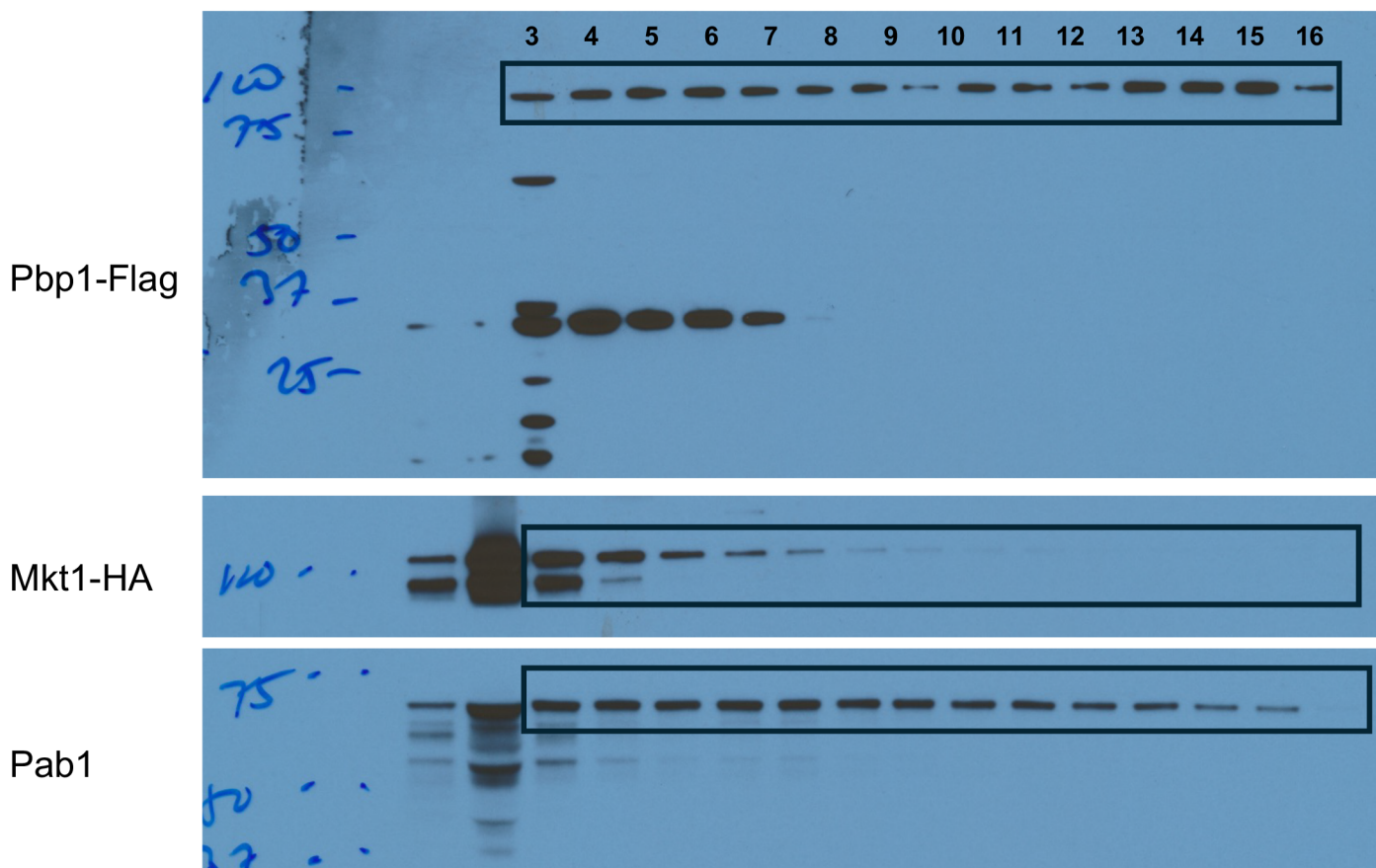

Supplement: SourceData FS4 — is the source file for Fig. S4. [file jcb_202411169_sourcedatafs4.pdf]

B

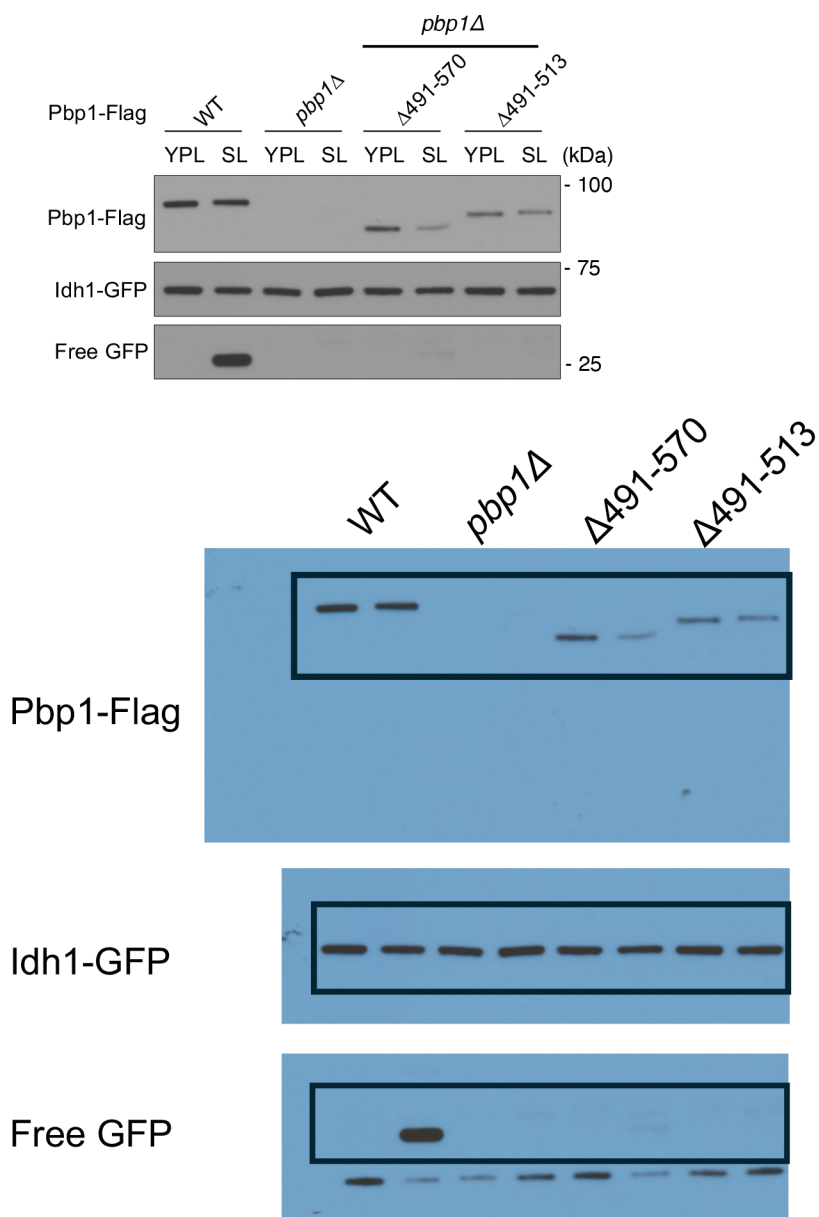

C

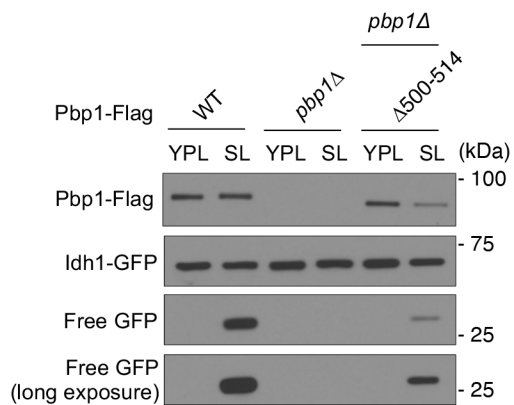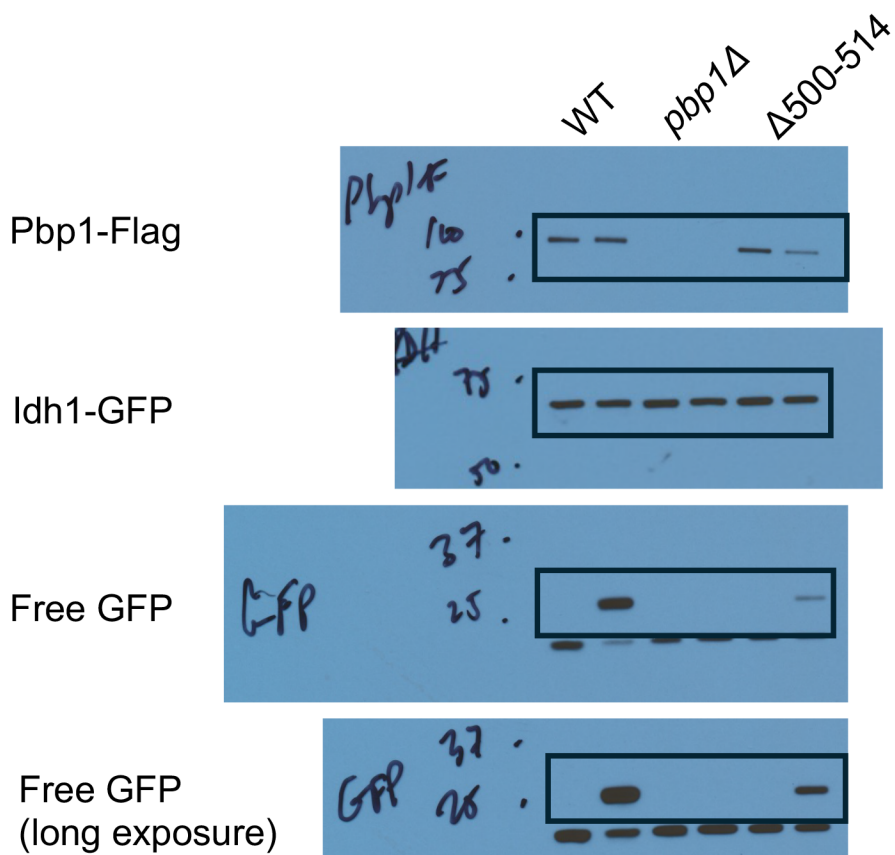

D

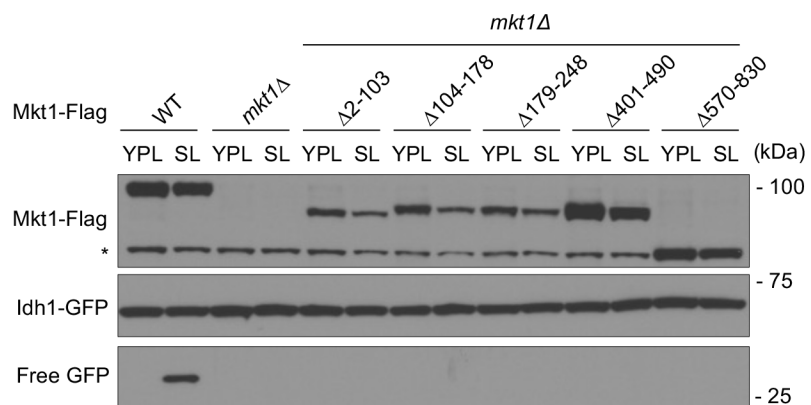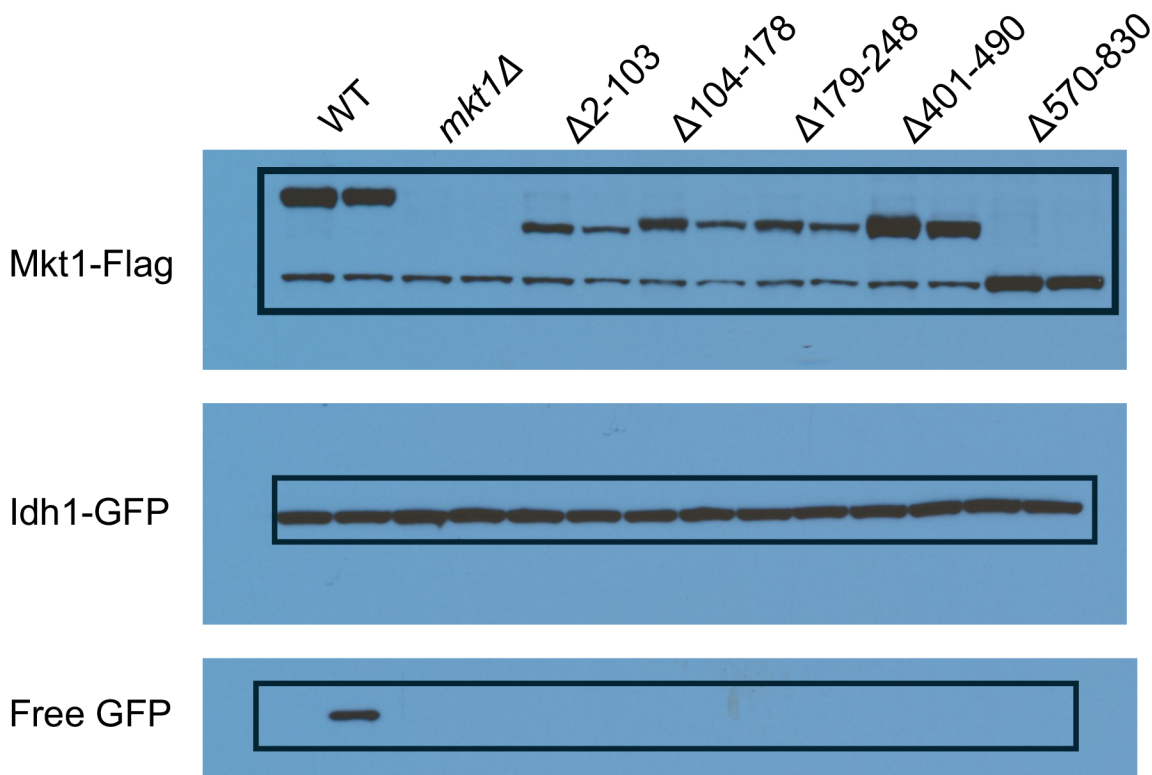

Figure S5D, Additional experiment

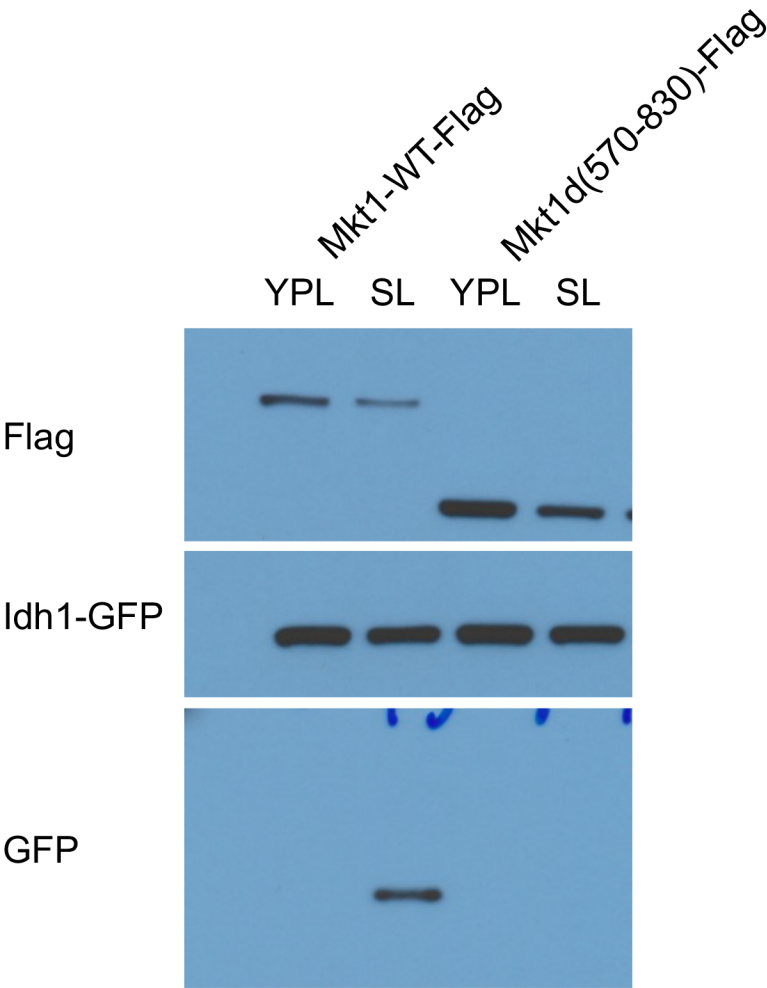

Supplement: SourceData FS5 — is the source file for Fig. S5. [file jcb_202411169_sourcedatafs5.pdf]
