# Supplementary material for: The yeast Mkt1/Pbp1 complex promotes adaptive responses to respiratory growth
Source: J Cell Biol. 2025 Aug 13;224(10):e202411169. doi: 10.1083/jcb.202411169 (PMC12345631; doi:10.1083/jcb.202411169)

C

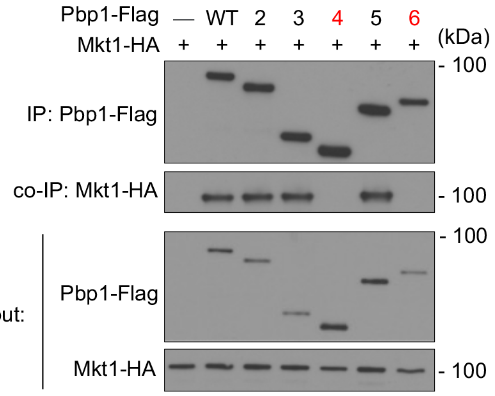
Pbp1-Flag — WT 2 3 4 5 6
Mkt1-HA + + + + + + +
(kDa)
IP: Pbp1-Flag
- 100
co-IP: Mkt1-HA
- 100
Input:
Pbp1-Flag
- 100
Mkt1-HA
- 100

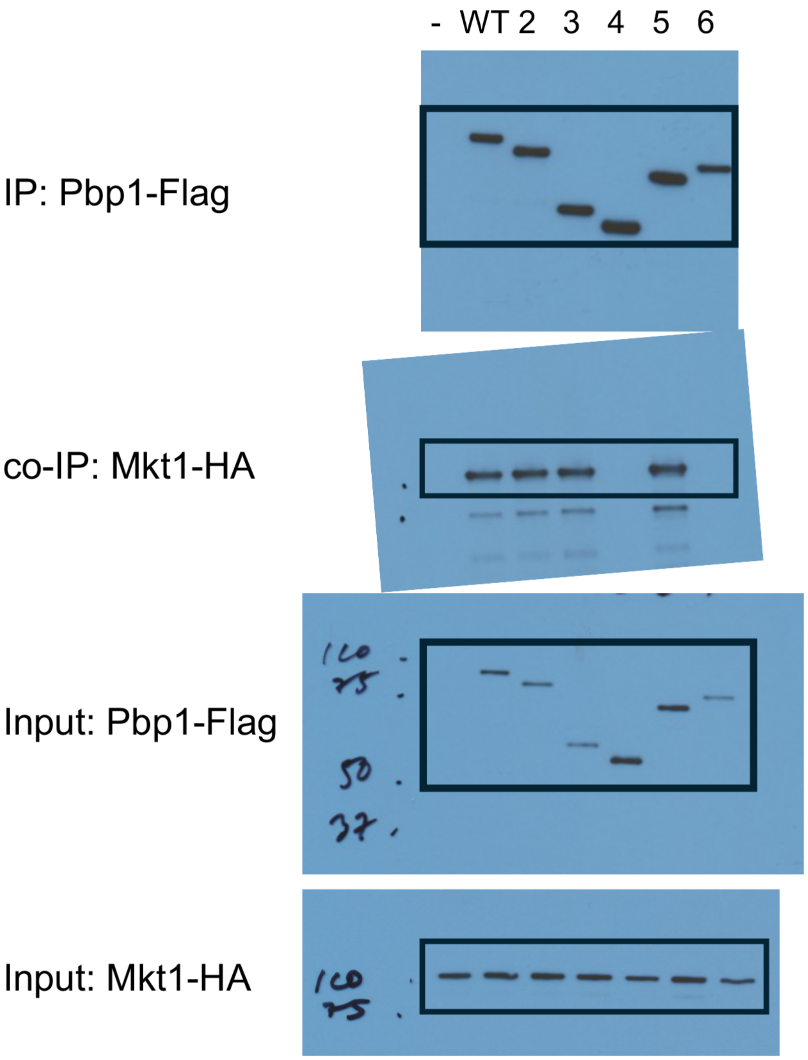
- WT 2 3 4 5 6
IP: Pbp1-Flag
co-IP: Mkt1-HA
Input: Pbp1-Flag
100
75
50
37
Input: Mkt1-HA
100
75

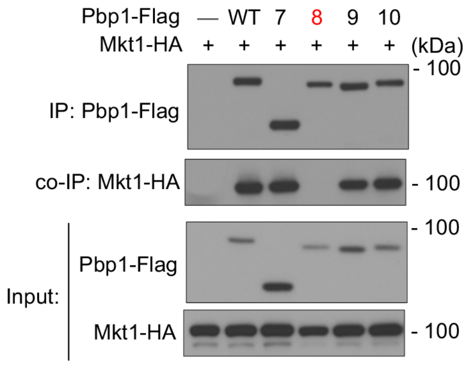
Pbp1-Flag — WT 7 8 9 10
Mkt1-HA + + + + + +
(kDa)
- 100
IP: Pbp1-Flag
co-IP: Mkt1-HA
- 100
- 100
Pbp1-Flag
Input:
Mkt1-HA
- 100

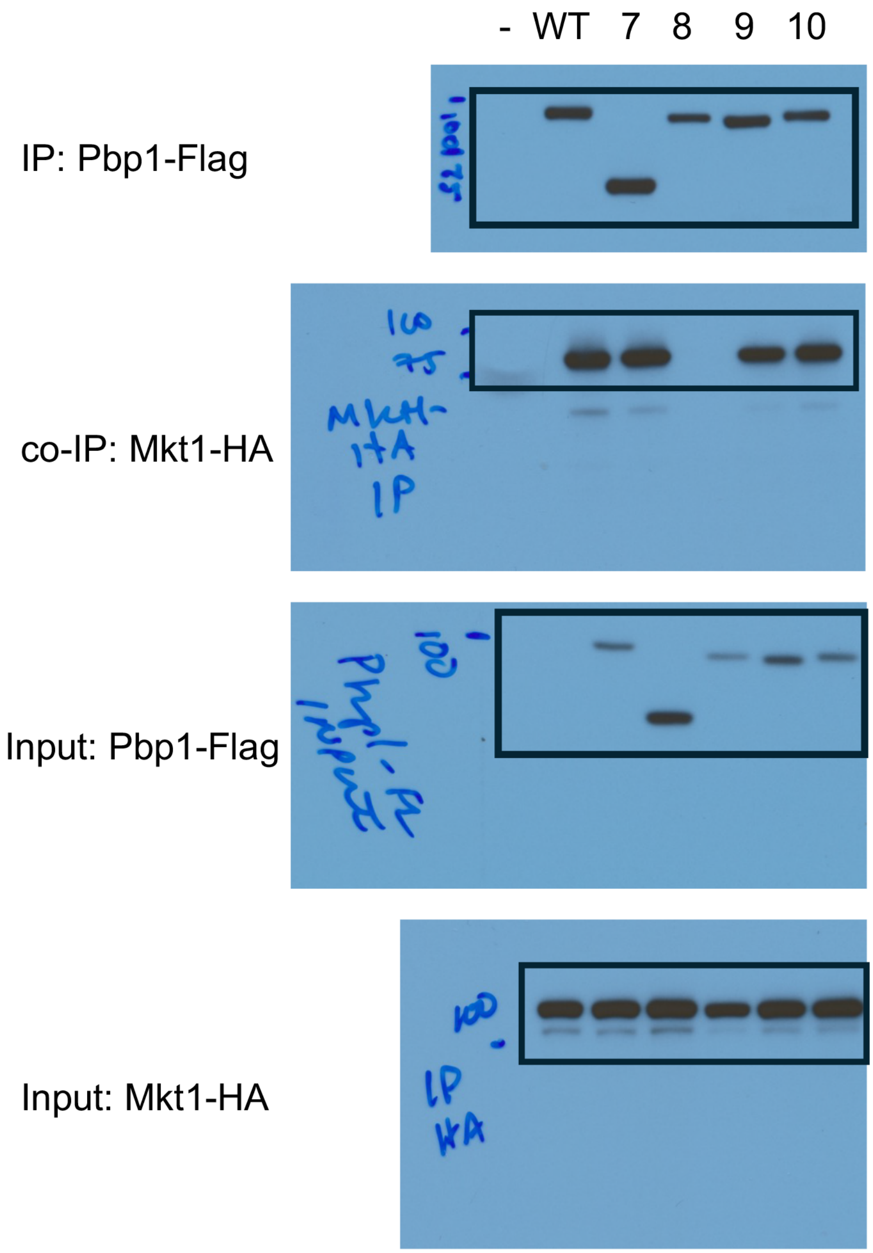
- WT 7 8 9 10
IP: Pbp1-Flag
co-IP: Mkt1-HA
Input: Pbp1-Flag
Input: Mkt1-HA

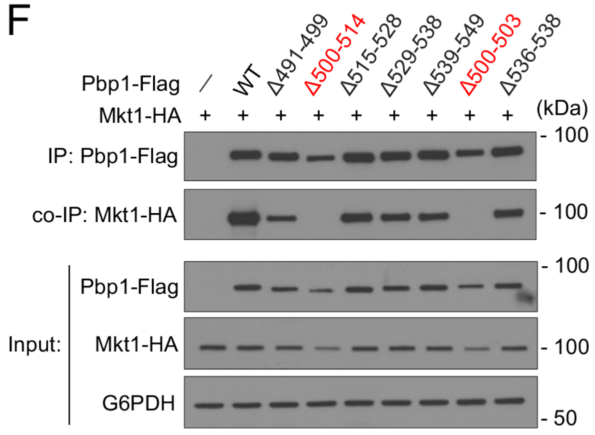
F
Pbp1-Flag
/
WT
Δ491-499
Δ500-514
Δ515-528
Δ529-538
Δ539-549
Δ500-503
Δ536-538
Mkt1-HA
+ + + + + + + + +
(kDa)
IP: Pbp1-Flag
- 100
co-IP: Mkt1-HA
- 100
Pbp1-Flag
- 100
Input:
Mkt1-HA
- 100
G6PDH
- 50

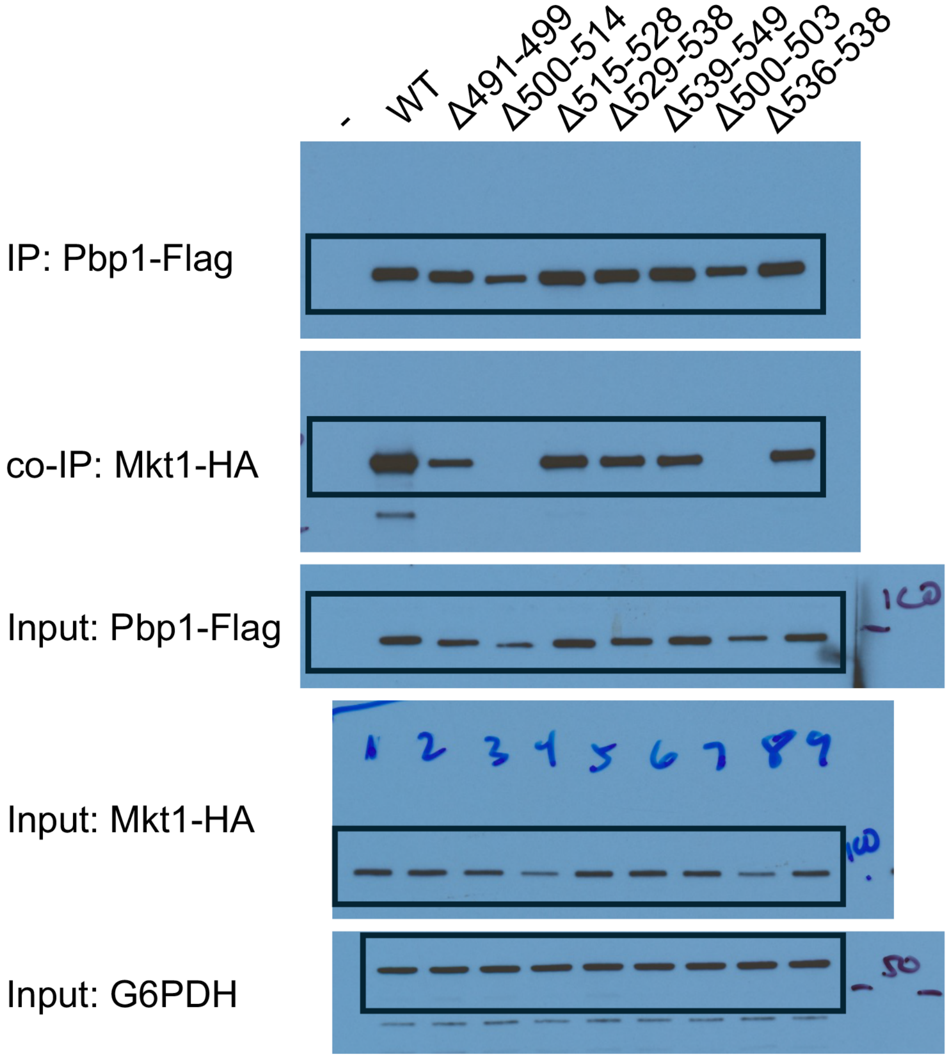
-
WT
Δ491-499
Δ500-514
Δ515-528
Δ529-538
Δ539-549
Δ500-503
Δ536-538
IP: Pbp1-Flag
co-IP: Mkt1-HA
Input: Pbp1-Flag
100
1 2 3 4 5 6 7 8 9
Input: Mkt1-HA
100
Input: G6PDH
50

G

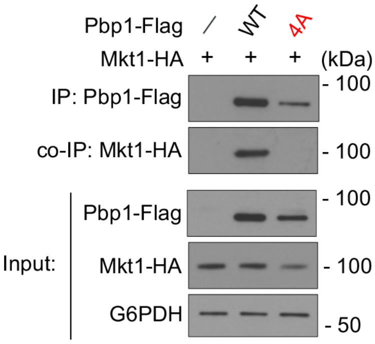


IP: Pbp1-Flag

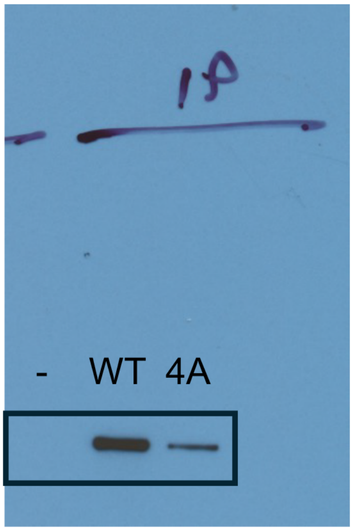


co-IP: Mkt1-HA

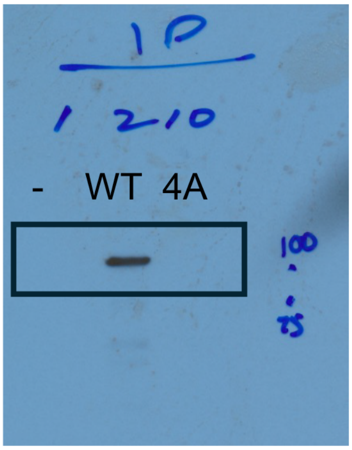


Input:
Pbp1-Flag

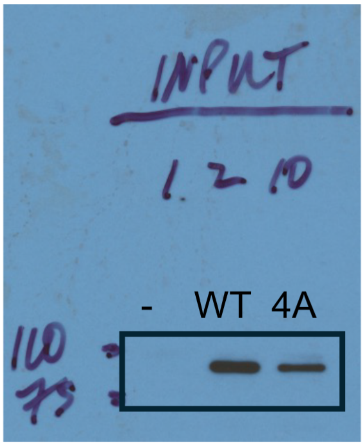


Input:
Mkt1-HA

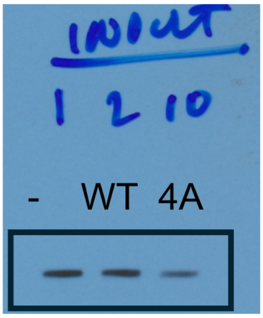


Input:
G6PDH

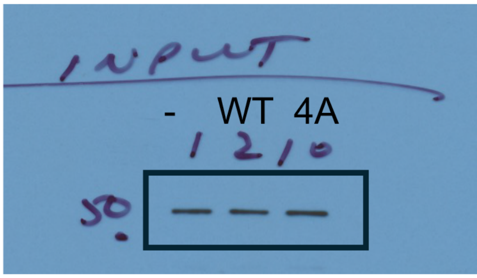

I

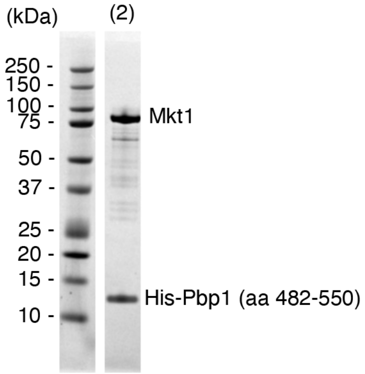


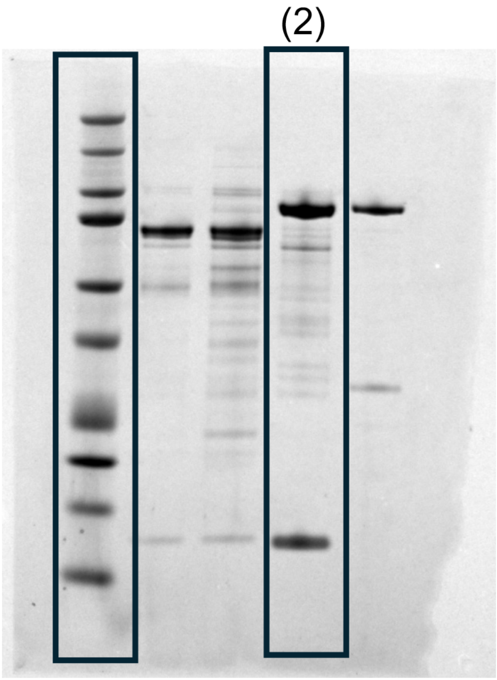

Figure 2C, Additional experiment

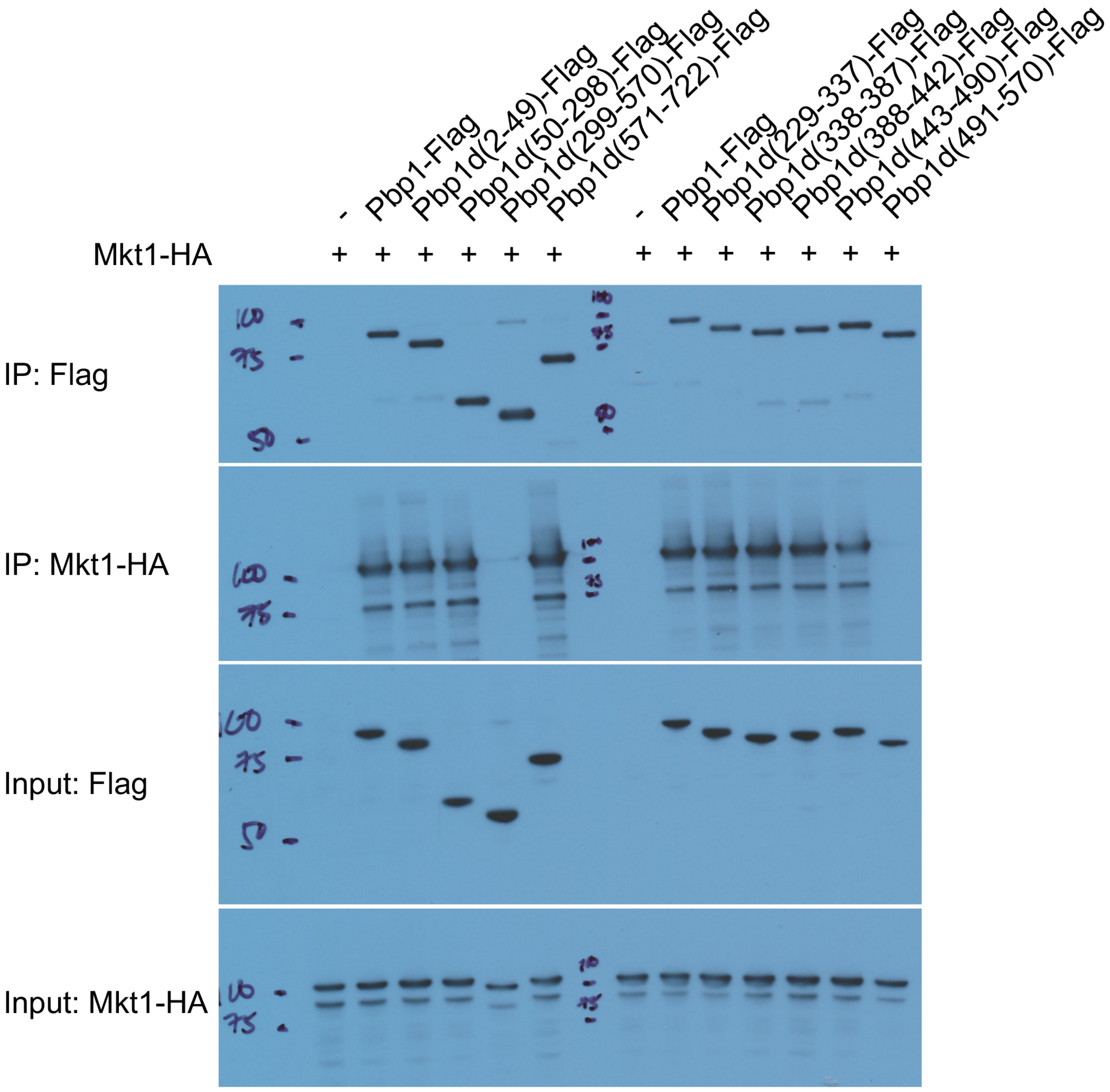

Figure 2F, Additional experiment

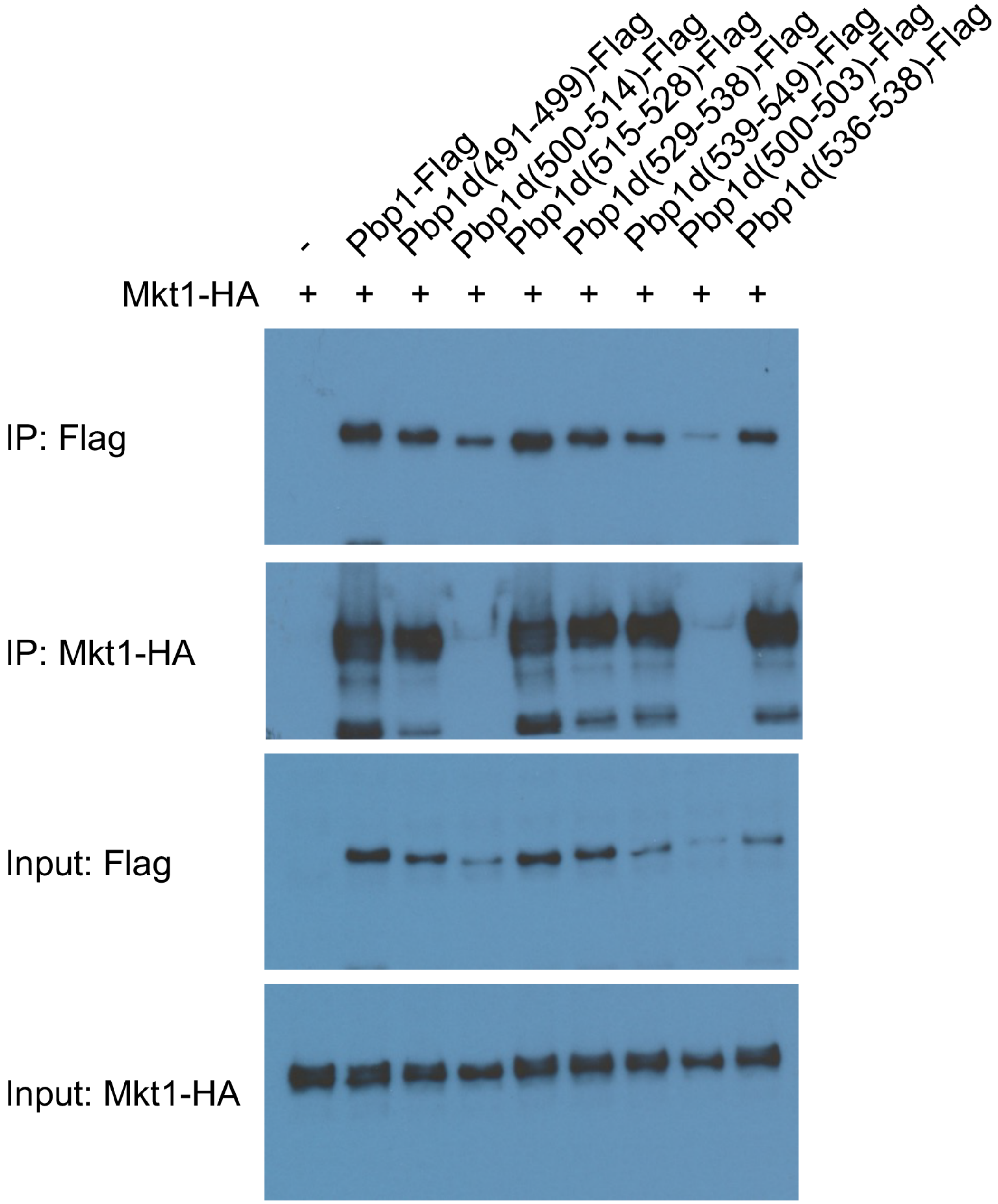

Supplement: SourceData F2 — is the source file for Fig. 2. [file jcb_202411169_sourcedataf2.pdf]
