# Supplementary material for: The yeast Mkt1/Pbp1 complex promotes adaptive responses to respiratory growth
Source: J Cell Biol. 2025 Aug 13;224(10):e202411169. doi: 10.1083/jcb.202411169 (PMC12345631; doi:10.1083/jcb.202411169)

A

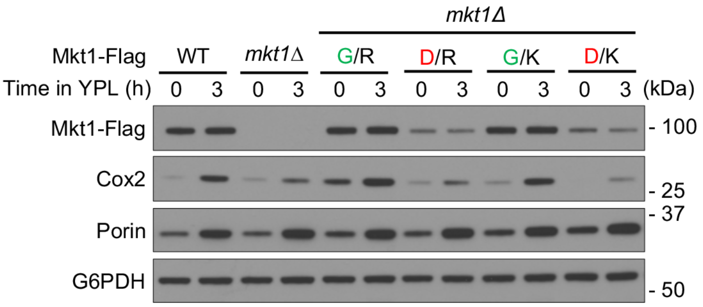

mkt1Δ
Mkt1-Flag
WT
mkt1Δ
G/R
D/R
G/K
D/K
Time in YPL (h)
0
3
(kDa)
Mkt1-Flag
- 100
Cox2
- 25
- 37
Porin
G6PDH
- 50

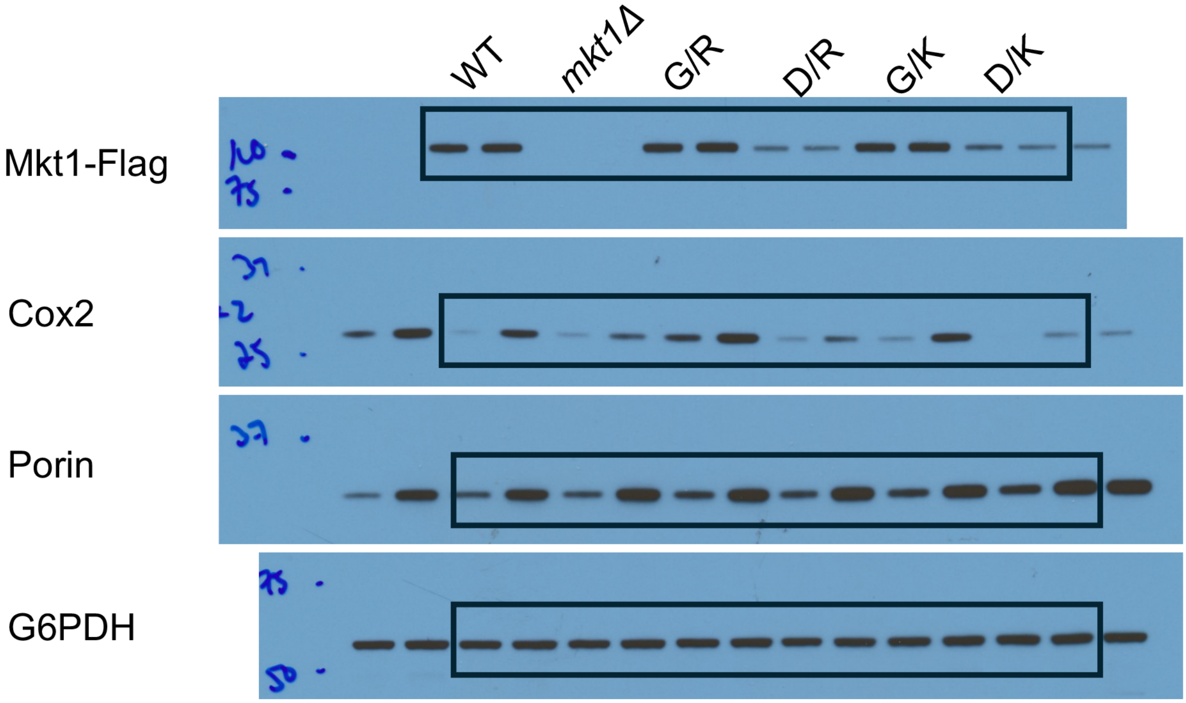

WT
mkt1Δ
G/R
D/R
G/K
D/K
Mkt1-Flag
Cox2
Porin
G6PDH

C

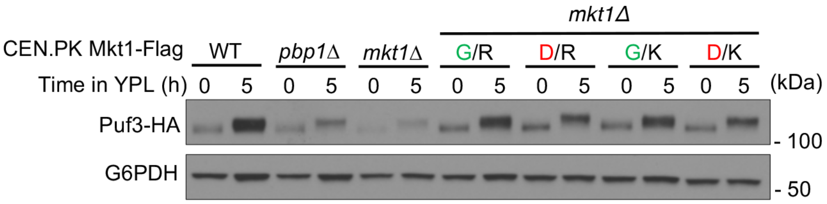
mkt1Δ
CEN.PK Mkt1-Flag
WT
pbp1Δ
mkt1Δ
G/R
D/R
G/K
D/K
Time in YPL (h)
0 5 0 5 0 5 0 5 0 5 0 5 0 5
(kDa)
Puf3-HA
- 100
G6PDH
- 50

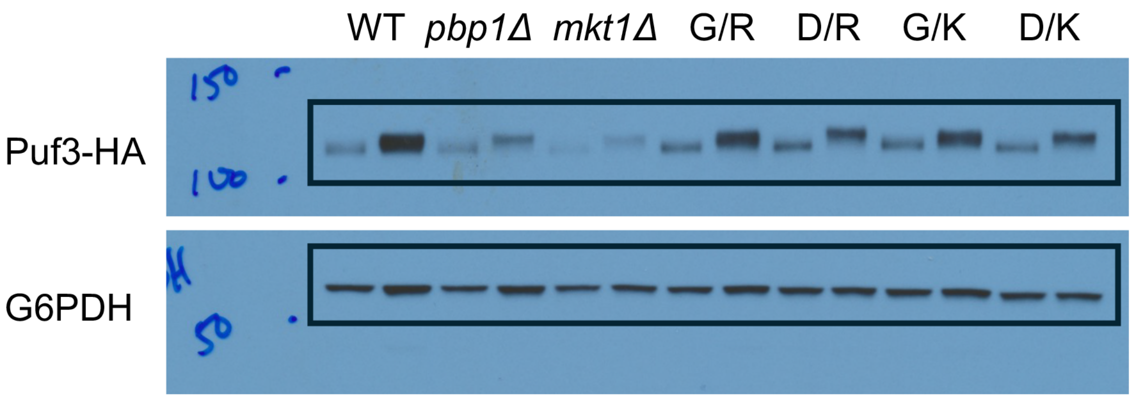
WT
pbp1Δ
mkt1Δ
G/R
D/R
G/K
D/K
Puf3-HA
150
100
G6PDH
50

D

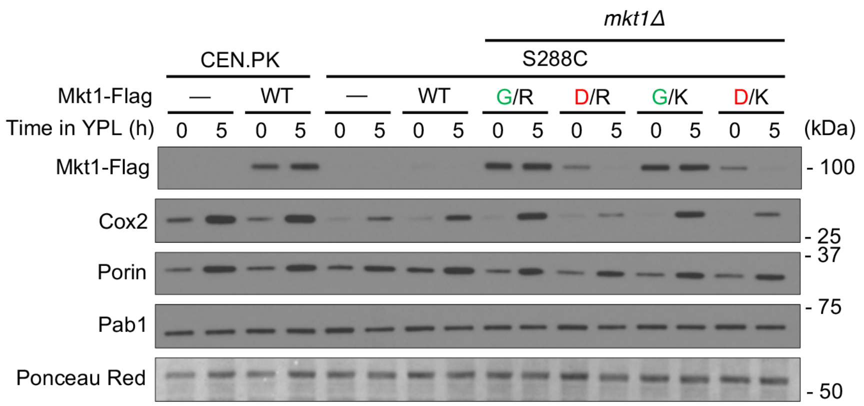

mkt1Δ
CEN.PK
S288C
Mkt1-Flag — WT — WT G/R D/R G/K D/K
Time in YPL (h) 0 5 0 5 0 5 0 5 0 5 0 5 0 5 0 5
(kDa)
Mkt1-Flag
- 100
Cox2
- 25
Porin
- 37
Pab1
- 75
Ponceau Red
- 50

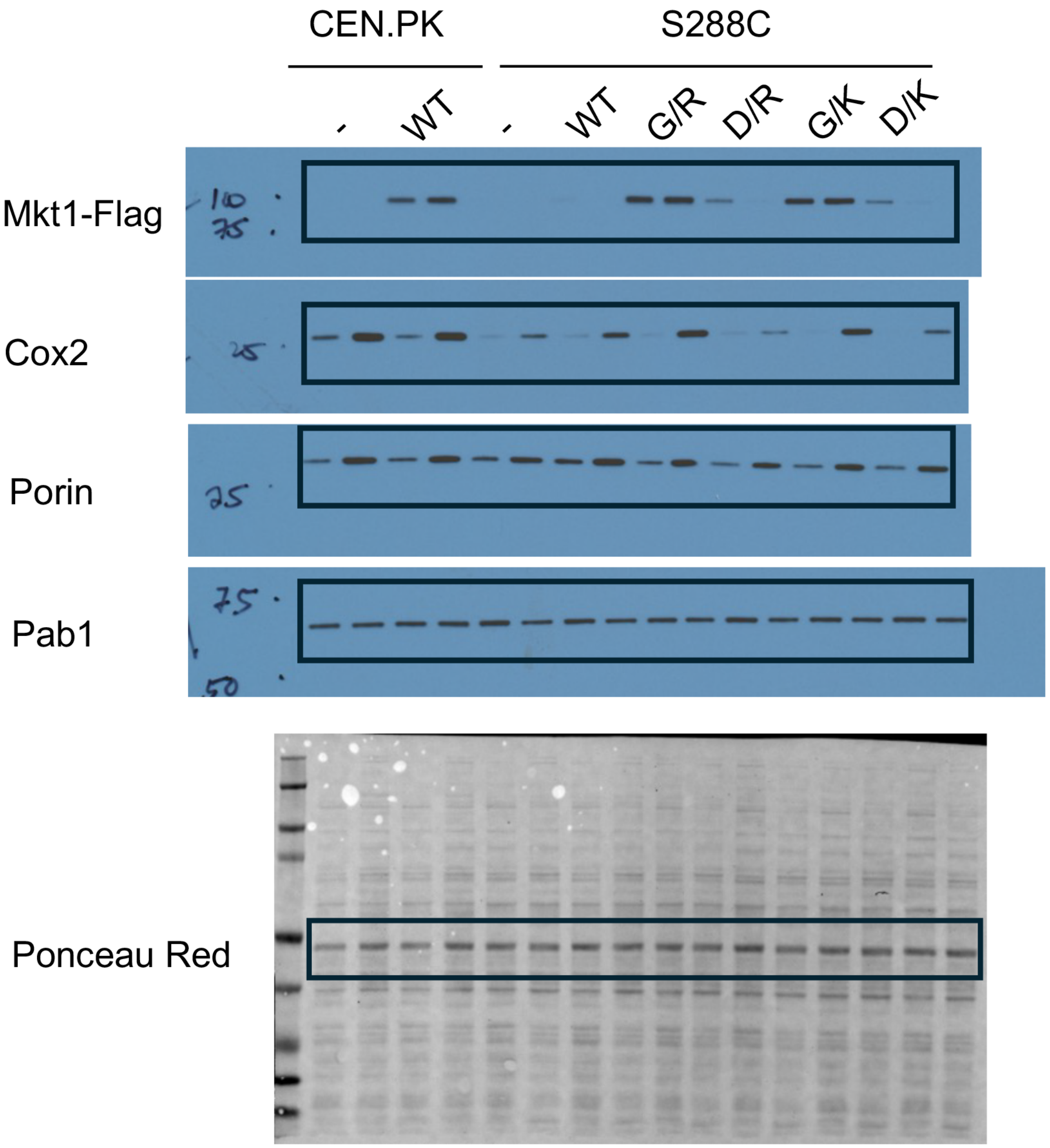

CEN.PK
S288C
- WT - WT G/R D/R G/K D/K
Mkt1-Flag
Cox2
Porin
Pab1
Ponceau Red

G

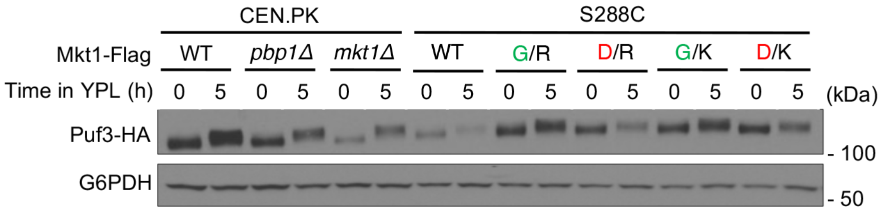
CEN.PK
S288C
Mkt1-Flag
WT
pbp1Δ
mkt1Δ
WT
G/R
D/R
G/K
D/K
Time in YPL (h)
0 5 0 5 0 5 0 5 0 5 0 5 0 5 0 5
(kDa)
Puf3-HA
- 100
G6PDH
- 50

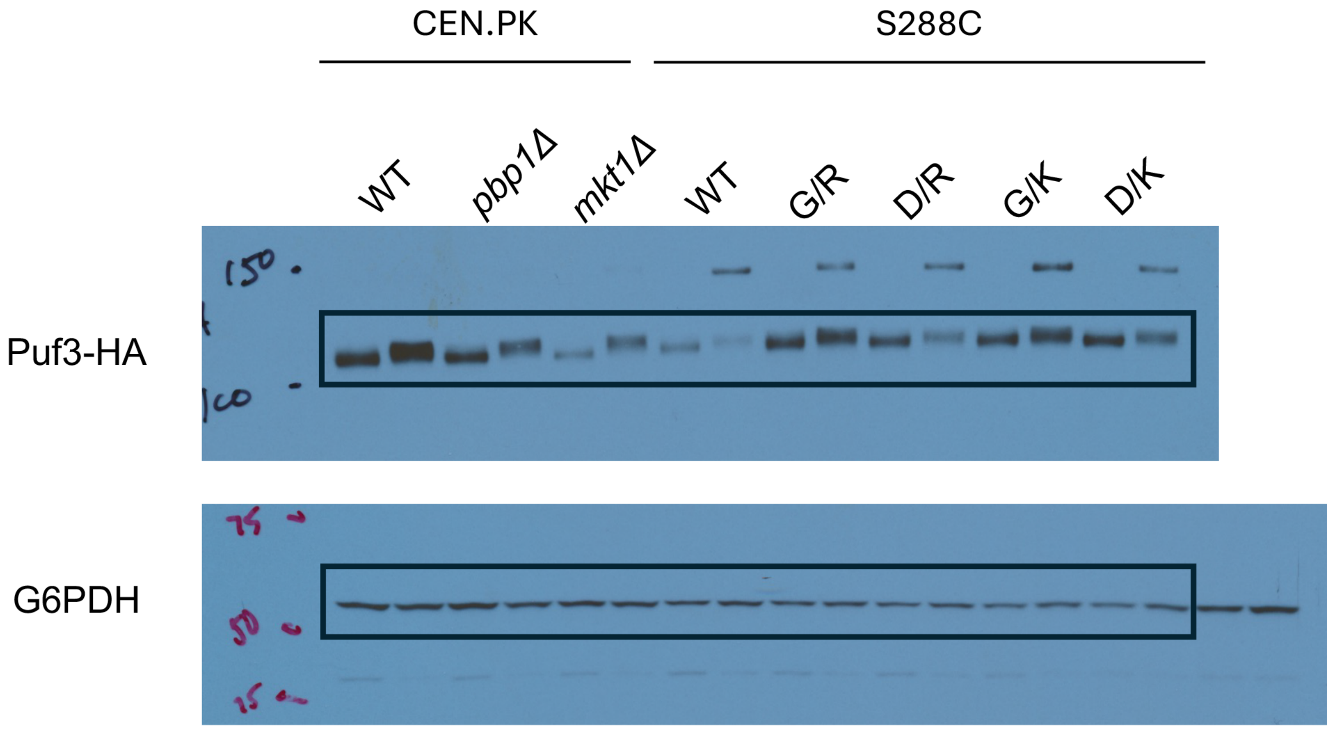
CEN.PK
S288C
WT
pbp1Δ
mkt1Δ
WT
G/R
D/R
G/K
D/K
Puf3-HA
G6PDH
150
100
75
50
25

Figure 8A, Additional experiment

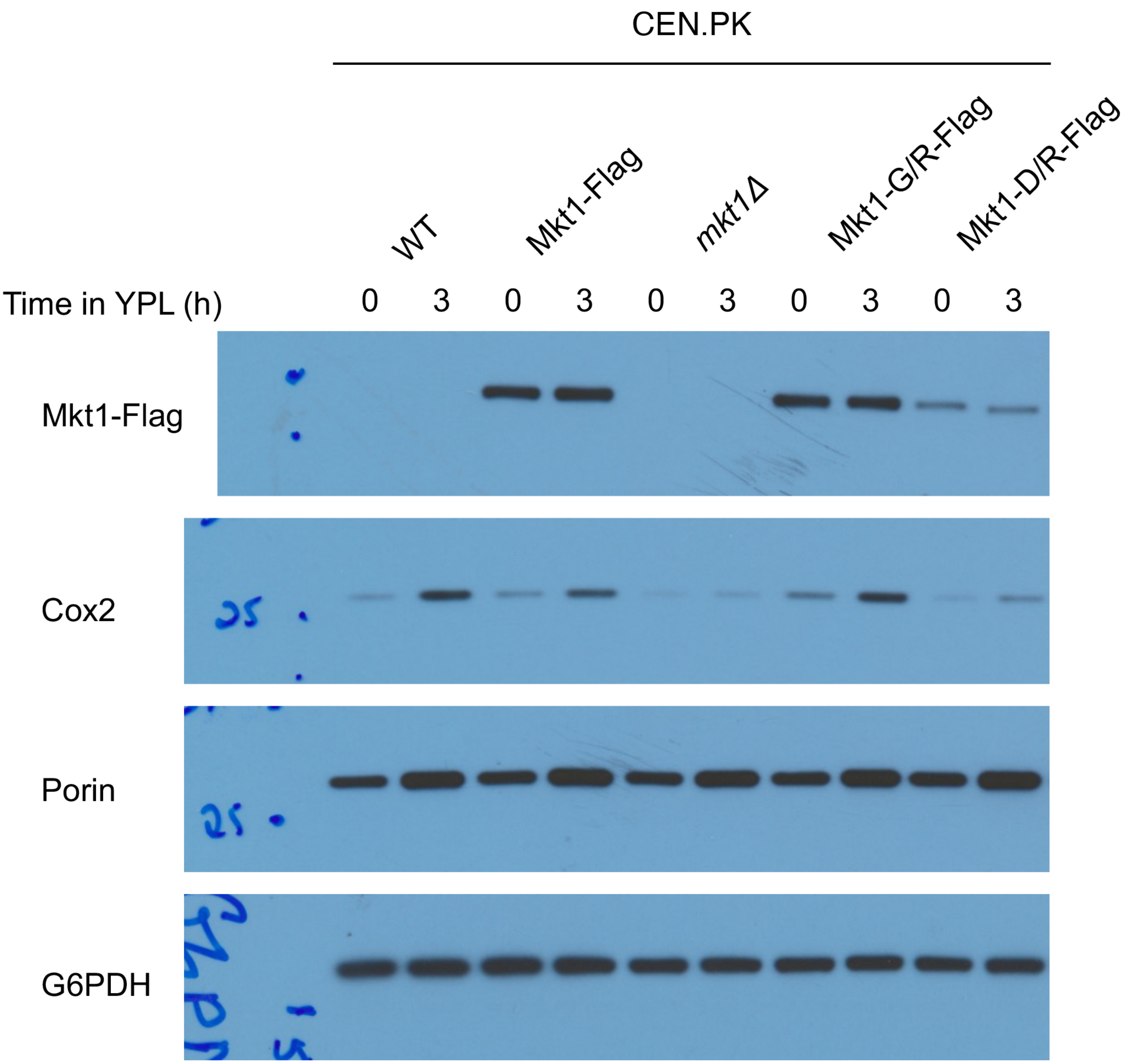

Figure 8D, Additional experiment

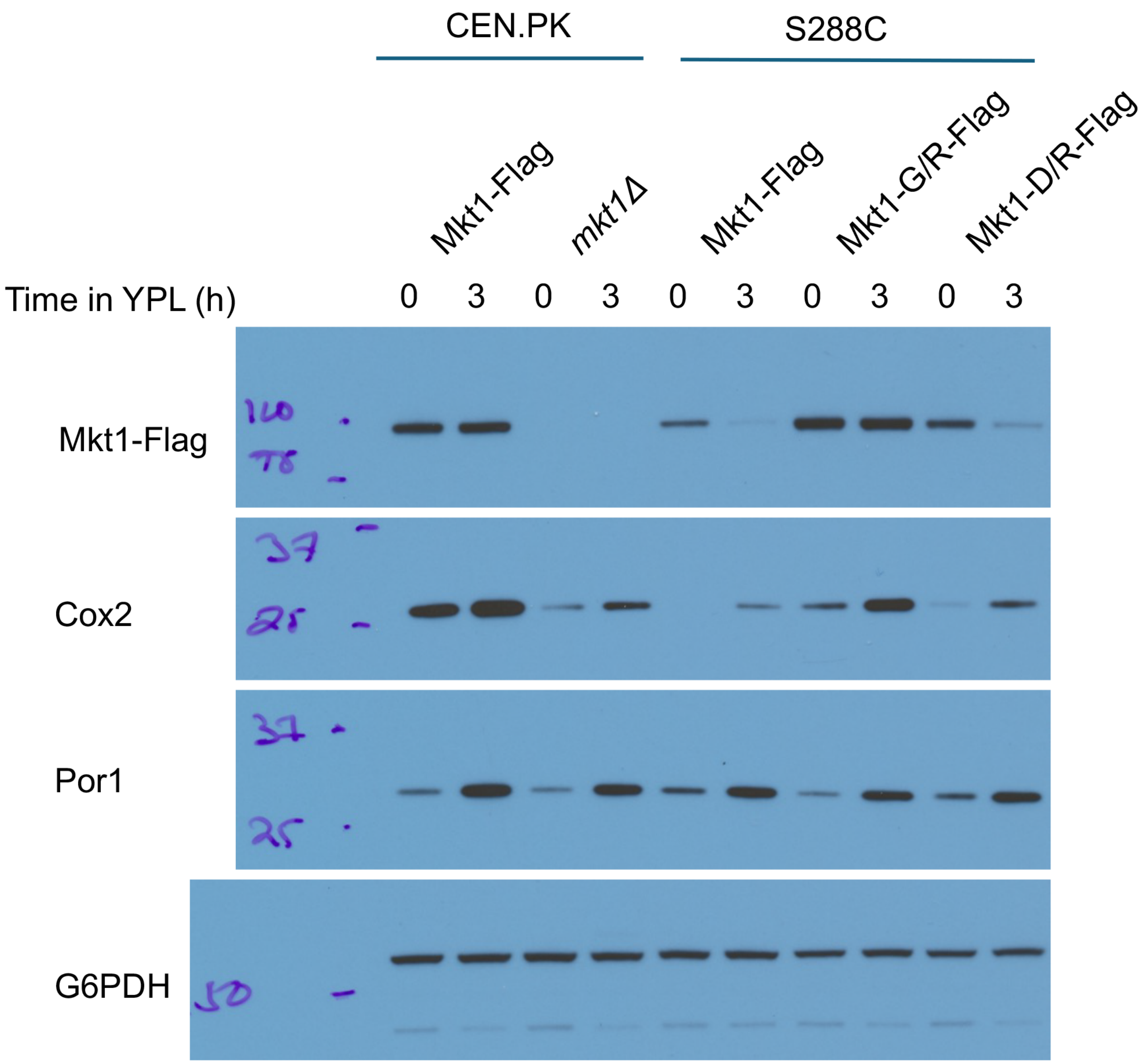

Figure 8G, Additional experiment

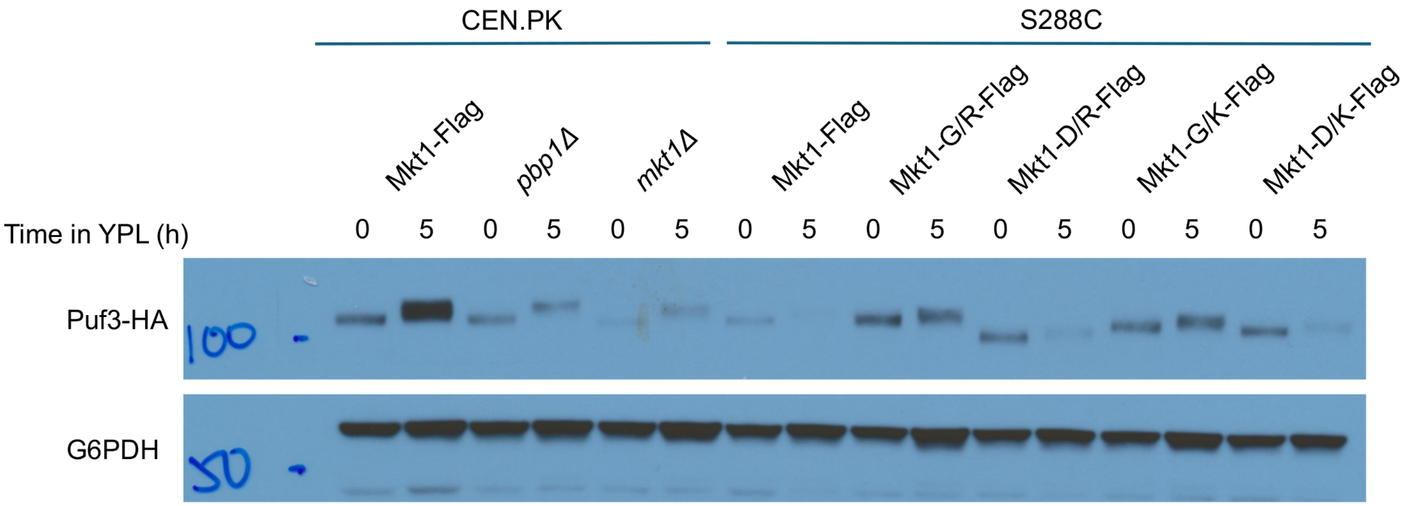

Supplement: SourceData F8 — is the source file for Fig. 8. [file jcb_202411169_sourcedataf8.pdf]
