# Supplementary material for: The yeast Mkt1/Pbp1 complex promotes adaptive responses to respiratory growth
Source: J Cell Biol. 2025 Aug 13;224(10):e202411169. doi: 10.1083/jcb.202411169 (PMC12345631; doi:10.1083/jcb.202411169)

A

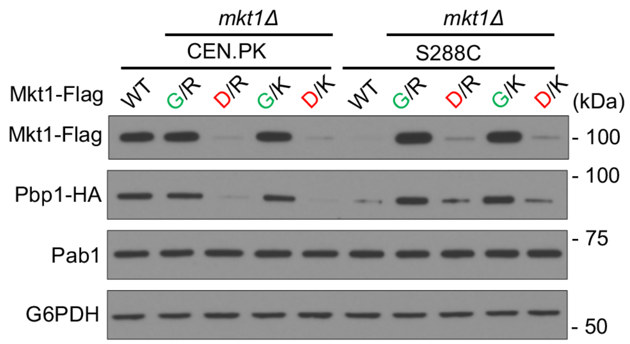
mkt1Δ
CEN.PK
mkt1Δ
S288C
Mkt1-Flag
WT G/R D/R G/K D/K WT G/R D/R G/K D/K
(kDa)
Mkt1-Flag
- 100
Pbp1-HA
- 100
Pab1
- 75
G6PDH
- 50

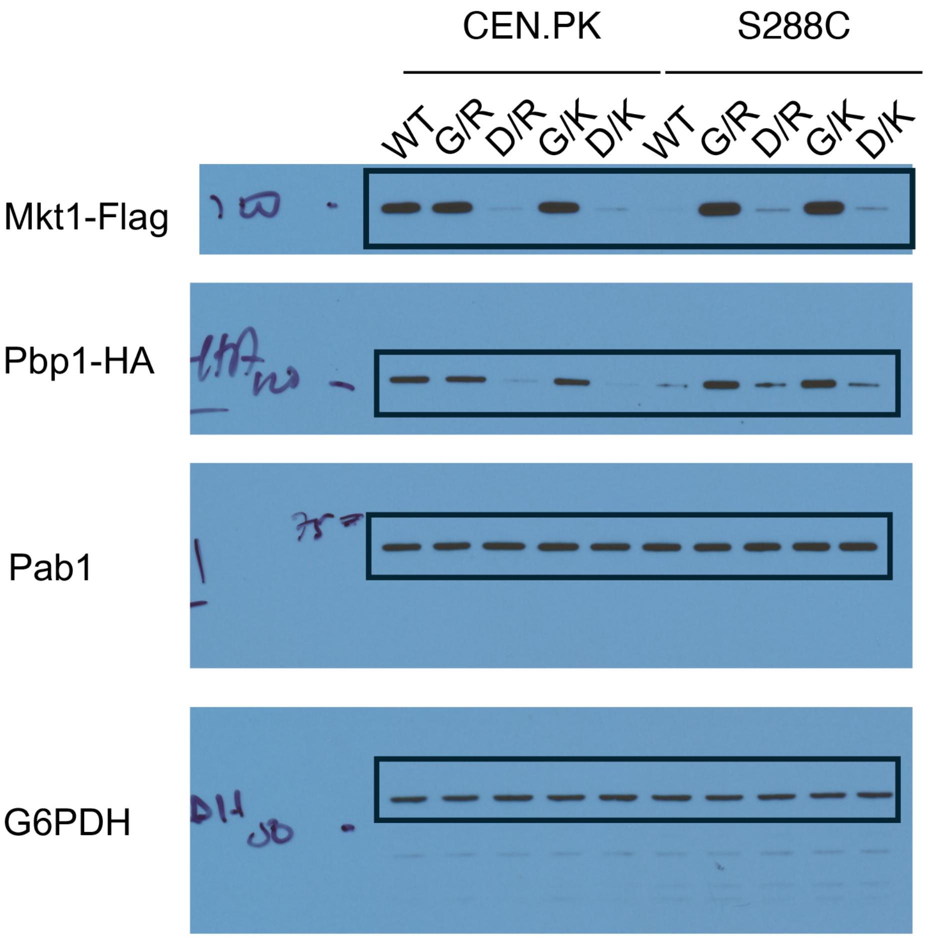
CEN.PK
S288C
WT G/R D/R G/K D/K WT G/R D/R G/K D/K
Mkt1-Flag
Pbp1-HA
Pab1
G6PDH

B

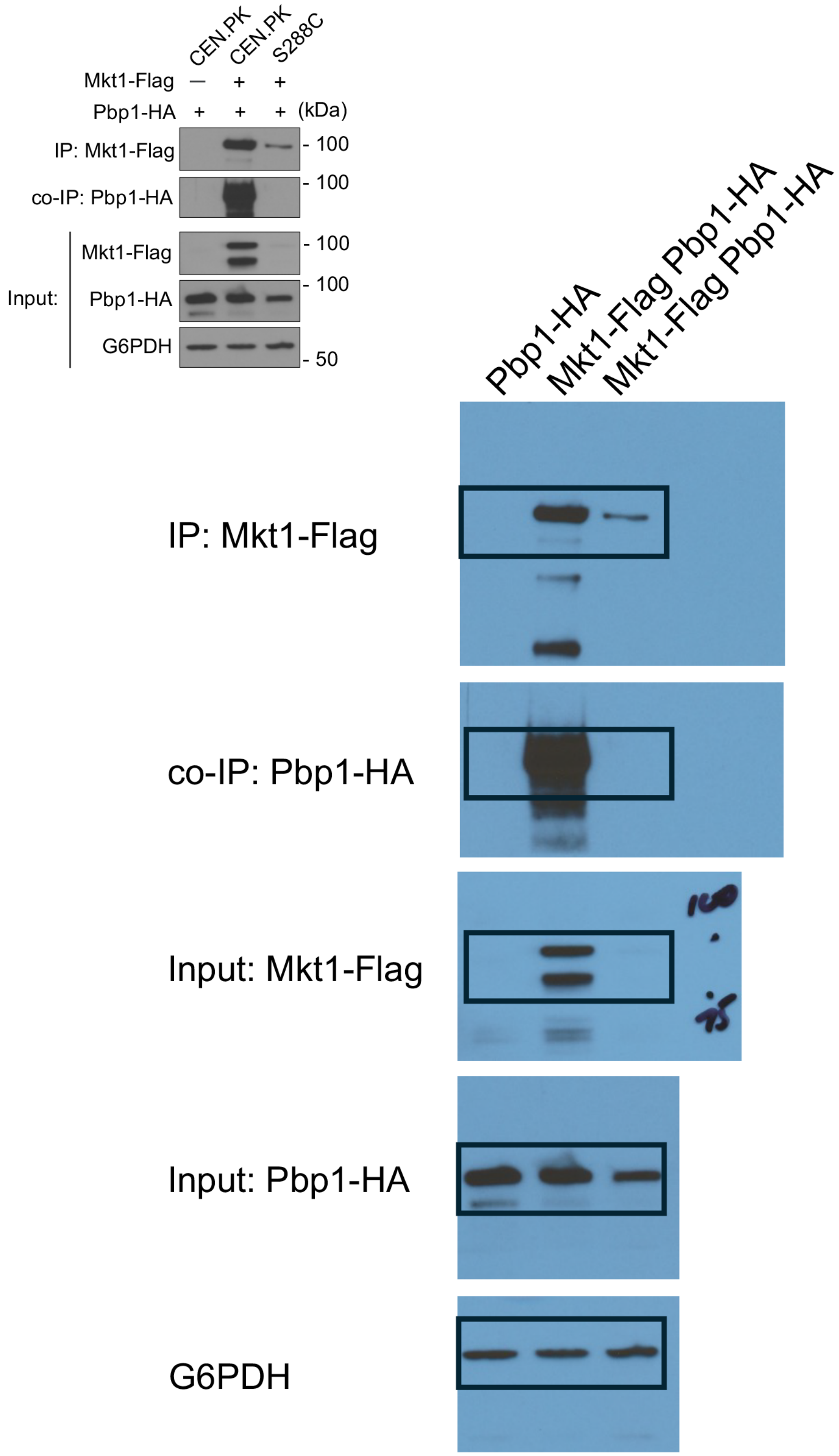

CEN.PK
CEN.PK
S288C
Mkt1-Flag — + +
Pbp1-HA + + + (kDa)
IP: Mkt1-Flag
co-IP: Pbp1-HA
Input:
Mkt1-Flag
Pbp1-HA
G6PDH
- 100
- 100
- 100
- 100
- 50
Pbp1-HA
Mkt1-Flag Pbp1-HA
Mkt1-Flag Pbp1-HA
IP: Mkt1-Flag
co-IP: Pbp1-HA
Input: Mkt1-Flag
Input: Pbp1-HA
G6PDH
100
75

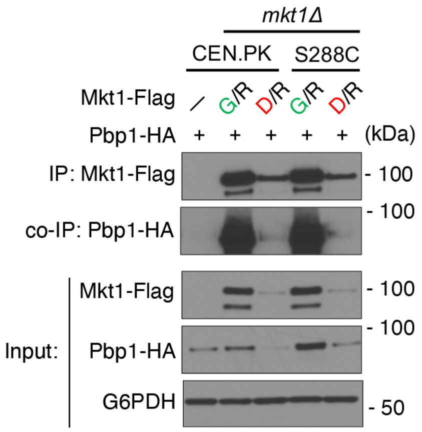
mkt1Δ
CEN.PK
S288C
Mkt1-Flag
/
G/R
D/R
G/R
D/R
Pbp1-HA
+ + + + +
(kDa)
IP: Mkt1-Flag
- 100
co-IP: Pbp1-HA
- 100
Mkt1-Flag
- 100
Input:
Pbp1-HA
- 100
G6PDH
- 50

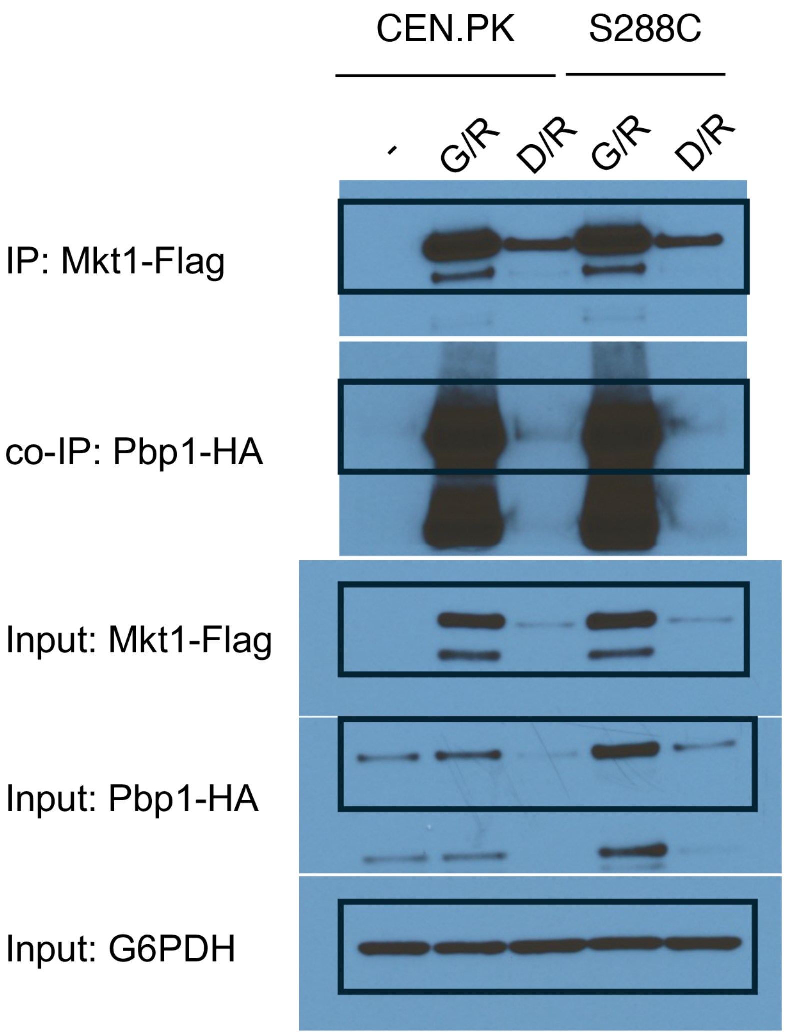
CEN.PK
S288C
-
G/R
D/R
G/R
D/R
IP: Mkt1-Flag
co-IP: Pbp1-HA
Input: Mkt1-Flag
Input: Pbp1-HA
Input: G6PDH

C

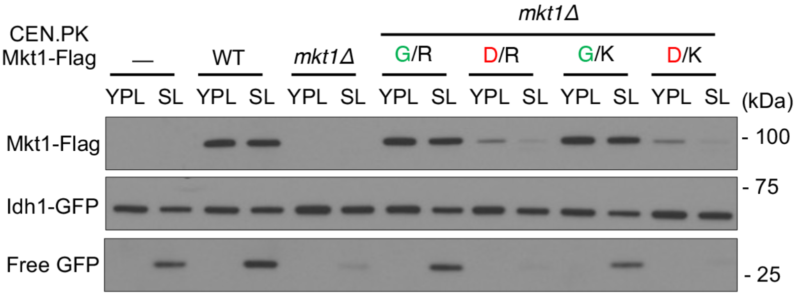

mkt1Δ
CEN.PK
Mkt1-Flag
—
WT
mkt1Δ
G/R
D/R
G/K
D/K
YPL SL YPL SL YPL SL YPL SL YPL SL YPL SL YPL SL
(kDa)
Mkt1-Flag
Idh1-GFP
Free GFP
- 100
- 75
- 25

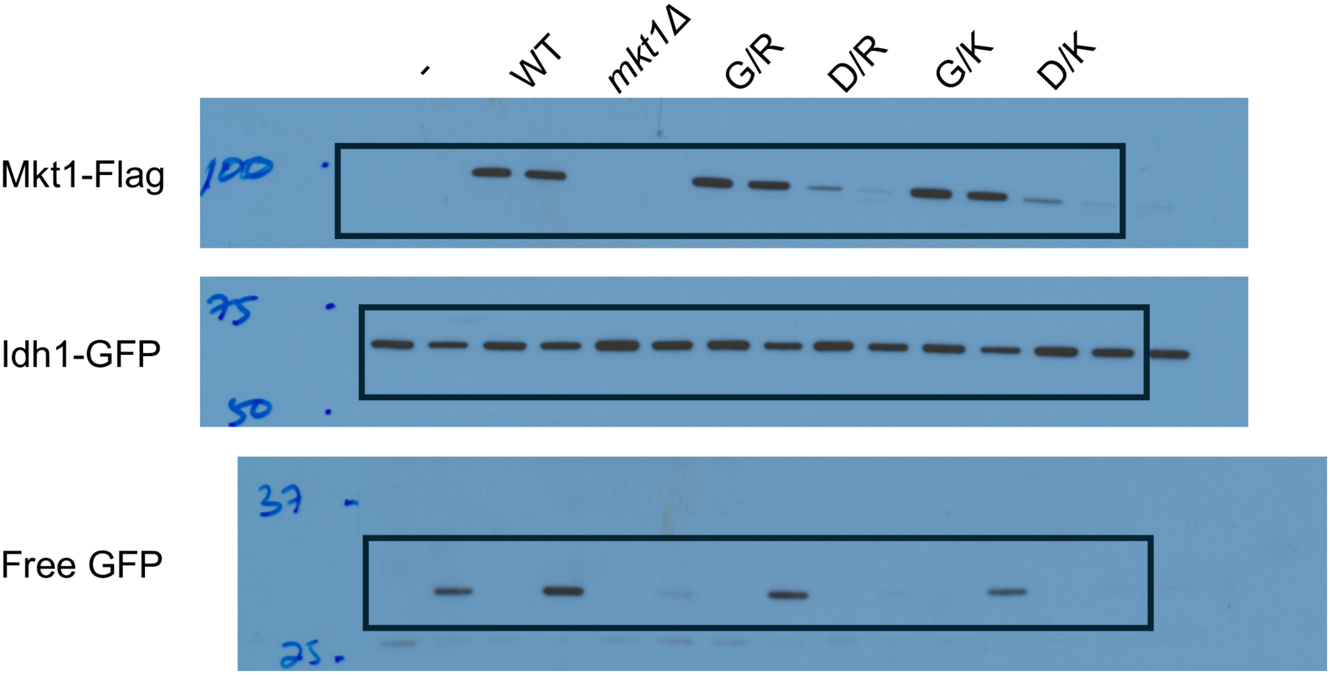

-
WT
mkt1Δ
G/R
D/R
G/K
D/K
Mkt1-Flag
Idh1-GFP
Free GFP
100
75
50
37
25

E

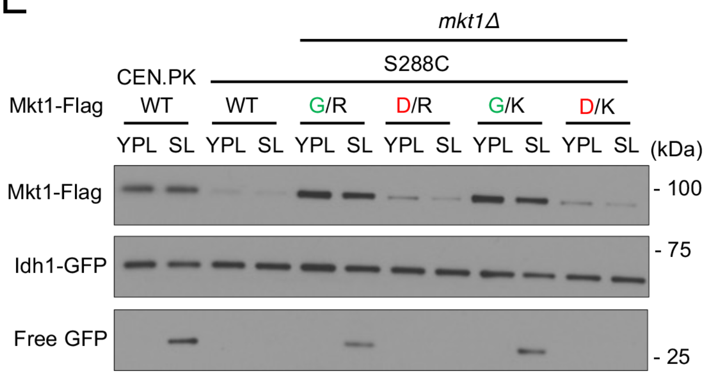
mkt1Δ
S288C
CEN.PK
Mkt1-Flag
WT
WT
G/R
D/R
G/K
D/K
YPL SL YPL SL YPL SL YPL SL YPL SL YPL SL
(kDa)
Mkt1-Flag
- 100
Idh1-GFP
- 75
Free GFP
- 25

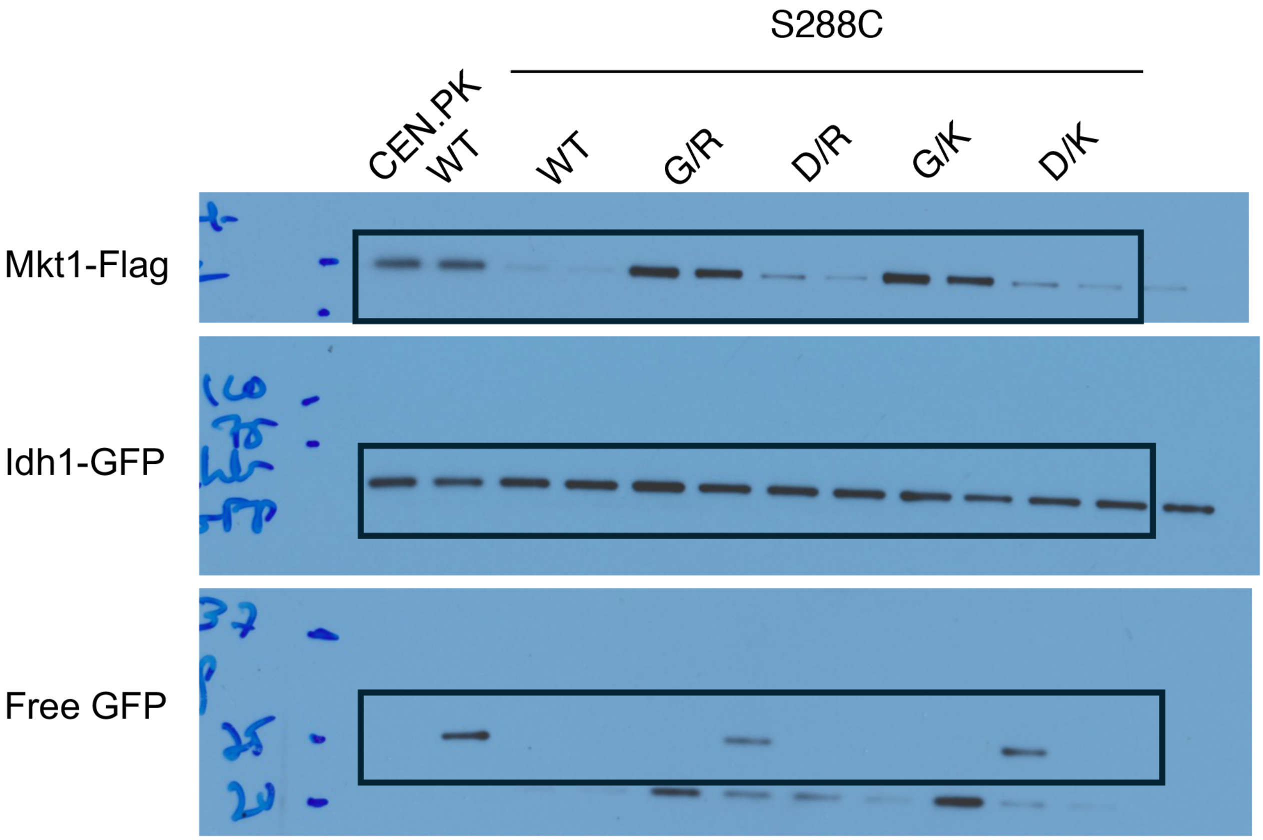
S288C
CEN.PK WT
WT
G/R
D/R
G/K
D/K
Mkt1-Flag
Idh1-GFP
Free GFP

G

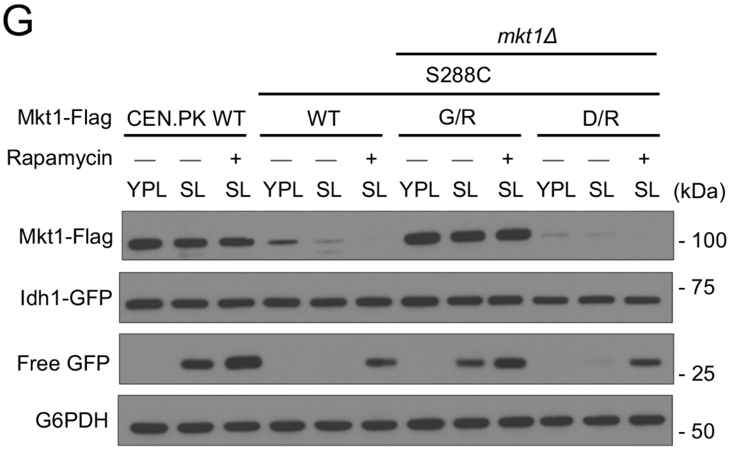
mkt1Δ
S288C
Mkt1-Flag CEN.PK WT WT G/R D/R
Rapamycin — — + — — + — — + — — +
YPL SL SL YPL SL SL YPL SL SL YPL SL SL (kDa)
Mkt1-Flag
Idh1-GFP
Free GFP
G6PDH
- 100
- 75
- 25
- 50

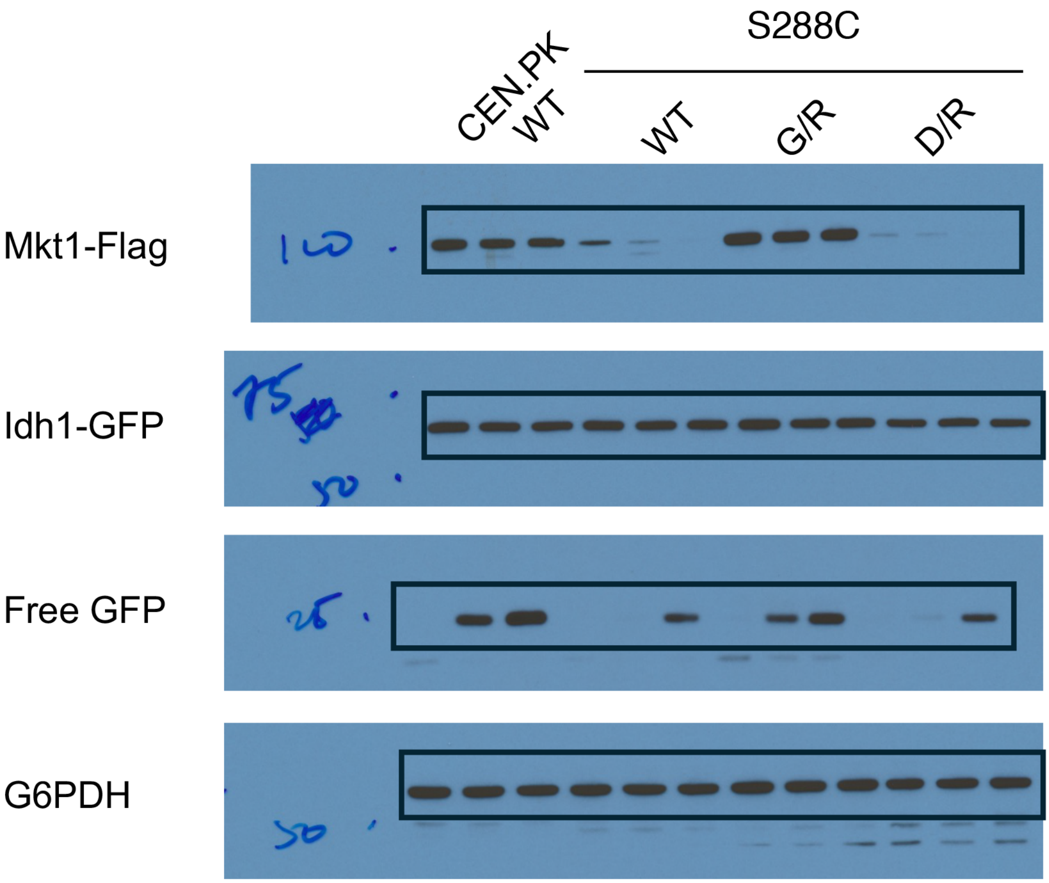
S288C
CEN.PK WT
WT
G/R
D/R
Mkt1-Flag
Idh1-GFP
Free GFP
G6PDH

H

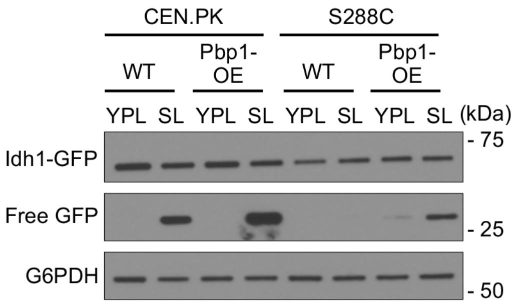
CEN.PK
S288C
WT
Pbp1-OE
WT
Pbp1-OE
YPL SL YPL SL YPL SL YPL SL
(kDa)
Idh1-GFP
Free GFP
G6PDH
- 75
- 25
- 50

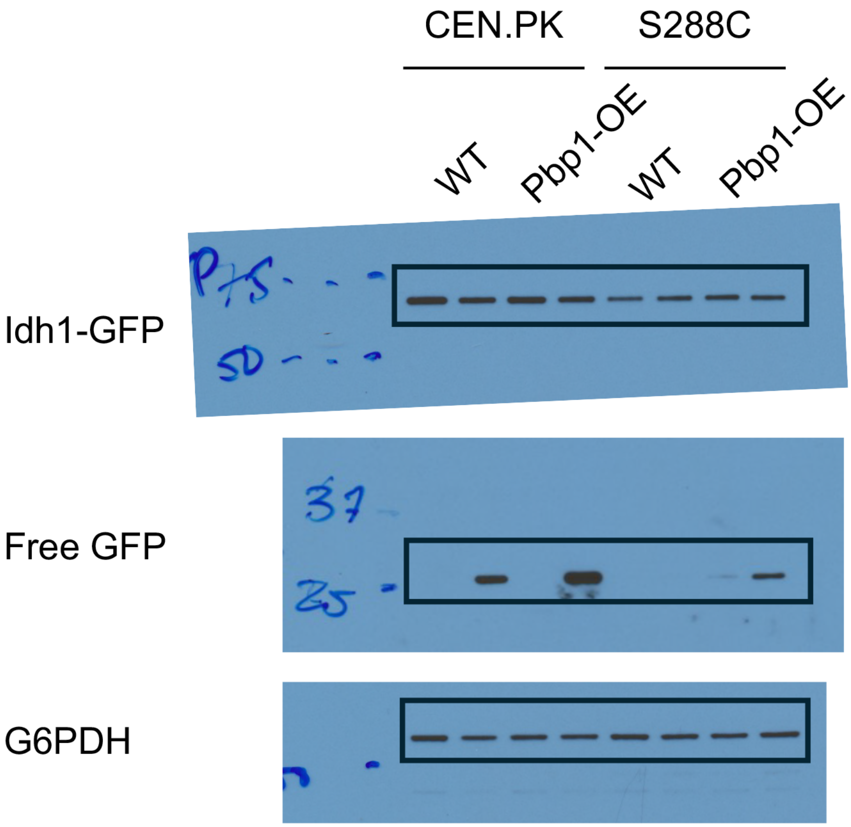
CEN.PK
S288C
WT
Pbp1-OE
WT
Pbp1-OE
Idh1-GFP
Free GFP
G6PDH
75
50
37
25

Figure 9C, Additional experiment

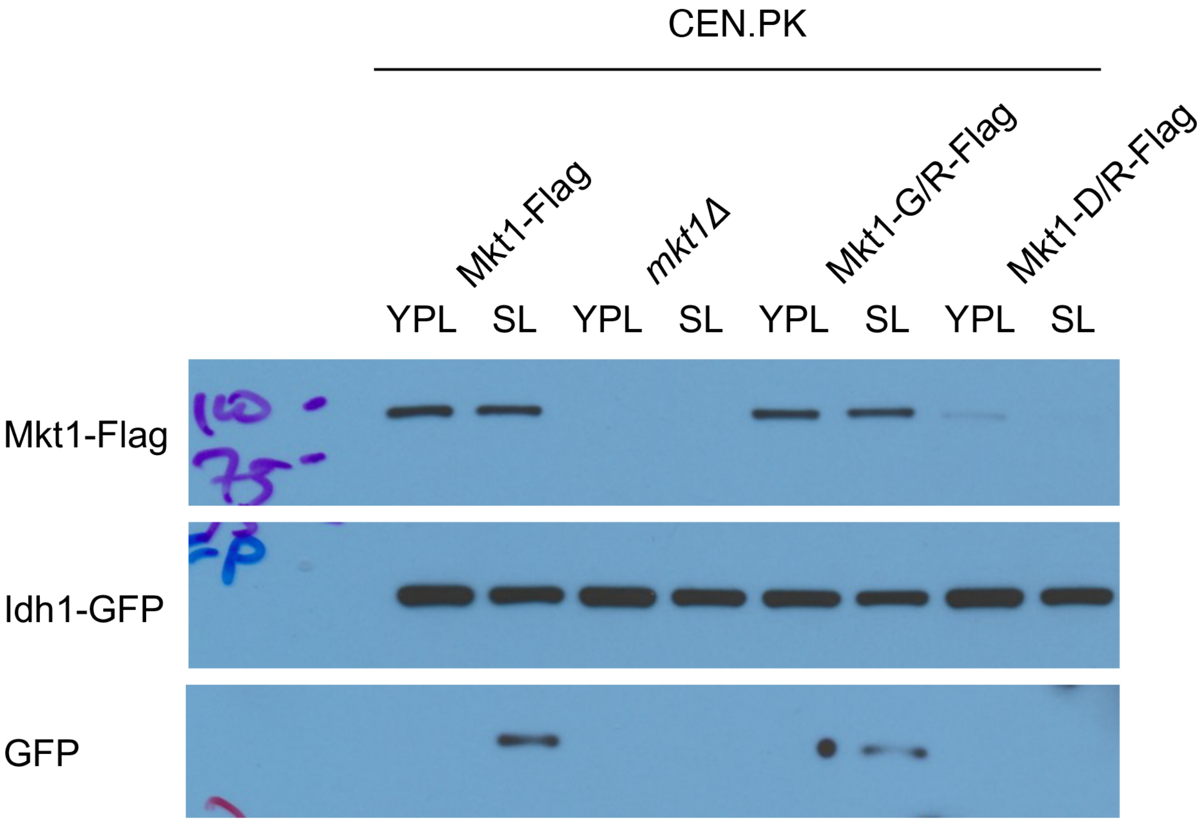

Supplement: SourceData F9 — is the source file for Fig. 9. [file jcb_202411169_sourcedataf9.pdf]
