# Supplementary material for: The yeast Mkt1/Pbp1 complex promotes adaptive responses to respiratory growth
Source: J Cell Biol. 2025 Aug 13;224(10):e202411169. doi: 10.1083/jcb.202411169 (PMC12345631; doi:10.1083/jcb.202411169)

B

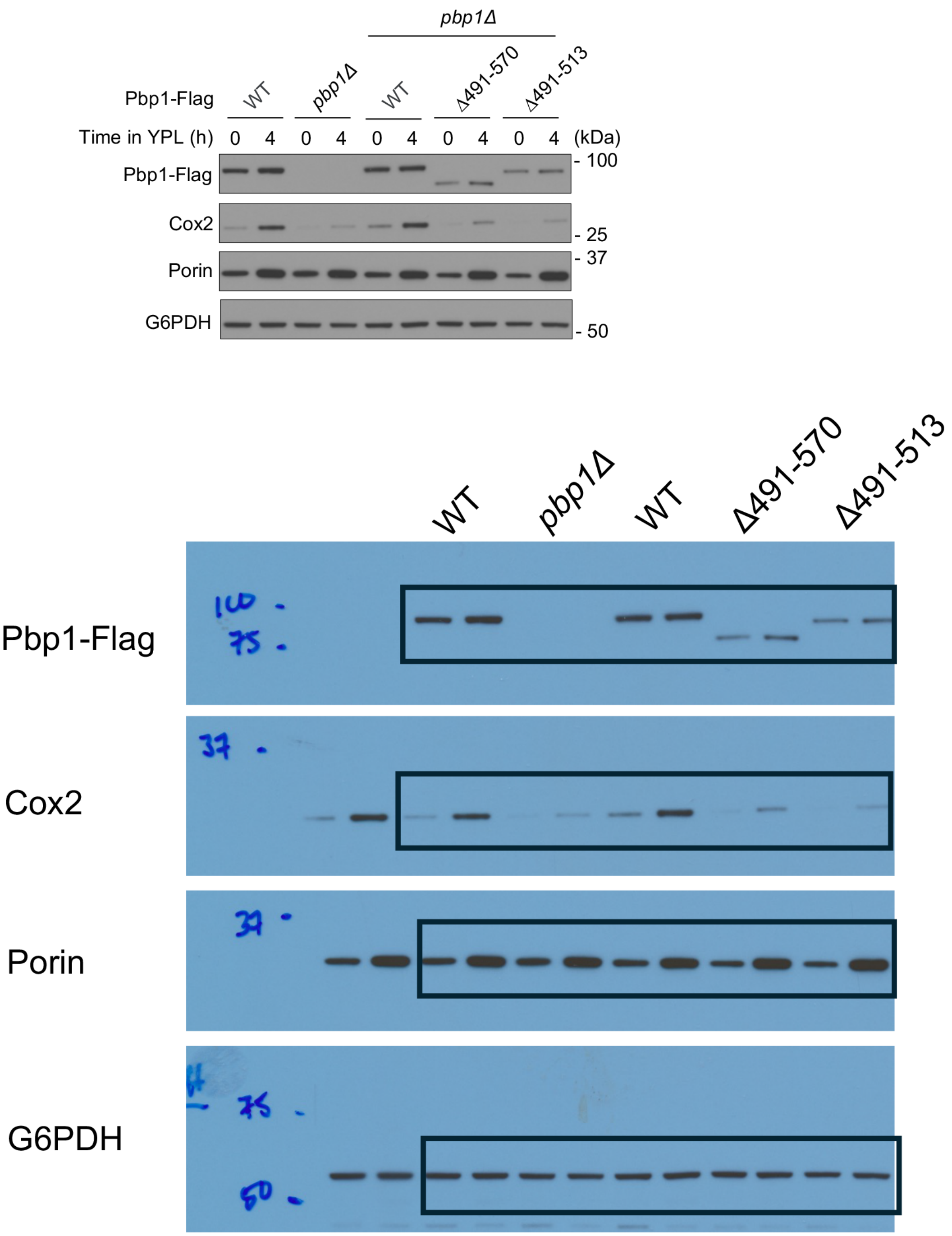
pbp1Δ
Pbp1-Flag
WT
pbp1Δ
WT
Δ491-570
Δ491-513
Time in YPL (h)
0 4 0 4 0 4 0 4 0 4
(kDa)
Pbp1-Flag
Cox2
Porin
G6PDH
- 100
- 25
- 37
- 50
WT
pbp1Δ
WT
Δ491-570
Δ491-513
Pbp1-Flag
Cox2
Porin
G6PDH

D

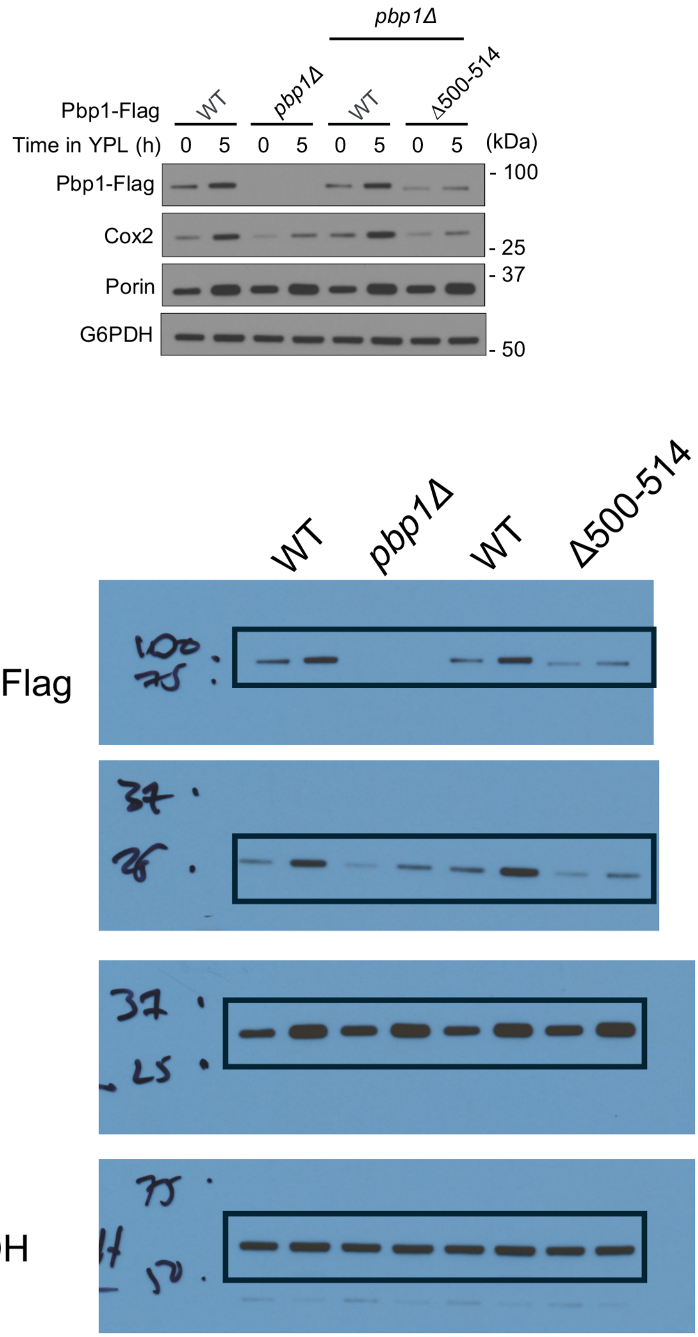

pbp1Δ
Pbp1-Flag
WT
pbp1Δ
WT
Δ500-514
Time in YPL (h)
0 5 0 5 0 5 0 5
(kDa)
Pbp1-Flag
- 100
Cox2
- 25
Porin
- 37
G6PDH
- 50
WT
pbp1Δ
WT
Δ500-514
Pbp1-Flag
Cox2
Porin
G6PDH

F

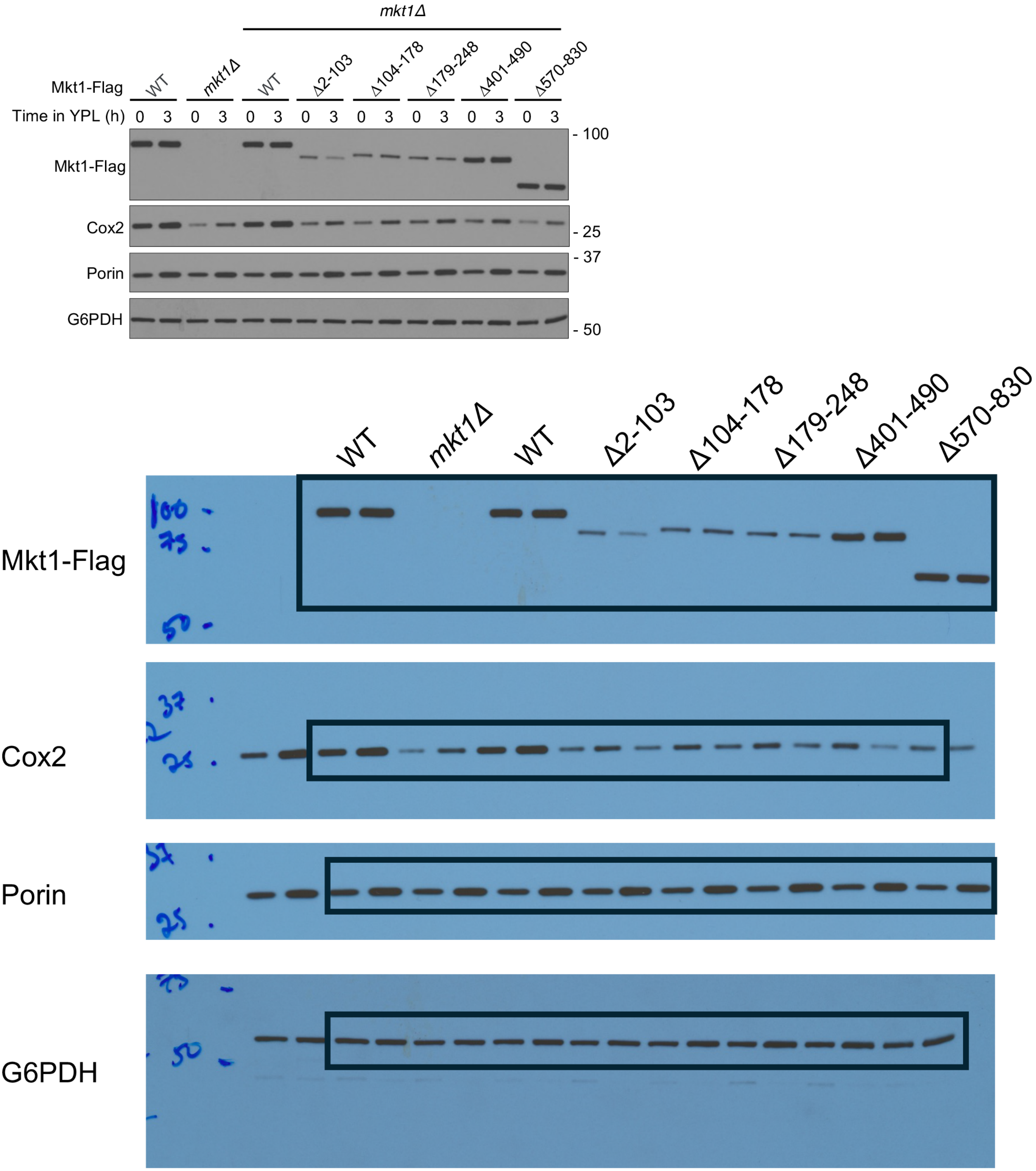

mkt1Δ
Mkt1-Flag
WT
mkt1Δ
WT
Δ2-103
Δ104-178
Δ179-248
Δ401-490
Δ570-830
Time in YPL (h)
0 3 0 3 0 3 0 3 0 3 0 3 0 3 0 3
Mkt1-Flag
Cox2
Porin
G6PDH
- 100
- 25
- 37
- 50
WT
mkt1Δ
WT
Δ2-103
Δ104-178
Δ179-248
Δ401-490
Δ570-830
Mkt1-Flag
Cox2
Porin
G6PDH
100
75
50
37
25
37
25
75
50

Figure S3B, Additional experiment

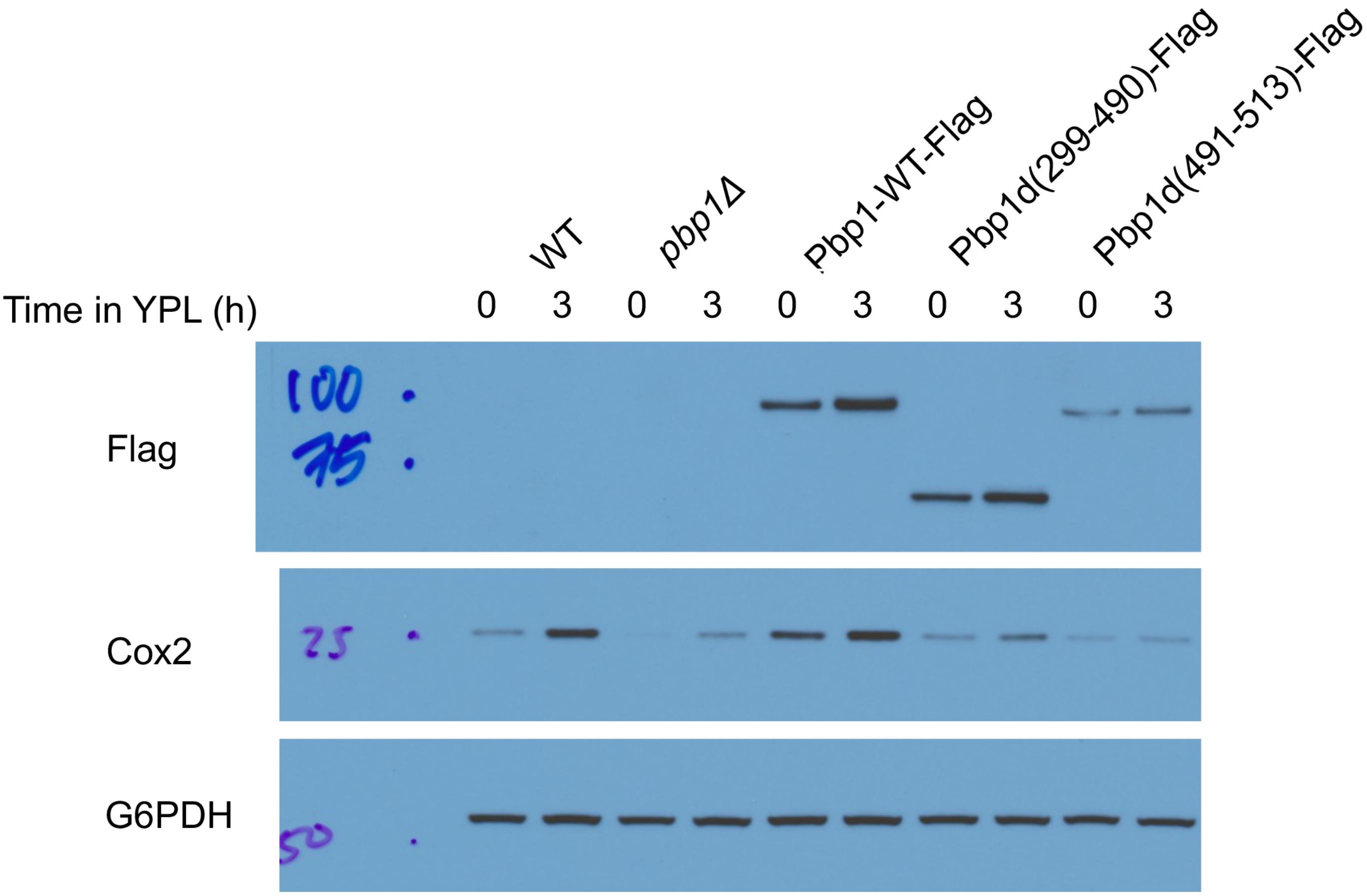

Figure S3D, Additional experiment

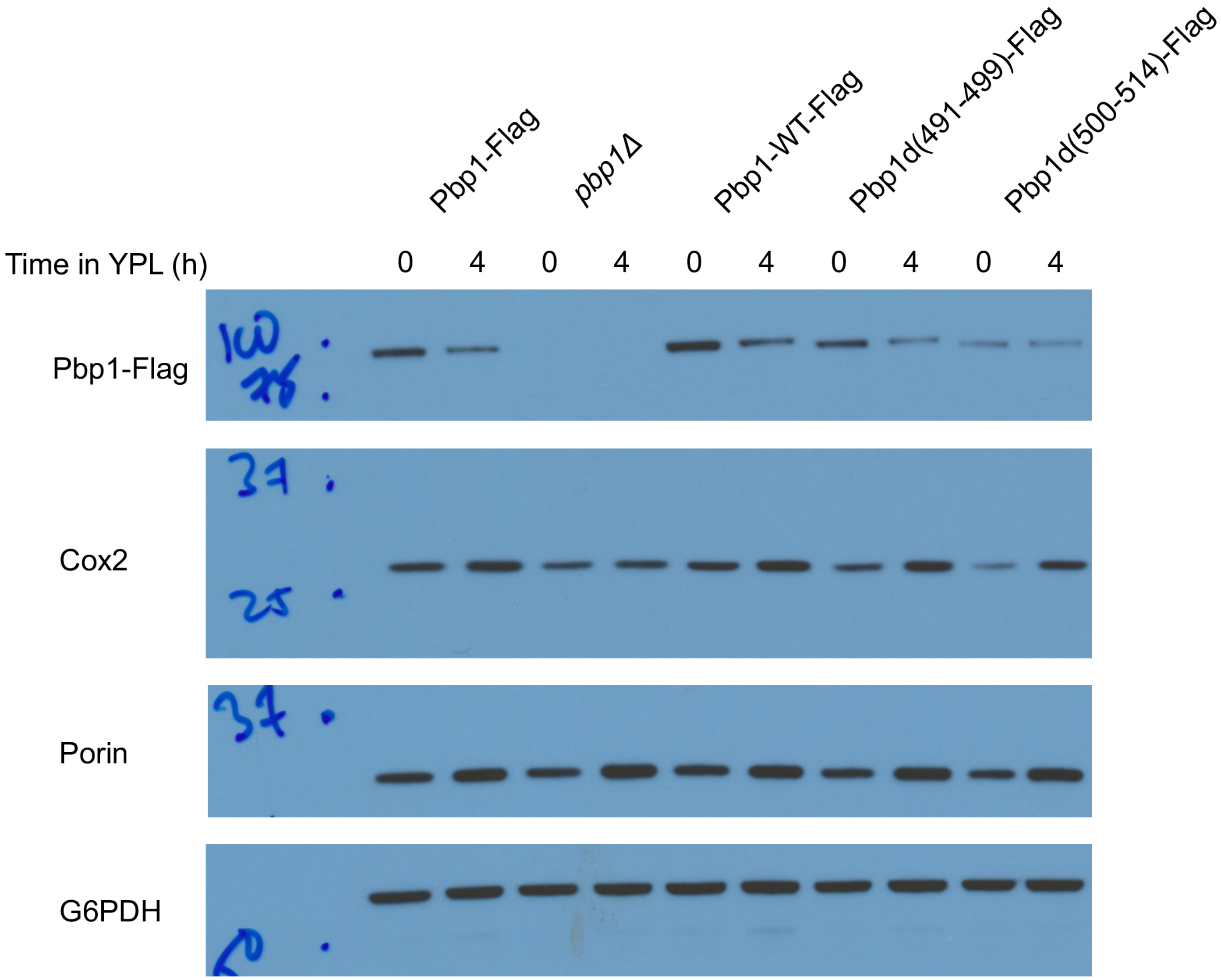

Figure S3F, Additional experiment

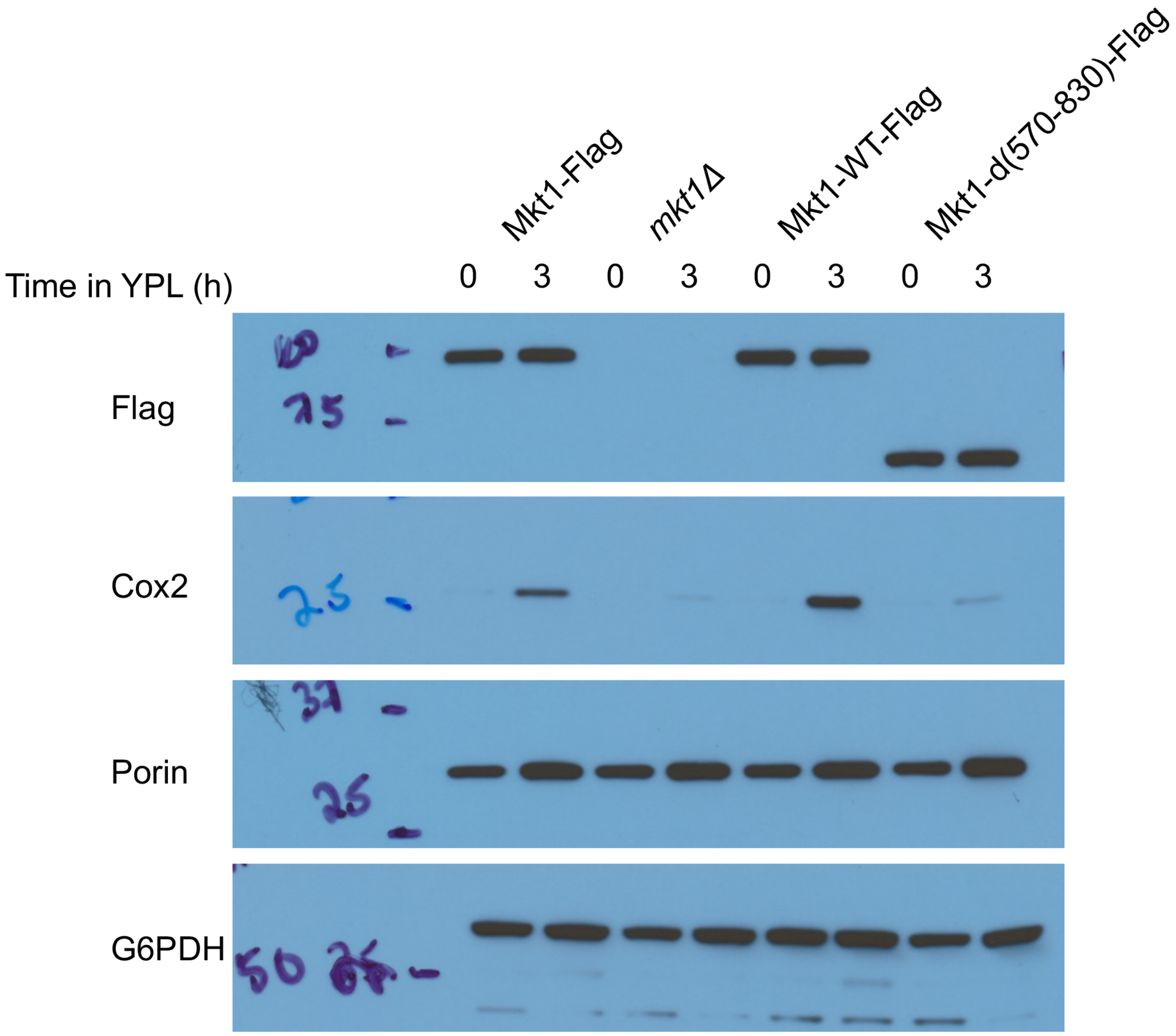

Supplement: SourceData FS3 — is the source file for Fig. S3. [file jcb_202411169_sourcedatafs3.pdf]
